# Supplementary material for: Injectable Peptide Hydrogel Encapsulation of Mesenchymal Stem Cells Improved Viability, Stemness, Anti-Inflammatory Effects, and Early Stage Wound Healing
Source: Biomolecules. 2022 Sep 17;12(9):1317. doi: 10.3390/biom12091317 (PMC9496061; doi:10.3390/biom12091317)
Supplement: Supplementary file 1 [file biomolecules-12-01317-s001.zip › biomolecules-1854688-supplementary.pdf]

Supplementary Table S1. Original MS data on proteins detected in CM from hADMSCs in 2D or encapsulated in 0.3% PGmatrix or Hystem

Accession	Peptide co	Unique pe	Confidenc	Anova (p)	q Value	Max fold c	Power	Highest mean conditio	Lowest mean conditio	Mass	Description
P02768;A0A0C4DGB6;B7WNR0;H0	187	136	1000.411	8.13E-11	1.72E-08	9.346851	1	0.3PG	2D	71362.7109	Albumin OS=Homo sapiens OX=9606 GN=ALB PE=1 SV=2
P12109;A0A087X0S5	86	69	673.8036	7.23E-09	1.71E-07	5.613915	1	2D	0.3PG	109670.0622	Collagen alpha-1(VI) chain OS=Homo sapiens OX=9606 GN=COL6A1 PE=1 SV=3
P01023;F8W7L3;H0YFH1	75	61	727.5969	1.10E-08	2.03E-07	4.086497	1	0.3PG	2D	164716.9032	Alpha-2-macroglobulin OS=Homo sapiens OX=9606 GN=A2M PE=1 SV=3
P02787;H7C5E8;C9JVG0;F8WCI6;C	71	60	699.6101	3.44E-09	1.17E-07	3.758026	1	0.3PG	2D	79345.0824	Serotransferrin OS=Homo sapiens OX=9606 GN=TF PE=1 SV=3
P01024;M0QYC8;M0QXZ3;M0R1Q	71	56	745.4945	7.34E-08	6.71E-07	2.559146	1	0.3PG	2D	188688.0949	Complement C3 OS=Homo sapiens OX=9606 GN=C3 PE=1 SV=2
P02452;I3L3H7	72	55	579.8268	3.30E-06	8.55E-06	3.340125	1	2D	0.3PG	139968.1403	Collagen alpha-1(I) chain OS=Homo sapiens OX=9606 GN=COL1A1 PE=1 SV=5
P08123;A0A087WTA8	66	53	580.5634	3.81E-09	1.25E-07	2.962212	1	2D	0.3PG	129827.2687	Collagen alpha-2(I) chain OS=Homo sapiens OX=9606 GN=COL1A2 PE=1 SV=7
H0Y7Z1;H0Y4K8	44	39	450.3486	3.42E-07	1.72E-06	2.560798	1	2D	0.3PG	121743.5501	Fibronectin (Fragment) OS=Homo sapiens OX=9606 GN=FN1 PE=1 SV=1
P08670;B0YJC4;B0YJC5;A0A1B0GT	47	37	387.4374	1.60E-10	2.27E-08	4.48285	1	2D	0.3PG	53708.7907	Vimentin OS=Homo sapiens OX=9606 GN=VIM PE=1 SV=4
P11047	42	36	357.4513	5.40E-08	5.43E-07	3.656903	1	2D	0.3PG	183305.8178	Laminin subunit gamma-1 OS=Homo sapiens OX=9606 GN=LAMC1 PE=1 SV=3
Q15063;B1ALD9	44	36	426.6451	1.07E-08	2.03E-07	11.70319	1	2D	0.3PG	93941.3973	Periostin OS=Homo sapiens OX=9606 GN=POSTN PE=1 SV=2
Q15582;H0Y8L3;S4R3C6;H0Y9D7;H	45	35	450.3779	5.25E-08	5.43E-07	9.053789	1	2D	0.3PG	75308.3206	Transforming growth factor-beta-induced protein ig-h3 OS=Homo sapiens OX=9606 GN=TGFBI PE=1 SV=1
P02461;H7C435	47	33	394.5304	3.94E-07	1.86E-06	3.76457	1	2D	0.3PG	139819.0619	Collagen alpha-1(III) chain OS=Homo sapiens OX=9606 GN=COL3A1 PE=1 SV=4
P05121	44	32	394.5547	4.28E-07	1.91E-06	2.576509	1	2D	0.3PG	45117.134	Plasminogen activator inhibitor 1 OS=Homo sapiens OX=9606 GN=SERPINE1 PE=1 SV=1
P04264;A0A1W2PQU7;A0A1W2PR	40	32	394.4775	5.94E-07	2.35E-06	2.079616	1	0.3PG	2D	66209.8959	Keratin_ type II cytoskeletal 1 OS=Homo sapiens OX=9606 GN=KRT1 PE=1 SV=6
P35579;Q5BKV1	50	31	344.492	0.000155	0.000218	1.26912	1	0.3PG	HYS	227787.2475	Myosin-9 OS=Homo sapiens OX=9606 GN=MYH9 PE=1 SV=4
P12110;C9JH44;H7C0M5	36	27	393.9751	1.13E-07	8.98E-07	4.110029	1	2D	0.3PG	109777.1344	Collagen alpha-2(VI) chain OS=Homo sapiens OX=9606 GN=COL6A2 PE=1 SV=4
P08253;H3BR66;H3BV48;H3BS34;E	31	26	325.7601	2.65E-07	1.44E-06	3.386198	1	2D	0.3PG	74965.9252	72 kDa type IV collagenase OS=Homo sapiens OX=9606 GN=MMP2 PE=1 SV=2
P00450;E9PFZ2;H7C5R1;H7C5N5;D	35	26	338.7233	1.05E-09	5.59E-08	4.664765	1	0.3PG	2D	123060.7982	Ceruloplasmin OS=Homo sapiens OX=9606 GN=CP PE=1 SV=1
P07942;G3XAI2;A0A7I2V4J9;A0A7I	36	26	294.7702	7.64E-07	2.77E-06	3.122906	1	2D	0.3PG	205281.1171	Laminin subunit beta-1 OS=Homo sapiens OX=9606 GN=LAMB1 PE=1 SV=2
P09486;F5H4E2	37	24	294.1266	5.38E-09	1.52E-07	5.079933	1	2D	0.3PG	35487.6148	SPARC OS=Homo sapiens OX=9606 GN=SPARC PE=1 SV=1
P20908;H7BY82	29	23	243.8753	4.64E-07	1.99E-06	2.164306	1	2D	0.3PG	184244.4298	Collagen alpha-1(V) chain OS=Homo sapiens OX=9606 GN=COL5A1 PE=1 SV=3
P46940;A0A0J9YXZ5;H0YLE8;H0YK	26	21	157.4548	7.70E-06	1.68E-05	2.798056	1	2D	0.3PG	189879.6047	Ras GTPase-activating-like protein IQGAP1 OS=Homo sapiens OX=9606 GN=IQGAP1 PE=1 SV=1
P06733;A0A2R8Y6G6;K7EM90;A0A	27	20	280.7822	4.55E-06	1.09E-05	1.619191	1	HYS	2D	47511.201	Alpha-enolase OS=Homo sapiens OX=9606 GN=ENO1 PE=1 SV=2
O75326;F5GYX3	21	20	199.4528	1.62E-09	7.64E-08	3.464994	1	0.3PG	2D	75907.3852	Semaphorin-7A OS=Homo sapiens OX=9606 GN=SEMA7A PE=1 SV=1
Q02809;Q5JXB7;Q5JXB8	21	20	150.9292	3.68E-06	9.23E-06	2.325337	1	2D	0.3PG	84120.5831	Procollagen-lysine_2-oxoglutarate 5-dioxygenase 1 OS=Homo sapiens OX=9606 GN=PLOD1 PE=1 SV=2
P01871	21	19	212.9037	8.67E-09	1.84E-07	6.039327	1	0.3PG	2D	50124.1273	Immunoglobulin heavy constant mu OS=Homo sapiens OX=9606 GN=IGHM PE=1 SV=4
P07996;A8MZG1	23	19	242.5013	7.69E-08	6.81E-07	2.8035	1	2D	0.3PG	133374.7259	Thrombospondin-1 OS=Homo sapiens OX=9606 GN=THBS1 PE=1 SV=2
P13639	25	19	181.9987	0.000395	0.000512	1.575637	0.99997	HYS	2D	96307.8182	Elongation factor 2 OS=Homo sapiens OX=9606 GN=EEF2 PE=1 SV=4
P01009;G3V2B9;G3V544;A0A0B4J2	38	19	294.9419	1.97E-07	1.25E-06	6.984629	1	0.3PG	2D	46907.7136	Alpha-1-antitrypsin OS=Homo sapiens OX=9606 GN=SERPINA1 PE=1 SV=3
Q7Z406;M0QY43;A0A2R8Y4C3	37	18	216.0067	2.89E-05	5.16E-05	19.54848	1	0.3PG	HYS	228840.3357	Myosin-14 OS=Homo sapiens OX=9606 GN=MYH14 PE=1 SV=2
P18206;A0A096LPE1	30	18	208.6529	0.013278	0.014377	1.238949	0.857642	HYS	2D	124369.8521	Vinculin OS=Homo sapiens OX=9606 GN=VCL PE=1 SV=4
B4E1Z4;E7ETN3;P00751;A0A0G2JH	22	18	191.0333	1.31E-07	9.95E-07	4.124128	1	0.3PG	2D	143281.0401	C3/C5 convertase OS=Homo sapiens OX=9606 PE=1 SV=1
P05997;A0A087WYX9;A0A3B3IRH9	25	17	207.7837	1.56E-05	3.05E-05	2.33046	1	HYS	0.3PG	145879.7858	Collagen alpha-2(V) chain OS=Homo sapiens OX=9606 GN=COL5A2 PE=1 SV=3
O00391;A8MXT8	21	17	215.4251	2.72E-06	7.38E-06	2.862587	1	2D	0.3PG	83376.2172	Sulfhydryl oxidase 1 OS=Homo sapiens OX=9606 GN=QSOX1 PE=1 SV=3
P02790;Q9BS19	21	17	270.8115	1.59E-08	2.59E-07	4.917995	1	0.3PG	2D	52417.7931	Hemopexin OS=Homo sapiens OX=9606 GN=HPX PE=1 SV=2
P21810;C9JKG1	19	17	151.0319	4.92E-10	5.10E-08	16.72983	1	2D	HYS	42053.3216	Biglycan OS=Homo sapiens OX=9606 GN=BGN PE=1 SV=2
P36955;I3L4N7;I3L107;I3L4Z0;I3L2	22	17	200.8357	1.43E-07	1.04E-06	3.945842	1	2D	0.3PG	46483.3447	Pigment epithelium-derived factor OS=Homo sapiens OX=9606 GN=SERPINF1 PE=1 SV=4
P02774;D6RF35;D6RBJ7;D6RF20	17	17	176.0183	7.27E-07	2.68E-06	3.641244	1	0.3PG	2D	54514.372	Vitamin D-binding protein OS=Homo sapiens OX=9606 GN=GC PE=1 SV=2
Q9Y4K0;E5RFY0;E5RHH3;E5RJL2;H	17	16	140.0596	3.61E-08	4.32E-07	5.487738	1	2D	0.3PG	88834.9278	Lysyl oxidase homolog 2 OS=Homo sapiens OX=9606 GN=LOXL2 PE=1 SV=1
Q14766;E7EV71;C9JD84;C9JDW2;H	17	16	132.8189	2.25E-09	9.10E-08	3.769138	1	2D	0.3PG	195236.3596	Latent-transforming growth factor beta-binding protein 1 OS=Homo sapiens OX=9606 GN=LTBP1 PE=1 SV=4
Q14624;B7ZKJ8;H7C0L5	22	16	179.5471	2.85E-07	1.50E-06	3.962607	1	0.3PG	2D	103585.7087	Inter-alpha-trypsin inhibitor heavy chain H4 OS=Homo sapiens OX=9606 GN=ITIH4 PE=1 SV=4
Q16270	16	16	131.9795	2.46E-08	3.42E-07	3.653944	1	2D	0.3PG	30156.8435	Insulin-like growth factor-binding protein 7 OS=Homo sapiens OX=9606 GN=IGFBP7 PE=1 SV=1
P02647;F8W696	22	16	205.4805	2.67E-07	1.44E-06	2.541764	1	0.3PG	2D	30777.8694	Apolipoprotein A-I OS=Homo sapiens OX=9606 GN=APOA1 PE=1 SV=1
P01033;Q5H9A7;H0Y789;Q5H9B5;	19	16	161.0104	5.20E-09	1.52E-07	3.796026	1	2D	0.3PG	23855.2313	Metalloproteinase inhibitor 1 OS=Homo sapiens OX=9606 GN=TIMP1 PE=1 SV=1
A0A0A0MS51;A0A0A0MT01;P0639	21	16	187.706	0.00027	0.000361	1.579429	0.999995	2D	0.3PG	82810.8576	Actin-depolymerizing factor OS=Homo sapiens OX=9606 GN=GSN PE=1 SV=1
P35442;A0A3B3ITK0;A0A7I2V585;	19	15	165.508	2.37E-07	1.38E-06	4.305828	1	2D	0.3PG	133869.2341	Thrombospondin-2 OS=Homo sapiens OX=9606 GN=THBS2 PE=1 SV=2
P11021;A0A7P0TB36;A0A7P0TAI0	25	14	207.4165	6.07E-06	1.38E-05	1.672504	1	HYS	2D	72447.1105	Endoplasmic reticulum chaperone BiP OS=Homo sapiens OX=9606 GN=HSPA5 PE=1 SV=2
P00338;F5GXY2;F5GXH2;F5GYU2;F	18	14	200.1772	0.000359	0.000469	1.468585	0.99998	HYS	0.3PG	36973.9299	L-lactate dehydrogenase A chain OS=Homo sapiens OX=9606 GN=LDHA PE=1 SV=2
B4DNK4;P14618;H3BTN5;H3BR70;	19	14	162.7093	6.35E-05	0.000102	1.563639	1	HYS	0.3PG	50468.1343	Pyruvate kinase OS=Homo sapiens OX=9606 GN=PKM PE=1 SV=1
Q16610	18	14	157.5069	0.003548	0.004081	1.117192	0.978571	HYS	0.3PG	62270.9165	Extracellular matrix protein 1 OS=Homo sapiens OX=9606 GN=ECM1 PE=1 SV=2
P01008	18	14	189.4471	1.91E-06	5.62E-06	3.566274	1	0.3PG	2D	53058.7536	Antithrombin-III OS=Homo sapiens OX=9606 GN=SERPINC1 PE=1 SV=1
O43707;F5GXS2;H7C144;K7EP19	35	14	322.008	0.00054	0.000684	1.231565	0.999887	0.3PG	HYS	105310.4484	Alpha-actinin-4 OS=Homo sapiens OX=9606 GN=ACTN4 PE=1 SV=2
Q08380;K7EP36;K7EKQ5;K7ESM3;	19	14	179.8215	1.19E-06	3.82E-06	2.657097	1	0.3PG	HYS	66243.5215	Galectin-3-binding protein OS=Homo sapiens OX=9606 GN=LGALS3BP PE=1 SV=1
C9JNG9	21	14	191.3497	2.77E-06	7.42E-06	2.845824	1	2D	0.3PG	77287.1816	Collagen alpha-3(VI) chain (Fragment) OS=Homo sapiens OX=9606 GN=COL6A3 PE=1 SV=1
P05155;A0A7I2V2D2;E9PGN7;H9K	17	14	181.1366	0.000209	0.000284	1.478145	0.999999	2D	0.3PG	55382.4019	Plasma protease C1 inhibitor OS=Homo sapiens OX=9606 GN=SERPING1 PE=1 SV=2
P30101	16	14	134.5875	3.90E-06	9.67E-06	2.077118	1	HYS	0.3PG	57181.6493	Protein disulfide-isomerase A3 OS=Homo sapiens OX=9606 GN=PDIA3 PE=1 SV=4
P08572;A0A087WY39;A0A3B3ITQ8	22	14	179.1432	2.39E-07	1.38E-06	1.851688	1	2D	0.3PG	168750.9211	Collagen alpha-2(IV) chain OS=Homo sapiens OX=9606 GN=COL4A2 PE=1 SV=4
Q76M96;H7C5K4;C9J8I6	16	14	137.7435	4.78E-07	2.00E-06	5.123286	1	2D	0.3PG	108573.125	Coiled-coil domain-containing protein 80 OS=Homo sapiens OX=9606 GN=CCDC80 PE=1 SV=1
P26641	15	13	101.5363	2.01E-07	1.27E-06	8.043175	1	HYS	0.3PG	50461.03	Elongation factor 1-gamma OS=Homo sapiens OX=9606 GN=EEF1G PE=1 SV=3
Q01995;H0YCU9;E9PJ32	14	13	121.5314	3.48E-08	4.24E-07	2.551083	1	HYS	0.3PG	22667.9987	Transgelin OS=Homo sapiens OX=9606 GN=TAGLN PE=1 SV=4
P04075;J3KPS3;H3BQN4;H3BR04;H	13	13	126.3314	1.59E-05	3.10E-05	1.619588	1	HYS	2D	39876.2787	Fructose-bisphosphate aldolase A OS=Homo sapiens OX=9606 GN=ALDOA PE=1 SV=2
A0A7P0NMY4;A0A087WWY3;A0A7	30	13	202.4841	2.71E-07	1.44E-06	2.336384	1	0.3PG	2D	169328.5556	Filamin-A OS=Homo sapiens OX=9606 GN=FLNA PE=4 SV=1
P14543	18	13	159.8145	1.12E-05	2.32E-05	2.164005	1	2D	HYS	139228.5047	Nidogen-1 OS=Homo sapiens OX=9606 GN=NID1 PE=1 SV=3
A0A087WY61;Q14980;F5H4J1;F5H	18	12	87.3793	6.37E-07	2.46E-06	3.348549	1	0.3PG	2D	237531.3574	Nuclear mitotic apparatus protein 1 OS=Homo sapiens OX=9606 GN=NUMA1 PE=1 SV=1
A0A7P0T823;A0A7P0TAE1;A0A7P0	19	12	140.3231	2.96E-05	5.26E-05	2.193513	1	2D	0.3PG	93992.618	Endoplasmin OS=Homo sapiens OX=9606 GN=HSP90B1 PE=4 SV=1
P60174;U3KPZ0;U3KQF3;U3KPS5	13	12	126.2899	2.04E-05	3.87E-05	1.623875	1	2D	0.3PG	26954.6554	Triosephosphate isomerase OS=Homo sapiens OX=9606 GN=TPI1 PE=1 SV=4
P35908;P12035	19	12	145.8536	0.000127	0.000184	2.042575	1	0.3PG	2D	65718.1023	Keratin_ type II cytoskeletal 2 epidermal OS=Homo sapiens OX=9606 GN=KRT2 PE=1 SV=2

P07737;K7EJ44;I3L3D5	12	12	117.3906	4.40E-07	1.93E-06	2.16657	1	HYS	0.3PG	15225.3488	Profilin-1 OS=Homo sapiens OX=9606 GN=PFN1 PE=1 SV=2
P01834;A0A5H1ZRQ3	15	12	115.4262	1.14E-07	8.98E-07	7.410698	1	0.3PG	2D	11936.1291	Immunoglobulin kappa constant OS=Homo sapiens OX=9606 GN=IGKC PE=1 SV=2
A0A6Q8PFJ0;P02545;Q3BDU5;Q5T	16	12	113.0611	0.000258	0.000347	2.005315	0.999996	2D	0.3PG	81473.6641	Prelamin-A/C OS=Homo sapiens OX=9606 GN=LMNA PE=1 SV=1
Q9BZ29;A0A0A0MSY4;A0A088AWI	19	11	98.4131	2.20E-06	6.27E-06	1.815765	1	2D	0.3PG	238670.1091	Dedicator of cytokinesis protein 9 OS=Homo sapiens OX=9606 GN=DOCK9 PE=1 SV=2
P55268	13	11	76.9523	5.28E-07	2.13E-06	2.96663	1	0.3PG	HYS	203109.6839	Laminin subunit beta-2 OS=Homo sapiens OX=9606 GN=LAMB2 PE=1 SV=1
P62258;B4DJF2;I3L3T1;K7EM20;K7	16	11	187.3021	2.53E-05	4.62E-05	1.510588	1	HYS	0.3PG	29345.0493	14-3-3 protein epsilon OS=Homo sapiens OX=9606 GN=YWHAE PE=1 SV=1
Q92743;A0A3B3IU24;H0Y7G9	14	11	105.1995	3.40E-05	5.95E-05	1.812504	1	2D	0.3PG	52199.4523	Serine protease HTRA1 OS=Homo sapiens OX=9606 GN=HTRA1 PE=1 SV=1
P03956	13	11	141.1756	1.26E-07	9.84E-07	4.252566	1	HYS	0.3PG	54178.068	Interstitial collagenase OS=Homo sapiens OX=9606 GN=MMP1 PE=1 SV=3
P35527;K7EQQ3	16	11	103.3838	3.59E-07	1.75E-06	1.965085	1	HYS	2D	62292.5365	Keratin_ type I cytoskeletal 9 OS=Homo sapiens OX=9606 GN=KRT9 PE=1 SV=3
P50395;Q5SX87;Q5SX86;Q5SX90;V	18	11	141.6249	3.36E-05	5.88E-05	1.693575	1	HYS	0.3PG	51119.5596	Rab GDP dissociation inhibitor beta OS=Homo sapiens OX=9606 GN=GDI2 PE=1 SV=2
P08238;F8W026;Q58FF8	27	11	205.3921	1.52E-06	4.72E-06	2.709418	1	HYS	0.3PG	83606.5094	Heat shock protein HSP 90-beta OS=Homo sapiens OX=9606 GN=HSP90AB1 PE=1 SV=4
P13797;A0A0A0MSQ0;U3KQI3	26	11	209.779	9.36E-05	0.000141	1.837207	1	HYS	0.3PG	71324.4032	Plastin-3 OS=Homo sapiens OX=9606 GN=PLS3 PE=1 SV=4
P13796;Q5TBN3	22	11	146.9934	1.10E-06	3.67E-06	1.975314	1	0.3PG	2D	70858.8014	Plastin-2 OS=Homo sapiens OX=9606 GN=LCP1 PE=1 SV=6
Q00610;A0A087WVQ6;J3KS13;K7E	20	11	134.1039	0.159416	0.163061	1.135409	0.328904	2D	0.3PG	193382.8096	Clathrin heavy chain 1 OS=Homo sapiens OX=9606 GN=CLTC PE=1 SV=5
H0YND0;A0A6I8PL22	12	11	115.4415	7.67E-09	1.75E-07	2.313661	1	2D	0.3PG	99284.6608	Fibrillin-1 (Fragment) OS=Homo sapiens OX=9606 GN=FBN1 PE=1 SV=1
P02760;S4R471;S4R3Y4	14	11	106.1364	2.63E-07	1.44E-06	2.422228	1	0.3PG	2D	39911.9931	Protein AMBP OS=Homo sapiens OX=9606 GN=AMBP PE=1 SV=1
P01857;A0A0A0MS08;A0A0A0MS0	24	11	247.5759	8.15E-09	1.78E-07	8.940447	1	0.3PG	2D	36619.1863	Immunoglobulin heavy constant gamma 1 OS=Homo sapiens OX=9606 GN=IGHG1 PE=1 SV=1
A0A5F9ZHM4;P07195;A0A3B3IS95	13	10	139.9092	1.14E-05	2.35E-05	1.547445	1	HYS	0.3PG	37579.4984	L-lactate dehydrogenase OS=Homo sapiens OX=9606 GN=LDHB PE=1 SV=1
Q12841;H7C4W4	14	10	92.4946	5.39E-08	5.43E-07	4.282044	1	2D	0.3PG	36126.095	Follistatin-related protein 1 OS=Homo sapiens OX=9606 GN=FSTL1 PE=1 SV=1
P51884	12	10	125.9216	4.43E-06	1.07E-05	2.900111	1	2D	HYS	38771.222	Lumican OS=Homo sapiens OX=9606 GN=LUM PE=1 SV=2
P63261;P60709;I3L4N8;A0A6Q8PF	22	10	219.2906	0.008665	0.009566	1.180479	0.913585	0.3PG	HYS	42135.1246	Actin_ cytoplasmic 2 OS=Homo sapiens OX=9606 GN=ACTG1 PE=1 SV=1
Q96AY3;H0Y827;K7ESG6;K7ELI6;K7	16	10	144.0118	9.75E-07	3.40E-06	3.013968	1	2D	0.3PG	64758.3598	Peptidyl-prolyl cis-trans isomerase FKBP10 OS=Homo sapiens OX=9606 GN=FKBP10 PE=1 SV=1
P11142;E9PKE3;E9PNE6;A8K7Q2;E	26	10	256.969	1.44E-06	4.54E-06	2.476191	1	0.3PG	2D	71126.3096	Heat shock cognate 71 kDa protein OS=Homo sapiens OX=9606 GN=HSPA8 PE=1 SV=1
P29401;A0A0B4J1R6;E9PFF2;F8W8	12	10	109.1197	0.001357	0.001632	1.622045	0.997623	2D	0.3PG	68562.0705	Transketolase OS=Homo sapiens OX=9606 GN=TKT PE=1 SV=3
Q92626;H7C1W1;C9J4I9;H7C300	16	10	109.3127	1.02E-06	3.48E-06	7.546438	1	2D	0.3PG	167898.083	Peroxidasin homolog OS=Homo sapiens OX=9606 GN=PXDN PE=1 SV=2
Q96N67;A0A1B0GUE9	13	10	78.2109	2.21E-08	3.19E-07	4.222721	1	0.3PG	2D	244442.9708	Dedicator of cytokinesis protein 7 OS=Homo sapiens OX=9606 GN=DOCK7 PE=1 SV=4
Q5H9U9;H0Y939	16	10	67.3867	0.023432	0.024959	1.157985	0.75765	HYS	0.3PG	199954.971	Probable ATP-dependent RNA helicase DDX60-like OS=Homo sapiens OX=9606 GN=DDX60L PE=2 SV=2
O43852;H0Y875	12	10	108.2148	6.13E-06	1.39E-05	1.555011	1	HYS	0.3PG	37220.9065	Calumenin OS=Homo sapiens OX=9606 GN=CALU PE=1 SV=2
P00738;A0A0C4DGL8;J3QLC9;J3QR	29	10	267.5293	0.00012	0.000176	2.611151	1	0.3PG	2D	45889.6837	Haptoglobin OS=Homo sapiens OX=9606 GN=HP PE=1 SV=1
P01011;G3V595;G3V3A0;A0A087W	17	10	174.0769	5.43E-08	5.43E-07	3.51582	1	0.3PG	2D	47822.0525	Alpha-1-antichymotrypsin OS=Homo sapiens OX=9606 GN=SERPINA3 PE=1 SV=2
H0YMW4;P07355;H0YL33;H0YM50	12	10	145.9102	2.96E-08	3.87E-07	4.000214	1	HYS	0.3PG	42242.0792	Annexin OS=Homo sapiens OX=9606 GN=ANXA2 PE=1 SV=2
P41219;H7C5W5;F8W835	15	9	117.0222	4.71E-07	2.00E-06	2.816349	1	2D	0.3PG	53765.0009	Peripherin OS=Homo sapiens OX=9606 GN=PRPH PE=1 SV=2
P01861;A0A286YFJ8	19	9	153.4066	2.21E-08	3.19E-07	7.34006	1	0.3PG	2D	36453.8384	Immunoglobulin heavy constant gamma 4 OS=Homo sapiens OX=9606 GN=IGHG4 PE=1 SV=1
Q15113	13	9	144.033	4.55E-06	1.09E-05	2.980755	1	HYS	0.3PG	48827.9501	Procollagen C-endopeptidase enhancer 1 OS=Homo sapiens OX=9606 GN=PCOLCE PE=1 SV=2
Q8IX30	10	9	81.0694	2.51E-07	1.43E-06	5.716534	1	HYS	0.3PG	114472.1249	Signal peptide_ CUB and EGF-like domain-containing protein 3 OS=Homo sapiens OX=9606 GN=SCUBE3 PE=1 SV=1
P10909;H0YC35;E7ERK6;H0YLK8;E7	11	9	84.6482	1.87E-07	1.20E-06	18.97659	1	2D	0.3PG	53064.9683	Clusterin OS=Homo sapiens OX=9606 GN=CLU PE=1 SV=1
A0A0U1RRM4;P26599;A0A7I2V621	10	9	62.0361	5.75E-05	9.39E-05	2.415609	1	HYS	0.3PG	62692.3911	Polypyrimidine tract-binding protein 1 OS=Homo sapiens OX=9606 GN=PTBP1 PE=1 SV=1
P50454;E9PJH8;E9PPV6;E9PR70;E9	11	9	98.1302	7.47E-07	2.74E-06	2.948934	1	HYS	2D	46554.6987	Serpin H1 OS=Homo sapiens OX=9606 GN=SERPINH1 PE=1 SV=2
P43652	11	9	78.1418	2.72E-07	1.44E-06	8.420972	1	0.3PG	HYS	71008.13	Afamin OS=Homo sapiens OX=9606 GN=AFM PE=1 SV=1
P62937;C9J5S7;F8WE65;A0A7I2V5J	12	9	134.7402	3.79E-07	1.83E-06	2.027468	1	HYS	0.3PG	18240.6427	Peptidyl-prolyl cis-trans isomerase A OS=Homo sapiens OX=9606 GN=PPIA PE=1 SV=2
P20742	17	9	122.6569	7.01E-09	1.71E-07	3.96235	1	0.3PG	HYS	165345.8858	Pregnancy zone protein OS=Homo sapiens OX=9606 GN=PZP PE=1 SV=4
P00558	14	9	111.5207	4.49E-07	1.95E-06	1.913679	1	HYS	0.3PG	45013.9805	Phosphoglycerate kinase 1 OS=Homo sapiens OX=9606 GN=PGK1 PE=1 SV=3
P07900;G3V2J8	21	9	131.6298	0.000432	0.000555	1.516123	0.999955	HYS	0.3PG	85059.0572	Heat shock protein HSP 90-alpha OS=Homo sapiens OX=9606 GN=HSP90AA1 PE=1 SV=5
P01859;A0A286YEY4	20	9	181.1902	1.01E-10	1.72E-08	8.786869	1	0.3PG	2D	36527.9778	Immunoglobulin heavy constant gamma 2 OS=Homo sapiens OX=9606 GN=IGHG2 PE=1 SV=2
P25940	16	9	97.3345	1.37E-05	2.76E-05	2.283107	1	HYS	0.3PG	172805.2727	Collagen alpha-3(V) chain OS=Homo sapiens OX=9606 GN=COL5A3 PE=1 SV=3
P09211;A8MX94;A0A087X2E9;A0A	10	9	99.8387	3.15E-06	8.26E-06	1.561611	1	HYS	0.3PG	23583.9696	Glutathione S-transferase P OS=Homo sapiens OX=9606 GN=GSTP1 PE=1 SV=2
Q9BX26;A2A341;A2A340	17	9	87.2788	1.51E-05	2.96E-05	3.377071	1	2D	0.3PG	177349.855	Synaptonemal complex protein 2 OS=Homo sapiens OX=9606 GN=SYCP2 PE=2 SV=2
P22692	12	9	87.6679	3.27E-08	4.08E-07	4.664717	1	2D	0.3PG	29131.5938	Insulin-like growth factor-binding protein 4 OS=Homo sapiens OX=9606 GN=IGFBP4 PE=1 SV=2
P23142;B1AHL2;B1AHM7;B1AHM9	10	9	97.6998	9.16E-08	7.95E-07	3.685082	1	2D	HYS	81320.1098	Fibulin-1 OS=Homo sapiens OX=9606 GN=FBLN1 PE=1 SV=4
P07602;C9JIZ6;A0A0J9YXB8	10	8	73.6304	2.32E-07	1.38E-06	3.997882	1	2D	0.3PG	59937.6442	Prosaposin OS=Homo sapiens OX=9606 GN=PSAP PE=1 SV=2
F5H369;O75747;F5H7Y7;A0A087W	10	8	58.0186	4.55E-05	7.67E-05	1.889301	1	2D	0.3PG	172621.6397	Phosphatidylinositol-4-phosphate 3-kinase OS=Homo sapiens OX=9606 GN=PIK3C2G PE=1 SV=1
Q6EMK4	8	8	59.1937	0.000143	0.000206	3.202598	1	HYS	0.3PG	72796.2461	Vasorin OS=Homo sapiens OX=9606 GN=VASN PE=1 SV=1
P63104;B0AZS6;E7EX29;B7Z2E6;H0	11	8	144.5105	0.000125	0.000183	1.543115	1	HYS	0.3PG	27916.2394	14-3-3 protein zeta/delta OS=Homo sapiens OX=9606 GN=YWHAZ PE=1 SV=1
O14786;E9PEP6;E7EX60;Q5JWQ4;	11	8	70.1755	0.000506	0.000644	1.741159	0.999913	2D	0.3PG	104389.1104	Neuropilin-1 OS=Homo sapiens OX=9606 GN=NRP1 PE=1 SV=3
Q02818;H7BZI1;C9JKZ2;C9J3C1;C9J	12	8	103.4645	1.07E-07	8.68E-07	3.323563	1	2D	0.3PG	53879.3872	Nucleobindin-1 OS=Homo sapiens OX=9606 GN=NUCB1 PE=1 SV=4
P16035;B4DFW2;K7EIX4;K7EL90	11	8	121.7864	5.51E-07	2.20E-06	4.408652	1	2D	0.3PG	25083.634	Metalloproteinase inhibitor 2 OS=Homo sapiens OX=9606 GN=TIMP2 PE=1 SV=2
Q13201;E7EPG1	11	8	75.6707	4.66E-07	1.99E-06	3.48741	1	2D	0.3PG	139307.9588	Multimerin-1 OS=Homo sapiens OX=9606 GN=MMRN1 PE=1 SV=3
P02462;A0A3B3ISV3	11	8	67.9411	1.13E-06	3.76E-06	2.782956	1	2D	0.3PG	161751.5969	Collagen alpha-1(IV) chain OS=Homo sapiens OX=9606 GN=COL4A1 PE=1 SV=4
Q2M2I5	13	8	80.4458	0.006006	0.00678	1.24499	0.948083	2D	0.3PG	55600.7377	Keratin_ type I cytoskeletal 24 OS=Homo sapiens OX=9606 GN=KRT24 PE=1 SV=1
A0A0A0MSI0;Q06830;A0A0A0MRC	12	8	86.6583	0.009429	0.010354	1.280947	0.903892	2D	0.3PG	19146.8635	Peroxiredoxin-1 (Fragment) OS=Homo sapiens OX=9606 GN=PRDX1 PE=1 SV=1
Q14019;H3BT58	8	8	71.9581	5.23E-07	2.13E-06	2.448342	1	HYS	0.3PG	16059.0549	Coactosin-like protein OS=Homo sapiens OX=9606 GN=COTL1 PE=1 SV=3
P01876;A0A286YEY1	15	8	140.9114	6.21E-10	5.10E-08	7.938981	1	0.3PG	2D	38510.0827	Immunoglobulin heavy constant alpha 1 OS=Homo sapiens OX=9606 GN=IGHA1 PE=1 SV=2
P05787	16	8	102.1615	7.95E-07	2.84E-06	2.234396	1	2D	0.3PG	53704.3638	Keratin_ type II cytoskeletal 8 OS=Homo sapiens OX=9606 GN=KRT8 PE=1 SV=7
P35580;E7ERA5	19	8	108.7289	4.15E-07	1.86E-06	4.220211	1	0.3PG	2D	229968.9231	Myosin-10 OS=Homo sapiens OX=9606 GN=MYH10 PE=1 SV=3
E7EPB6;P13569;A0A3B3ITW5;A0A3	15	8	87.9462	6.05E-06	1.38E-05	3.380607	1	HYS	0.3PG	164381.0047	Cystic fibrosis transmembrane conductance regulator (Fragment) OS=Homo sapiens OX=9606 GN=CFTR PE=1 SV=1
P19827;H7C0N0	16	8	136.5379	4.25E-05	7.25E-05	2.038129	1	0.3PG	2D	101845.5159	Inter-alpha-trypsin inhibitor heavy chain H1 OS=Homo sapiens OX=9606 GN=ITIH1 PE=1 SV=3
E9PGC5;Q15262;Q5TG12;H0YDP9	10	7	57.7724	0.000539	0.000684	2.203433	0.999888	HYS	0.3PG	168024.9148	Protein-tyrosine-phosphatase OS=Homo sapiens OX=9606 GN=PTPRK PE=1 SV=1
Q9Y678;H0Y8X7	11	7	65.251	0.093675	0.096397	1.190987	0.445628	2D	0.3PG	99030.073	Coatomer subunit gamma-1 OS=Homo sapiens OX=9606 GN=COPG1 PE=1 SV=1
P02765;C9JV77	10	7	81.9314	2.14E-07	1.32E-06	6.971146	1	0.3PG	2D	40139.1565	Alpha-2-HS-glycoprotein OS=Homo sapiens OX=9606 GN=AHSG PE=1 SV=2
P04406;E7EUT5	12	7	98.211	0.001491	0.001775	1.416002	0.996934	HYS	0.3PG	36224.3681	Glyceraldehyde-3-phosphate dehydrogenase OS=Homo sapiens OX=9606 GN=GAPDH PE=1 SV=3
P15291;Q86XA6;O43286	7	7	51.374	1.81E-06	5.40E-06	2.915158	1	2D	0.3PG	44319.5415	Beta-1_4-galactosyltransferase 1 OS=Homo sapiens OX=9606 GN=B4GALT1 PE=1 SV=5
P07196	10	7	79.8407	6.18E-06	1.39E-05	2.855069	1	HYS	2D	61573.714	Neurofilament light polypeptide OS=Homo sapiens OX=9606 GN=NEFL PE=1 SV=3

P08476	8	7	66.3236	6.45E-09	1.67E-07	4.474654	1	HYS	0.3PG	48240.738	Inhibin beta A chain OS=Homo sapiens OX=9606 GN=INHBA PE=1 SV=2
O60687;A0A1W2PR88;A0A1W2PRE	9	7	70.5446	2.98E-07	1.54E-06	3.668824	1	2D	HYS	54226.5447	Sushi repeat-containing protein SRPX2 OS=Homo sapiens OX=9606 GN=SRPX2 PE=1 SV=1
A0A2U3TZU2;P06744;K7EQ48;A0A	9	7	88.9766	0.000667	0.000835	1.605464	0.999751	HYS	0.3PG	67571.2151	Glucose-6-phosphate isomerase OS=Homo sapiens OX=9606 GN=GPI PE=1 SV=1
P54289;H0Y715	8	7	41.5215	2.19E-06	6.26E-06	2.300005	1	0.3PG	2D	125708.7376	Voltage-dependent calcium channel subunit alpha-2/delta-1 OS=Homo sapiens OX=9606 GN=CACNA2D1 PE=1 SV=3
Q149M9;F8W0U9	11	7	47.8706	8.30E-10	5.10E-08	72.11919	1	HYS	2D	176947.085	NACHT domain- and WD repeat-containing protein 1 OS=Homo sapiens OX=9606 GN=NWD1 PE=1 SV=3
F2Z3N3;Q68BL8;H0YE85;H0YEW8	8	7	81.784	1.01E-07	8.43E-07	3.29613	1	2D	0.3PG	84811.477	Olfactomedin-like protein 2B OS=Homo sapiens OX=9606 GN=OLFML2B PE=1 SV=1
P15121;E9PCX2;E9PEF9;Q5T621;V9	9	7	77.9707	0.037068	0.038946	1.610154	0.660216	HYS	0.3PG	36252.6413	Aldo-keto reductase family 1 member B1 OS=Homo sapiens OX=9606 GN=AKR1B1 PE=1 SV=3
K7EL50;P27797;A0A7P0T861;K7EJE	11	7	109.208	5.83E-05	9.50E-05	1.736695	1	HYS	0.3PG	43585.2062	Calreticulin OS=Homo sapiens OX=9606 GN=CALR PE=1 SV=2
E9PF17;Q86W61	12	7	96.4596	2.63E-07	1.44E-06	3.435777	1	0.3PG	HYS	178598.1199	Versican core protein OS=Homo sapiens OX=9606 GN=VCAN PE=1 SV=2
Q96JN2	12	7	61.8936	4.24E-05	7.25E-05	1.486953	1	2D	0.3PG	135699.3462	Coiled-coil domain-containing protein 136 OS=Homo sapiens OX=9606 GN=CCDC136 PE=1 SV=3
Q12931;I3L0K7;I3L239;I3L253	12	7	76.5123	2.34E-05	4.36E-05	2.336132	1	HYS	0.3PG	80395.2776	Heat shock protein 75 kDa_ mitochondrial OS=Homo sapiens OX=9606 GN=TRAP1 PE=1 SV=3
P09382	10	7	98.1347	4.43E-07	1.93E-06	1.928066	1	2D	0.3PG	15057.8861	Galectin-1 OS=Homo sapiens OX=9606 GN=LGALS1 PE=1 SV=2
P53675;A0A087WXH4;A0A087WV7	14	7	80.9537	2.76E-08	3.67E-07	3.031392	1	HYS	0.3PG	189140.241	Clathrin heavy chain 2 OS=Homo sapiens OX=9606 GN=CLTCL1 PE=1 SV=2
Q9UKX2	13	7	73.4889	1.40E-09	7.01E-08	6.165664	1	0.3PG	2D	224071.3252	Myosin-2 OS=Homo sapiens OX=9606 GN=MYH2 PE=1 SV=1
P07585;F8VUF6;F8VWU0;F8VXZ8;F	8	7	79.9795	1.43E-07	1.04E-06	2.799425	1	2D	HYS	40088.9951	Decorin OS=Homo sapiens OX=9606 GN=DCN PE=1 SV=1
O94822;H7BYG8;S4R3T2	14	7	90.3391	2.45E-06	6.82E-06	3.129267	1	HYS	0.3PG	203232.596	E3 ubiquitin-protein ligase listerin OS=Homo sapiens OX=9606 GN=LTN1 PE=1 SV=6
P31150;G5E9U5	15	7	109.4868	0.005292	0.00599	1.551236	0.957287	HYS	0.3PG	51210.1274	Rab GDP dissociation inhibitor alpha OS=Homo sapiens OX=9606 GN=GDI1 PE=1 SV=2
P08603;A0A0D9SG88;Q5TFM2	10	7	47.9671	0.001052	0.001276	1.216657	0.998864	HYS	2D	143772.7447	Complement factor H OS=Homo sapiens OX=9606 GN=CFH PE=1 SV=4
P19652	15	7	152.0958	1.12E-05	2.32E-05	2.332154	1	0.3PG	2D	23887.7903	Alpha-1-acid glycoprotein 2 OS=Homo sapiens OX=9606 GN=ORM2 PE=1 SV=2
P18669;P15259	13	7	128.9541	9.06E-08	7.94E-07	2.262592	1	HYS	0.3PG	28918.0363	Phosphoglycerate mutase 1 OS=Homo sapiens OX=9606 GN=PGAM1 PE=1 SV=2
Q9Y6D6;E5RIF2;E5RJN9	17	7	97.815	2.48E-06	6.87E-06	3.479375	1	2D	0.3PG	210991.5118	Brefeldin A-inhibited guanine nucleotide-exchange protein 1 OS=Homo sapiens OX=9606 GN=ARFGEF1 PE=1 SV=2
Q9HBG6;D6RIB5;H0Y9Q2	9	7	51.4729	6.43E-05	0.000103	1.776129	1	2D	0.3PG	143878.4173	Intraflagellar transport protein 122 homolog OS=Homo sapiens OX=9606 GN=IFT122 PE=1 SV=2
P17655	10	7	62.5984	3.49E-08	4.24E-07	4.211036	1	HYS	0.3PG	80850.8697	Calpain-2 catalytic subunit OS=Homo sapiens OX=9606 GN=CAPN2 PE=1 SV=6
A0A7P0Z497;P23284;A0A7P0TB45;	9	7	81.6078	5.28E-07	2.13E-06	2.151264	1	HYS	0.3PG	20267.2898	Peptidyl-prolyl cis-trans isomerase B OS=Homo sapiens OX=9606 GN=PPIB PE=4 SV=1
A0A0G2JIW1;P0DMV8;P0DMV9;V9	17	7	127.9943	1.79E-05	3.46E-05	2.069091	1	2D	0.3PG	70394.5072	Heat shock 70 kDa protein 1B OS=Homo sapiens OX=9606 GN=HSPA1B PE=1 SV=1
H0Y8C6;O00410;A0A096LPA6;E7EC	9	7	54.427	0.000154	0.000218	1.977746	1	2D	0.3PG	125288.2974	Importin-5 (Fragment) OS=Homo sapiens OX=9606 GN=IPO5 PE=1 SV=1
Q01518;Q5T0R1;Q5T0R2;Q5T0R3;(	10	7	66.7442	2.57E-05	4.67E-05	2.278947	1	HYS	0.3PG	52357.7623	Adenylyl cyclase-associated protein 1 OS=Homo sapiens OX=9606 GN=CAP1 PE=1 SV=5
P53804;E9PCE7;E9PMP8	16	7	94.2207	0.248382	0.251638	1.211163	0.242106	0.3PG	2D	233005.7662	E3 ubiquitin-protein ligase TTC3 OS=Homo sapiens OX=9606 GN=TTC3 PE=1 SV=2
Q9ULV4;B4E3S0;H0YHL7;F8VRE9;F	8	7	59.9136	1.22E-06	3.90E-06	3.591316	1	0.3PG	2D	53933.4684	Coronin-1C OS=Homo sapiens OX=9606 GN=CORO1C PE=1 SV=1
A0A087X0K0;P39059	6	6	44.5363	2.36E-05	4.38E-05	2.066356	1	2D	HYS	140515.697	Collagen alpha-1(XV) chain OS=Homo sapiens OX=9606 GN=COL15A1 PE=1 SV=1
E9PMV1;E9PIA2	8	6	57.2138	0.480575	0.482278	1.122529	0.135561	2D	HYS	81008.571	Plectin (Fragment) OS=Homo sapiens OX=9606 GN=PLEC PE=1 SV=1
P04004;F5GX75;H0YJW9	8	6	62.6027	7.12E-05	0.000112	1.942489	1	2D	HYS	55104.0374	Vitronectin OS=Homo sapiens OX=9606 GN=VTN PE=1 SV=1
P19013	10	6	91.9366	5.32E-06	1.24E-05	2.54145	1	2D	0.3PG	56543.3077	Keratin_ type II cytoskeletal 4 OS=Homo sapiens OX=9606 GN=KRT4 PE=1 SV=5
P40925;A0A5K1VW95;B9A041;B8Z	10	6	71.3525	1.60E-07	1.11E-06	3.91639	1	0.3PG	HYS	36654.3026	Malate dehydrogenase_ cytoplasmic OS=Homo sapiens OX=9606 GN=MDH1 PE=1 SV=4
H0Y4P7	8	6	53.2938	0.000341	0.000446	2.15551	0.999984	2D	HYS	70854.5329	Collagen alpha-1(XII) chain (Fragment) OS=Homo sapiens OX=9606 GN=COL12A1 PE=1 SV=1
P61158;B4DXW1;F8WDR7;A0A0A0	8	6	56.6114	0.000592	0.000744	1.978268	0.99984	HYS	0.3PG	47827.4696	Actin-related protein 3 OS=Homo sapiens OX=9606 GN=ACTR3 PE=1 SV=3
Q9NZ71;D6RA96;A0A0C4DGC7;X6R	10	6	52.1452	0.026661	0.028257	1.135682	0.73145	2D	0.3PG	135051.6451	Regulator of telomere elongation helicase 1 OS=Homo sapiens OX=9606 GN=RTEL1 PE=1 SV=2
H0Y599;Q8N1I0;F5GXW1	10	6	58.0099	3.58E-07	1.75E-06	3.811467	1	2D	0.3PG	230405.2325	Dedicator of cytokinesis protein 4 (Fragment) OS=Homo sapiens OX=9606 GN=DOCK4 PE=1 SV=1
A0A7I2V2U7;A0A7I2V349;A0A7I2V	10	6	64.9715	0.001636	0.001942	1.264284	0.996096	HYS	0.3PG	68649.8201	Nucleolin OS=Homo sapiens OX=9606 GN=NCL PE=1 SV=1
P30041	8	6	66.0732	4.80E-05	8.07E-05	1.627441	1	2D	0.3PG	25149.0747	Peroxiredoxin-6 OS=Homo sapiens OX=9606 GN=PRDX6 PE=1 SV=3
P62987;B4DV12;F5GXK7;F5GYU3;F	7	6	57.5293	1.33E-07	1.00E-06	2.756177	1	HYS	0.3PG	15013.472	Ubiquitin-60S ribosomal protein L40 OS=Homo sapiens OX=9606 GN=UBA52 PE=1 SV=2
Q9Y6C2;A0A0A0MT20	7	6	44.9874	2.41E-05	4.43E-05	2.420703	1	2D	0.3PG	108006.8573	EMILIN-1 OS=Homo sapiens OX=9606 GN=EMILIN1 PE=1 SV=3
P55058	10	6	95.6493	0.000102	0.000153	2.387331	1	2D	0.3PG	54967.6638	Phospholipid transfer protein OS=Homo sapiens OX=9606 GN=PLTP PE=1 SV=1
P19823;Q5T985;Q5T987	7	6	54.355	2.75E-06	7.40E-06	2.490136	1	0.3PG	2D	106919.8497	Inter-alpha-trypsin inhibitor heavy chain H2 OS=Homo sapiens OX=9606 GN=ITIH2 PE=1 SV=2
P02763	13	6	124.9262	8.93E-10	5.10E-08	3.212633	1	0.3PG	2D	23739.6925	Alpha-1-acid glycoprotein 1 OS=Homo sapiens OX=9606 GN=ORM1 PE=1 SV=1
Q9Y240;V9H0X6;M0R081	6	6	42.4437	2.74E-05	4.93E-05	2.428052	1	HYS	0.3PG	36036.8385	C-type lectin domain family 11 member A OS=Homo sapiens OX=9606 GN=CLEC11A PE=1 SV=1
A0A7I2V346;A0A7I2V4Z0;A0A7I2Y(	8	6	48.6138	4.16E-09	1.01E-05	3.436419	1	2D	0.3PG	58876.104	Heterogeneous nuclear ribonucleoprotein Q OS=Homo sapiens OX=9606 GN=SYNCRIP PE=1 SV=1
A0A087X253;P63010;A0A087WU93	10	6	58.9316	0.000104	0.000154	1.490158	1	0.3PG	2D	102017.0822	AP complex subunit beta OS=Homo sapiens OX=9606 GN=AP2B1 PE=1 SV=1
P31946;A0A0J9YWE8;A0A0J9YWZ2	11	6	118.6905	0.00483	0.005496	1.764351	0.963154	0.3PG	2D	28196.5102	14-3-3 protein beta/alpha OS=Homo sapiens OX=9606 GN=YWHAB PE=1 SV=3
P01860;A0A286YES1;A0A4W9A917	16	6	183.4934	4.16E-09	1.31E-07	8.913081	1	0.3PG	2D	42313.4888	Immunoglobulin heavy constant gamma 3 OS=Homo sapiens OX=9606 GN=IGHG3 PE=1 SV=2
P06703;R4GN98	6	6	78.5312	0.00113	0.001366	2.056209	0.998592	2D	HYS	10236.7832	Protein S100-A6 OS=Homo sapiens OX=9606 GN=S100A6 PE=1 SV=1
Q08043;A0A087WSZ2	17	6	134.9682	0.000143	0.000206	1.820662	1	HYS	2D	103982.858	Alpha-actinin-3 OS=Homo sapiens OX=9606 GN=ACTN3 PE=1 SV=2
Q9NRN5;B4DNG0	11	6	85.6843	0.000335	0.000439	1.722528	0.999986	2D	0.3PG	46409.5117	Olfactomedin-like protein 3 OS=Homo sapiens OX=9606 GN=OLFML3 PE=2 SV=1
A1L4H1;M0QZ17	9	6	64.6902	0.105752	0.108562	1.12639	0.41808	0.3PG	2D	168195.1132	Soluble scavenger receptor cysteine-rich domain-containing protein SSC5D OS=Homo sapiens OX=9606 GN=SSC5D PE=1 SV=3
E7EQR4;P15311	17	6	110.4276	3.49E-05	6.09E-05	1.853651	1	2D	0.3PG	69485.8854	Ezrin OS=Homo sapiens OX=9606 GN=EZR PE=1 SV=3
Q14697;F5H6X6;E9PKU7	9	6	58.3797	0.007758	0.008632	1.522982	0.925273	2D	0.3PG	107330.3755	Neutral alpha-glucosidase AB OS=Homo sapiens OX=9606 GN=GANAB PE=1 SV=3
P22626;A0A7I2V323;A0A7I2V3P1;A	6	6	51.8992	2.54E-07	1.44E-06	2.114683	1	HYS	0.3PG	37486.7876	Heterogeneous nuclear ribonucleoproteins A2/B1 OS=Homo sapiens OX=9606 GN=HNRNPA2B1 PE=1 SV=2
P37802;X6RJP6	9	6	94.1574	5.65E-05	9.29E-05	2.093127	1	HYS	0.3PG	22562.5876	Transgelin-2 OS=Homo sapiens OX=9606 GN=TAGLN2 PE=1 SV=3
Q6YHK3	9	6	49.8739	0.003157	0.003651	1.674664	0.982866	2D	0.3PG	162601.7488	CD109 antigen OS=Homo sapiens OX=9606 GN=CD109 PE=1 SV=2
Q96PD5;M0R2W8	5	5	29.0355	0.23766	0.241063	1.196364	0.250226	0.3PG	2D	62787.3718	N-acetylmuramoyl-L-alanine amidase OS=Homo sapiens OX=9606 GN=PGLYRP2 PE=1 SV=1
P10599	8	5	70.8433	1.03E-06	3.50E-06	1.749824	1	HYS	0.3PG	12022.6557	Thioredoxin OS=Homo sapiens OX=9606 GN=TXN PE=1 SV=3
Q9H7Z3	5	5	26.3203	0.000682	0.000846	1.493092	0.99973	0.3PG	2D	133927.6951	Nuclear exosome regulator NRDE2 OS=Homo sapiens OX=9606 GN=NRDE2 PE=1 SV=3
P0CF74;A0A5H1ZRQ7;A0M8Q6	8	5	45.565	4.97E-06	1.16E-05	4.269081	1	0.3PG	2D	11447.6279	Immunoglobulin lambda constant 6 OS=Homo sapiens OX=9606 GN=IGLC6 PE=1 SV=1
P01034	8	5	67.2239	7.70E-07	2.77E-06	3.319039	1	2D	0.3PG	16027.3632	Cystatin-C OS=Homo sapiens OX=9606 GN=CST3 PE=1 SV=1
E7EWC2;F5H7S7;Q13576;D6R939	5	5	27.209	0.000189	0.000261	1.598317	0.999999	2D	0.3PG	160847.8517	Ras GTPase-activating-like protein IQGAP2 (Fragment) OS=Homo sapiens OX=9606 GN=IQGAP2 PE=1 SV=1
Q92878;A0A494BZX5;A0A494C122	7	5	28.2475	0.033566	0.03531	1.500135	0.682232	2D	0.3PG	154918.9456	DNA repair protein RAD50 OS=Homo sapiens OX=9606 GN=RAD50 PE=1 SV=1
Q32P51;A0A7I2V2L6;A0A7I2V3R8;A	7	5	68.9123	4.91E-07	2.04E-06	2.629733	1	HYS	0.3PG	34396.4687	Heterogeneous nuclear ribonucleoprotein A1-like 2 OS=Homo sapiens OX=9606 GN=HNRNPA1L2 PE=2 SV=2
Q8NDI1;B5MC86	5	5	37.5562	1.29E-07	9.89E-07	4.806276	1	0.3PG	2D	140644.7901	EH domain-binding protein 1 OS=Homo sapiens OX=9606 GN=EHBP1 PE=1 SV=3
P24592;F8VYK9;A0A3B3IUE0;F8VV.	6	5	55.9991	6.58E-07	2.50E-06	2.863476	1	2D	0.3PG	26234.9016	Insulin-like growth factor-binding protein 6 OS=Homo sapiens OX=9606 GN=IGFBP6 PE=1 SV=1
J3QQJ5;Q9Y2L5	7	5	31.9353	4.14E-06	1.01E-05	2.936928	1	2D	0.3PG	156467.2181	Trafficking protein particle complex subunit 8 OS=Homo sapiens OX=9606 GN=TRAPPC8 PE=1 SV=1
Q99538;G3V4E4;G3V2T4;H0YJN9	6	5	69.0233	2.92E-06	7.76E-06	3.849951	1	2D	0.3PG	49810.4742	Legumain OS=Homo sapiens OX=9606 GN=LGMN PE=1 SV=1
Q9BXL7;H7BY05	6	5	30.3683	3.15E-07	1.61E-06	2.275415	1	2D	0.3PG	134595.8338	Caspase recruitment domain-containing protein 11 OS=Homo sapiens OX=9606 GN=CARD11 PE=1 SV=3

Q9HC10	6	5	36.2639	4.29E-06	1.04E-05	2.484157	1	2D	0.3PG	228350.1952	Otoferlin OS=Homo sapiens OX=9606 GN=OTOF PE=2 SV=3
A0A0A0MQX7;Q08629;D6RB21	7	5	43.7491	7.10E-09	1.71E-07	6.523525	1	2D	HYS	43588.2842	Testican-1 OS=Homo sapiens OX=9606 GN=SPOCK1 PE=1 SV=1
A0A7P0T7Z2;A0A7P0T9L9;A0A7P0T	7	5	38.0125	0.00907	0.009986	1.294099	0.908433	HYS	0.3PG	204418.7507	Brefeldin A-inhibited guanine nucleotide-exchange protein 2 OS=Homo sapiens OX=9606 GN=ARFGEF2 PE=4 SV=1
Q9UKU9	6	5	32.3463	1.79E-06	5.37E-06	5.786357	1	2D	0.3PG	57617.7894	Angiopoietin-related protein 2 OS=Homo sapiens OX=9606 GN=ANGPTL2 PE=1 SV=1
O94985;Q5SR54;A0A3B3ITY6	6	5	45.3191	0.000149	0.000211	1.797162	1	2D	0.3PG	111047.4981	Calsyntenin-1 OS=Homo sapiens OX=9606 GN=CLSTN1 PE=1 SV=1
F6SYF8;Q9UBP4;E9PHY3;E9PKK9;E9	6	5	40.3167	1.49E-06	4.66E-06	2.2626	1	2D	0.3PG	41147.7724	Dickkopf-related protein 3 OS=Homo sapiens OX=9606 GN=DKK3 PE=1 SV=1
A0A0A0MTS5;A0A0A6YYF2;K7EIP4;	7	5	39.4967	1.00E-06	3.44E-06	2.802946	1	0.3PG	HYS	186476.7314	HCG1811249_ isoform CRA_f OS=Homo sapiens OX=9606 GN=LAMA3 PE=1 SV=1
P24593;C9JXX4	6	5	40.6328	6.27E-08	5.92E-07	2.721428	1	HYS	0.3PG	31596.8053	Insulin-like growth factor-binding protein 5 OS=Homo sapiens OX=9606 GN=IGFBP5 PE=1 SV=1
P02749;J3QLI0;J3QRN2;J3KS17	6	5	46.8805	9.38E-06	1.99E-05	2.597985	1	0.3PG	2D	39609.831	Beta-2-glycoprotein 1 OS=Homo sapiens OX=9606 GN=APOH PE=1 SV=3
P33908	6	5	48.2517	0.088023	0.0908	1.258998	0.45992	2D	HYS	73196.7737	Mannosyl-oligosaccharide 1_2-alpha-mannosidase IA OS=Homo sapiens OX=9606 GN=MAN1A1 PE=1 SV=3
F8VUX9;Q96KV7;G3V201	7	5	47.1256	8.42E-06	1.83E-05	1.692259	1	0.3PG	2D	191045.0718	WD repeat-containing protein 90 OS=Homo sapiens OX=9606 GN=WDR90 PE=1 SV=1
H7C410;P35052;H7BZL4;H7C024	6	5	42.3775	3.95E-07	1.86E-06	5.791642	1	2D	0.3PG	54705.8336	Glypican-1 OS=Homo sapiens OX=9606 GN=GPC1 PE=1 SV=2
A0A7P0T8D1;A0A7P0TA52;A0A7P0	6	5	57.774	5.24E-07	2.13E-06	5.478823	1	0.3PG	2D	52355.1623	Angiotensinogen OS=Homo sapiens OX=9606 GN=AGT PE=4 SV=1
A0A087X0M4;Q9BWU0;H7C256	7	5	36.6602	3.41E-09	1.17E-07	22.43008	1	0.3PG	HYS	83631.9346	Kanadaptin OS=Homo sapiens OX=9606 GN=SLC4A1AP PE=1 SV=1
P63241;I3L397;I3L504;Q6IS14;C9J4	7	5	81.0816	1.51E-06	4.69E-06	3.830632	1	HYS	0.3PG	17060.4028	Eukaryotic translation initiation factor 5A-1 OS=Homo sapiens OX=9606 GN=EIF5A PE=1 SV=2
P53396	7	5	45.185	0.190314	0.193965	15.53399	0.292886	0.3PG	2D	121751.9198	ATP-citrate synthase OS=Homo sapiens OX=9606 GN=ACLY PE=1 SV=3
C9JEV0;P25311;H7BZJ8	8	5	54.368	0.000147	0.00021	2.208591	1	0.3PG	HYS	26524.9174	Zinc-alpha-2-glycoprotein OS=Homo sapiens OX=9606 GN=AZGP1 PE=1 SV=1
P52565;J3KRE2;J3KTF8;J3QQX2;J3K	8	5	57.2024	2.59E-06	7.11E-06	1.857709	1	HYS	0.3PG	23264.1679	Rho GDP-dissociation inhibitor 1 OS=Homo sapiens OX=9606 GN=ARHGDIA PE=1 SV=3
Q86Y38	6	5	39.9889	9.88E-08	8.32E-07	7.202841	1	2D	0.3PG	108424.8035	Xylosyltransferase 1 OS=Homo sapiens OX=9606 GN=XYLT1 PE=1 SV=1
P31949	6	5	62.1269	0.017933	0.019295	1.29706	0.808097	HYS	0.3PG	11854.5344	Protein S100-A11 OS=Homo sapiens OX=9606 GN=S100A11 PE=1 SV=2
P05546	8	5	52.4466	1.46E-05	2.89E-05	10.37683	1	0.3PG	2D	57241.8282	Heparin cofactor 2 OS=Homo sapiens OX=9606 GN=SERPIND1 PE=1 SV=3
Q9Y696;A6PVS0;O15247	8	5	51.1228	3.69E-05	6.40E-05	1.749313	1	HYS	0.3PG	29000.2696	Chloride intracellular channel protein 4 OS=Homo sapiens OX=9606 GN=CLIC4 PE=1 SV=4
P61981	9	5	116.1068	2.25E-05	4.23E-05	2.269757	1	HYS	0.3PG	28473.7251	14-3-3 protein gamma OS=Homo sapiens OX=9606 GN=YWHAG PE=1 SV=2
Q58FF6	10	5	58.926	4.12E-07	1.86E-06	2.287771	1	2D	0.3PG	58891.778	Putative heat shock protein HSP 90-beta 4 OS=Homo sapiens OX=9606 GN=HSP90AB4P PE=5 SV=1
A0JNW5;A0A0C4DGH6	9	5	36.1424	2.83E-06	7.56E-06	2.074079	1	HYS	0.3PG	165624.4695	UHRF1-binding protein 1-like OS=Homo sapiens OX=9606 GN=UHRF1BP1L PE=1 SV=2
Q6P1J6;H7BYX7	11	5	58.4805	2.67E-05	4.82E-05	3.091396	1	2D	0.3PG	164962.8029	Phospholipase B1_ membrane-associated OS=Homo sapiens OX=9606 GN=PLB1 PE=1 SV=3
O75970;F5H1U9;B7Z824;H0YGQ3;I	9	5	49.5546	1.05E-08	2.03E-07	8.25553	1	HYS	2D	222929.7554	Multiple PDZ domain protein OS=Homo sapiens OX=9606 GN=MPDZ PE=1 SV=2
B9A064;A0A0B4J231;A0A5H1ZRQ4	10	5	97.5407	1.69E-08	2.66E-07	4.054856	1	0.3PG	2D	23405.4375	Immunoglobulin lambda-like polypeptide 5 OS=Homo sapiens OX=9606 GN=IGLL5 PE=2 SV=2
P68032;P62736;P63267;P68133;A6	14	5	142.11	4.75E-07	2.00E-06	2.397252	1	2D	0.3PG	42361.2493	Actin_ alpha cardiac muscle 1 OS=Homo sapiens OX=9606 GN=ACTC1 PE=1 SV=1
P26038;V9GZ54;A0A2R8Y5P0	19	5	148.693	2.97E-07	1.54E-06	4.550828	1	2D	0.3PG	67934.2403	Moesin OS=Homo sapiens OX=9606 GN=MSN PE=1 SV=3
O00469;E7ETU9	10	5	67.9203	0.014542	0.015726	1.306174	0.843504	HYS	0.3PG	85427.0964	Procollagen-lysine_2-oxoglutarate 5-dioxygenase 2 OS=Homo sapiens OX=9606 GN=PLOD2 PE=1 SV=2
O95425;A0A6I8PIX7;A0A0J9YWY6	9	5	48.3463	1.81E-05	3.48E-05	2.117109	1	2D	0.3PG	249571.5833	Supervillin OS=Homo sapiens OX=9606 GN=SVIL PE=1 SV=2
G3V2A4;H0YIY9	7	4	45.5093	2.14E-07	1.32E-06	4.139312	1	0.3PG	2D	210334.9991	Telomerase protein component 1 OS=Homo sapiens OX=9606 GN=TEP1 PE=1 SV=1
Q9H9Y6	7	4	32.7502	2.37E-05	4.38E-05	4.371667	1	2D	0.3PG	129883.551	DNA-directed RNA polymerase I subunit RPA2 OS=Homo sapiens OX=9606 GN=POLR1B PE=1 SV=2
C9IYI1;E7EMS7;C9JCN1;M0R265	4	4	24.5922	2.08E-09	8.82E-08	9.549134	1	2D	HYS	26871.3504	Dermokine OS=Homo sapiens OX=9606 GN=DMKN PE=1 SV=1
P04196	6	4	47.6985	0.000162	0.000227	1.797571	1	0.3PG	HYS	60547.8329	Histidine-rich glycoprotein OS=Homo sapiens OX=9606 GN=HRG PE=1 SV=1
Q15404	5	4	38.2884	1.06E-06	3.55E-06	4.859869	1	HYS	0.3PG	31540.3463	Ras suppressor protein 1 OS=Homo sapiens OX=9606 GN=RSU1 PE=1 SV=3
Q9BQI5	4	4	22.5084	5.20E-08	5.43E-07	3.27164	1	HYS	0.3PG	89507.8946	SH3-containing GRB2-like protein 3-interacting protein 1 OS=Homo sapiens OX=9606 GN=SGIP1 PE=1 SV=2
A0A3B3IS43;H0Y8A5;Q8IV35	4	4	25.5824	1.55E-06	4.77E-06	3.011631	1	2D	0.3PG	119258.6863	WD repeat-containing protein 49 OS=Homo sapiens OX=9606 GN=WDR49 PE=1 SV=1
P17066;P48741	10	4	70.5394	1.20E-08	2.13E-07	24.47971	1	HYS	0.3PG	71484.4509	Heat shock 70 kDa protein 6 OS=Homo sapiens OX=9606 GN=HSPA6 PE=1 SV=2
G3XAE9;Q9Y4F4;H0YIY7	7	4	31.5158	5.18E-05	8.60E-05	1.949806	1	2D	HYS	196689.0612	KIAA0423_ isoform CRA_a OS=Homo sapiens OX=9606 GN=TOGARAM1 PE=1 SV=1
A0A075B7B4;Q14164;A0A075B7C7	4	4	20.3	3.53E-06	8.95E-06	3.159018	1	2D	0.3PG	74434.8811	Inhibitor of nuclear factor kappa-B kinase subunit epsilon OS=Homo sapiens OX=9606 GN=IKBKE PE=1 SV=1
P02766;A0A087WT59	6	4	80.6835	2.04E-09	8.82E-08	6.646789	1	0.3PG	2D	16001.0979	Transthyretin OS=Homo sapiens OX=9606 GN=TTR PE=1 SV=1
Q75QN2;J3KNV5;E5RG48;E5RIN8;E	4	4	27.1001	1.09E-05	2.28E-05	2.067881	1	HYS	0.3PG	114570.9629	Integrator complex subunit 8 OS=Homo sapiens OX=9606 GN=INTS8 PE=1 SV=1
P31947	6	4	74.6939	0.00464	0.005294	1.459836	0.96553	2D	0.3PG	27888.1757	14-3-3 protein sigma OS=Homo sapiens OX=9606 GN=SFN PE=1 SV=1
Q86W92	5	4	27.417	4.03E-06	9.97E-06	2.08642	1	0.3PG	2D	114594.643	Liprin-beta-1 OS=Homo sapiens OX=9606 GN=PPFIBP1 PE=1 SV=2
Q38SD2;E9PK39;E9PMK9;E9PLF8	5	4	23.2528	6.50E-06	1.46E-05	2.99834	1	0.3PG	2D	228529.7019	Leucine-rich repeat serine/threonine-protein kinase 1 OS=Homo sapiens OX=9606 GN=LRRK1 PE=1 SV=3
Q9UG01	4	4	20.1157	2.37E-05	4.38E-05	2.36256	1	2D	0.3PG	199344.0591	Intraflagellar transport protein 172 homolog OS=Homo sapiens OX=9606 GN=IFT172 PE=1 SV=2
P54762;A0A3B3IRY8;C9J466;C9J6S	5	4	32.8658	2.65E-07	1.44E-06	5.41722	1	0.3PG	2D	111368.1547	Ephrin type-B receptor 1 OS=Homo sapiens OX=9606 GN=EPHB1 PE=1 SV=1
A0A7I2V5M3;P07711;A0A7I2V484;	6	4	46.6547	2.53E-06	6.99E-06	3.70878	1	HYS	0.3PG	29798.6522	Procathepsin L OS=Homo sapiens OX=9606 GN=CTSL PE=1 SV=1
Q05682;C9J813;E7EX44;E9PGZ1;C9	6	4	40.3124	4.12E-06	1.01E-05	4.679541	1	2D	0.3PG	93288.2192	Caldesmon OS=Homo sapiens OX=9606 GN=CALD1 PE=1 SV=3
Q14651;C9JAM8;C9J0F3;C9J359	11	4	60.0745	3.58E-06	9.04E-06	4.126166	1	0.3PG	2D	70652.7593	Plastin-1 OS=Homo sapiens OX=9606 GN=PLS1 PE=1 SV=2
P55884;C9JQN7	5	4	24.8926	8.67E-05	0.000133	5.595654	1	2D	HYS	92880.941	Eukaryotic translation initiation factor 3 subunit B OS=Homo sapiens OX=9606 GN=EIF3B PE=1 SV=3
Q5W041;B4DXS3	4	4	21.5742	7.52E-06	1.66E-05	2.518026	1	HYS	0.3PG	97203.4253	Armadillo repeat-containing protein 3 OS=Homo sapiens OX=9606 GN=ARMC3 PE=2 SV=2
Q9H299;Q5T123	4	4	40.5597	9.61E-05	0.000145	2.591919	1	HYS	0.3PG	10494.7603	SH3 domain-binding glutamic acid-rich-like protein 3 OS=Homo sapiens OX=9606 GN=SH3BGRL3 PE=1 SV=1
E9PIT3;P00734	4	4	23.6732	2.31E-07	1.38E-06	10.2917	1	0.3PG	HYS	66834.2775	Activation peptide fragment 1 OS=Homo sapiens OX=9606 GN=F2 PE=1 SV=1
P26022	6	4	56.6542	8.78E-06	1.88E-05	4.367659	1	2D	0.3PG	42545.9553	Pentraxin-related protein PTX3 OS=Homo sapiens OX=9606 GN=PTX3 PE=1 SV=3
Q10471	5	4	24.5862	6.81E-06	1.52E-05	3.575791	1	2D	0.3PG	65474.241	Polypeptide N-acetylgalactosaminyltransferase 2 OS=Homo sapiens OX=9606 GN=GALNT2 PE=1 SV=1
P78539	5	4	39.5119	7.83E-07	2.81E-06	4.86562	1	2D	0.3PG	52826.6319	Sushi repeat-containing protein SRPX OS=Homo sapiens OX=9606 GN=SRPX PE=1 SV=1
Q9Y5Q9;A0A494C1S7	5	4	21.7405	0.000449	0.000575	1.31228	0.999947	HYS	2D	102070.8449	General transcription factor 3C polypeptide 3 OS=Homo sapiens OX=9606 GN=GTF3C3 PE=1 SV=1
Q8N3U4	6	4	33.6165	3.80E-05	6.58E-05	4.258099	1	0.3PG	2D	142524.3553	Cohesin subunit SA-2 OS=Homo sapiens OX=9606 GN=STAG2 PE=1 SV=3
P04083;Q5T3N1;Q5T3N0	5	4	32.1666	5.01E-07	2.07E-06	4.17251	1	HYS	0.3PG	38942.4262	Annexin A1 OS=Homo sapiens OX=9606 GN=ANXA1 PE=1 SV=2
H3BP20;P06865;H3BS10;H3BTD4;A	9	4	57.2113	0.000158	0.000222	2.700828	1	2D	0.3PG	62455.6136	Beta-hexosaminidase OS=Homo sapiens OX=9606 GN=HEXA PE=1 SV=1
Q15155;A0A087X117;A0A0G2JN29	8	4	39.8679	1.59E-05	3.11E-05	34.96669	1	HYS	0.3PG	135293.5922	Nodal modulator 1 OS=Homo sapiens OX=9606 GN=NOMO1 PE=1 SV=5
H7BZA0	8	4	43.7466	0.001805	0.002134	1.667131	0.995005	2D	0.3PG	80855.1077	Neurobeachin-like protein 1 (Fragment) OS=Homo sapiens OX=9606 GN=NBEAL1 PE=1 SV=1
Q9Y3P9;B5MCD9	5	4	21.3434	0.001248	0.001505	3.646595	0.998121	2D	0.3PG	122991.6608	Rab GTPase-activating protein 1 OS=Homo sapiens OX=9606 GN=RABGAP1 PE=1 SV=3
P06748;A0A7I2V5S2;A0A7I2YQC0;A	5	4	39.2664	9.50E-05	0.000143	1.797199	1	2D	0.3PG	32746.1672	Nucleophosmin OS=Homo sapiens OX=9606 GN=NPM1 PE=1 SV=2
E7EVA0	8	4	48.8518	4.72E-06	1.12E-05	2.195525	1	0.3PG	2D	246699.4101	Microtubule-associated protein OS=Homo sapiens OX=9606 GN=MAP4 PE=1 SV=1
A0A7P0TB70;O95786;A0A7P0T9I8;	5	4	37.735	0.002995	0.003478	1.461465	0.984565	2D	HYS	102014.6776	Uncharacterized protein OS=Homo sapiens OX=9606 PE=4 SV=1
Q8WZ64;D6RAD6	7	4	38.5887	1.42E-05	2.84E-05	5.379237	1	2D	0.3PG	195219.9231	Arf-GAP with Rho-GAP domain_ ANK repeat and PH domain-containing protein 2 OS=Homo sapiens OX=9606 GN=ARAP2 PE=1 SV=3
P40926;G3XAL0	5	4	36.6274	1.04E-07	8.55E-07	3.548379	1	2D	0.3PG	35959.5534	Malate dehydrogenase_ mitochondrial OS=Homo sapiens OX=9606 GN=MDH2 PE=1 SV=3
A0A0B4J2C3;P13693;Q5W0H4;J3KI	5	4	62.7519	1.29E-06	4.13E-06	2.572731	1	HYS	0.3PG	22802.0048	Translationally-controlled tumor protein OS=Homo sapiens OX=9606 GN=TPT1 PE=1 SV=1

Q13162;H7C3T4;A6NG45;A6NJJ0	5	4	35.0232	5.12E-05	8.54E-05	2.398136	1	HYS	0.3PG	30768.044	Peroxiredoxin-4 OS=Homo sapiens OX=9606 GN=PRDX4 PE=1 SV=1
O00299	8	4	53.8424	3.82E-07	1.84E-06	1.828824	1	HYS	2D	27264.9152	Chloride intracellular channel protein 1 OS=Homo sapiens OX=9606 GN=CLIC1 PE=1 SV=4
A0A1B0GTN9;E9PKC0;Q6IQ23	5	4	28.3959	0.000168	0.000235	1.520483	1	HYS	0.3PG	135938.1043	Pleckstrin homology domain-containing family A member 7 (Fragment) OS=Homo sapiens OX=9606 GN=PLEKHA7 PE=1 SV=1
Q96J65	8	4	44.2562	1.16E-06	3.80E-06	6.285954	1	HYS	0.3PG	153723.4093	ATP-binding cassette sub-family C member 12 OS=Homo sapiens OX=9606 GN=ABCC12 PE=1 SV=2
Q96J02;A0A590UJQ1;A0A590UJW	9	4	43.3117	1.71E-06	5.17E-06	2.367497	1	2D	0.3PG	103658.3626	E3 ubiquitin-protein ligase Itchy homolog OS=Homo sapiens OX=9606 GN=ITCH PE=1 SV=2
P36776;K7EJE8;K7EKE6	6	3	40.1494	0.002227	0.002614	1.420802	0.9918	HYS	0.3PG	107002.734	Lon protease homolog_ mitochondrial OS=Homo sapiens OX=9606 GN=LONP1 PE=1 SV=2
Q8TF44	6	3	32.7792	0.003256	0.003755	1.276097	0.981804	HYS	0.3PG	44804.403	C2 calcium-dependent domain-containing protein 4C OS=Homo sapiens OX=9606 GN=C2CD4C PE=1 SV=2
Q12851;H7C208	6	3	38.9636	1.06E-07	8.68E-07	8.815042	1	HYS	0.3PG	92525.5385	Mitogen-activated protein kinase kinase kinase kinase 2 OS=Homo sapiens OX=9606 GN=MAP4K2 PE=1 SV=2
A0A6Q8PF29;A0A6Q8PF44;A0A6Q	6	3	36.4142	5.23E-05	8.66E-05	1.649299	1	HYS	0.3PG	125275.9693	Inverted formin-2 OS=Homo sapiens OX=9606 GN=INF2 PE=1 SV=1
P14174	3	3	36.5675	0.025503	0.027097	1.127709	0.740583	2D	0.3PG	12647.4113	Macrophage migration inhibitory factor OS=Homo sapiens OX=9606 GN=MIF PE=1 SV=4
Q7KZF4	6	3	30.3848	0.001324	0.001594	1.785144	0.997781	2D	HYS	102681.4413	Staphylococcal nuclease domain-containing protein 1 OS=Homo sapiens OX=9606 GN=SND1 PE=1 SV=1
Q14005;H0YKB7;H0YLL1;H3BN12;H	6	3	27.4958	0.00911	0.010017	1.720785	0.907924	2D	0.3PG	143064.1528	Pro-interleukin-16 OS=Homo sapiens OX=9606 GN=IL16 PE=1 SV=4
Q6DN14;D6RA42	4	3	19.3493	6.47E-09	1.67E-07	9.574845	1	0.3PG	2D	112479.8813	Multiple C2 and transmembrane domain-containing protein 1 OS=Homo sapiens OX=9606 GN=MCTP1 PE=2 SV=2
A0A0B4J1T8;Q9UF33;B4DXQ6	4	3	18.0252	1.95E-06	5.70E-06	2.43432	1	HYS	2D	128329.7039	Receptor protein-tyrosine kinase OS=Homo sapiens OX=9606 GN=EPHA6 PE=1 SV=1
J3QR65;J3QRJ2;Q9NYA4;J3KT95;J3	4	3	26.8605	0.090098	0.092828	1.321271	0.454558	0.3PG	2D	129522.1998	Protein-tyrosine-phosphatase OS=Homo sapiens OX=9606 GN=MTMR4 PE=1 SV=1
A0A3B3IU48;Q8NFP4;A0A7P0TB21	6	3	28.8188	0.04145	0.043284	1.309943	0.634969	HYS	2D	109164.3267	MAM domain-containing glycosylphosphatidylinositol anchor protein 1 OS=Homo sapiens OX=9606 GN=MDGA1 PE=1 SV=1
P62942;A0A087WZM5;A0A7I2V3S	4	3	25.0614	0.000148	0.000211	1.55551	1	0.3PG	2D	12007.7621	Peptidyl-prolyl cis-trans isomerase FKBP1A OS=Homo sapiens OX=9606 GN=FKBP1A PE=1 SV=2
Q9UHP3	6	3	26.9153	2.23E-06	6.34E-06	2.064247	1	2D	0.3PG	122902.4051	Ubiquitin carboxyl-terminal hydrolase 25 OS=Homo sapiens OX=9606 GN=USP25 PE=1 SV=4
E9PJA2;P09603;E9PKP4;E9PQ08;H	3	3	19.6998	3.65E-06	9.18E-06	2.575934	1	2D	0.3PG	31741.3069	Macrophage colony-stimulating factor 1 (Fragment) OS=Homo sapiens OX=9606 GN=CSF1 PE=1 SV=1
P15090;E5RH45;E5RIR0;P02689	6	3	36.6324	6.96E-06	1.55E-05	2.079469	1	0.3PG	2D	14832.9782	Fatty acid-binding protein_ adipocyte OS=Homo sapiens OX=9606 GN=FABP4 PE=1 SV=3
Q8NB78;H0Y6H0	3	3	13.4919	0.001676	0.001987	1.468514	0.995849	HYS	0.3PG	93524.1359	Lysine-specific histone demethylase 1B OS=Homo sapiens OX=9606 GN=KDM1B PE=1 SV=3
Q9NYU2	6	3	33.2489	0.002976	0.00346	1.60848	0.984768	2D	0.3PG	177931.2185	UDP-glucose:glycoprotein glucosyltransferase 1 OS=Homo sapiens OX=9606 GN=UGGT1 PE=1 SV=3
O76061	3	3	16.4195	1.36E-08	2.32E-07	11.41151	1	2D	0.3PG	34104.025	Stanniocalcin-2 OS=Homo sapiens OX=9606 GN=STC2 PE=1 SV=1
A0A7P0TAE5;Q9C0F0;A0A2R8Y461	8	3	39.495	1.15E-05	2.36E-05	4.555064	1	2D	0.3PG	240177.5289	Putative Polycomb group protein ASXL3 OS=Homo sapiens OX=9606 GN=ASXL3 PE=4 SV=1
A0A5F9UP49;Q9BRK5;A0A5F9UJX7	3	3	30.9482	8.20E-07	2.91E-06	5.73074	1	2D	0.3PG	41050.8543	45 kDa calcium-binding protein OS=Homo sapiens OX=9606 GN=SDF4 PE=1 SV=1
O43314;A0A087WZV0	5	3	21.0733	4.85E-07	2.02E-06	6.674548	1	2D	0.3PG	141661.5445	Inositol hexakisphosphate and diphosphoinositol-pentakisphosphate kinase 2 OS=Homo sapiens OX=9606 GN=PPIP5K2 PE=1 SV=3
Q9BUR4;E9PMG4	3	3	12.8438	5.34E-05	8.83E-05	1.921434	1	2D	0.3PG	60164.8699	Telomerase Cajal body protein 1 OS=Homo sapiens OX=9606 GN=WRAP53 PE=1 SV=1
O60518	5	3	27.0087	0.000682	0.000846	1.537848	0.99973	HYS	0.3PG	126253.5538	Ran-binding protein 6 OS=Homo sapiens OX=9606 GN=RANBP6 PE=1 SV=2
Q8IWY7	7	3	40.1398	3.90E-06	9.67E-06	5.380922	1	0.3PG	2D	184007.0012	Tau-tubulin kinase OS=Homo sapiens OX=9606 GN=TTBK2 PE=1 SV=1
P04217;M0R009	5	3	37.945	1.38E-06	4.37E-06	3.541025	1	0.3PG	2D	54823.8357	Alpha-1B-glycoprotein OS=Homo sapiens OX=9606 GN=A1BG PE=1 SV=4
P78324	3	3	16.2706	2.19E-05	4.14E-05	2.892558	1	2D	0.3PG	55480.1547	Tyrosine-protein phosphatase non-receptor type substrate 1 OS=Homo sapiens OX=9606 GN=SIRPA PE=1 SV=2
A0A0G2JPA8;P08697;A0A0J9YWQ3	3	3	30.37	6.77E-05	0.000107	2.905	1	0.3PG	2D	48165.2503	Alpha-2-antiplasmin OS=Homo sapiens OX=9606 GN=SERPINF2 PE=1 SV=1
Q14693;A0A0A0MS66	3	3	18.9577	2.29E-05	4.29E-05	3.171793	1	0.3PG	2D	99348.6419	Phosphatidate phosphatase LPIN1 OS=Homo sapiens OX=9606 GN=LPIN1 PE=1 SV=2
A0A6Q8KRG2;Q08379;A0A1W2PQ	3	3	15.1602	0.004504	0.005153	1.225917	0.967209	HYS	0.3PG	112285.9808	Golgin subfamily A member 2 OS=Homo sapiens OX=9606 GN=GOLGA2 PE=1 SV=3
P17987	5	3	26.3179	7.29E-07	2.68E-06	4.681169	1	2D	0.3PG	60856.9388	T-complex protein 1 subunit alpha OS=Homo sapiens OX=9606 GN=TCP1 PE=1 SV=1
Q9NZ56;A0A7P0TA49;A0A7P0Z432	5	3	24.6394	3.38E-09	1.17E-07	9.211453	1	0.3PG	HYS	181303.3085	Formin-2 OS=Homo sapiens OX=9606 GN=FMN2 PE=1 SV=4
Q9HAS0;J9JIC5;K7EII1	5	3	19.2751	0.012723	0.013843	1.203826	0.864018	0.3PG	2D	45305.9377	Protein Njmu-R1 OS=Homo sapiens OX=9606 GN=C17orf75 PE=1 SV=2
G5E9A6;P51784	5	3	26.9422	5.32E-07	2.13E-06	4.492323	1	0.3PG	2D	106057.688	Ubiquitin carboxyl-terminal hydrolase OS=Homo sapiens OX=9606 GN=USP11 PE=1 SV=1
A0A024QZX5;A0A087X1N8;P35237	5	3	25.1228	4.91E-05	8.23E-05	2.800506	1	HYS	2D	43366.7061	Serpin B6 OS=Homo sapiens OX=9606 GN=SERPINB6 PE=1 SV=1
Q14674	5	3	27.8321	1.97E-06	5.73E-06	3.110418	1	2D	0.3PG	236711.405	Separin OS=Homo sapiens OX=9606 GN=ESPL1 PE=1 SV=3
A0A499FI48;P13667	5	3	24.0925	1.18E-05	2.41E-05	2.090818	1	HYS	0.3PG	73402.8644	Protein disulfide-isomerase OS=Homo sapiens OX=9606 GN=PDIA4 PE=1 SV=1
Q2TAZ0	5	3	38.6721	6.25E-07	2.43E-06	7.036537	1	2D	0.3PG	215027.4191	Autophagy-related protein 2 homolog A OS=Homo sapiens OX=9606 GN=ATG2A PE=1 SV=3
A0A087X0R0;A0A6Q8PFK0;O1555	3	3	14.6468	0.296163	0.299689	1.117863	0.210944	0.3PG	2D	161727.0855	Lysine-specific demethylase 6A OS=Homo sapiens OX=9606 GN=KDM6A PE=1 SV=2
Q8NEZ3;D6R9P6;D6RAI4;D6RE75	5	3	23.2593	1.59E-06	4.85E-06	2.323709	1	HYS	2D	153520.2271	WD repeat-containing protein 19 OS=Homo sapiens OX=9606 GN=WDR19 PE=1 SV=2
A0A7P0MRI8;Q15147	5	3	22.4003	0.000197	0.00027	1.697678	0.999999	0.3PG	2D	138685.3405	1-phosphatidylinositol 4_5-bisphosphate phosphodiesterase beta-4 OS=Homo sapiens OX=9606 GN=PLCB4 PE=4 SV=1
P09871;F8WCZ6;H0Y5D1;F5H7T4	22	3	208.6328	1.15E-06	3.78E-06	3.028627	1	2D	0.3PG	78224.2859	Complement C1s subcomponent OS=Homo sapiens OX=9606 GN=C1S PE=1 SV=1
Q8WWH4	3	3	19.3065	0.007333	0.008201	1.436173	0.930798	2D	0.3PG	53971.5666	Ankyrin repeat_ SAM and basic leucine zipper domain-containing protein 1 OS=Homo sapiens OX=9606 GN=ASZ1 PE=1 SV=1
P24534;C9JZW3;F2Z2G2;F8WF65	3	3	25.8588	0.000207	0.000282	1.781603	0.999999	2D	0.3PG	24934.8442	Elongation factor 1-beta OS=Homo sapiens OX=9606 GN=EEF1B2 PE=1 SV=3
O00311	5	3	22.2767	2.54E-06	6.99E-06	4.310808	1	HYS	0.3PG	64686.6938	Cell division cycle 7-related protein kinase OS=Homo sapiens OX=9606 GN=CDC7 PE=1 SV=1
A0A3B3IS84;A0A3B3IT15;P53621;A	5	3	35.1541	1.05E-05	2.19E-05	2.167971	1	2D	0.3PG	137649.9755	Coatomer subunit alpha OS=Homo sapiens OX=9606 GN=COPA PE=1 SV=1
Q8IZ83	3	3	14.9638	3.15E-05	5.56E-05	7.398294	1	0.3PG	HYS	86154.0532	Aldehyde dehydrogenase family 16 member A1 OS=Homo sapiens OX=9606 GN=ALDH16A1 PE=1 SV=2
P30085;A0A494BXC7;Q5T0D2	5	3	31.4368	0.00074	0.00091	2.119008	0.999639	HYS	2D	22450.4849	UMP-CMP kinase OS=Homo sapiens OX=9606 GN=CMPK1 PE=1 SV=3
A0A1B0GVU7;H0YMT1;H0YN01	5	3	22.9557	4.16E-08	4.77E-07	7.228136	1	2D	0.3PG	166228.6088	Talin-2 OS=Homo sapiens OX=9606 GN=TLN2 PE=1 SV=1
B1AK87;B1AK88;P47756;B1AK85	3	3	26.2043	0.000898	0.001097	1.985889	0.999308	2D	0.3PG	27725.2774	F-actin-capping protein subunit beta OS=Homo sapiens OX=9606 GN=CAPZB PE=1 SV=2
A0A0A0MQU6;B3KU01;E9PDV9;H0	3	3	19.8373	5.31E-06	1.24E-05	189.7502	1	2D	HYS	117621.663	Sema domain_ transmembrane domain (TM)_ and cytoplasmic domain_ (Semaphorin) 6A_ isoform CRA_d OS=Homo sapiens OX=9606 GN=SEMA6A PE=1 SV=1
Q9UBF2	6	3	38.315	0.002904	0.003381	1.396015	0.985505	0.3PG	2D	98763.0882	Coatomer subunit gamma-2 OS=Homo sapiens OX=9606 GN=COPG2 PE=1 SV=1
O76013;Q14532	4	3	30.4779	1.39E-05	2.78E-05	2.43723	1	0.3PG	2D	53387.6796	Keratin_ type I cuticular Ha6 OS=Homo sapiens OX=9606 GN=KRT36 PE=1 SV=1
Q96RQ9	4	3	30.3371	8.74E-07	3.07E-06	5.337677	1	2D	0.3PG	63109.2037	L-amino-acid oxidase OS=Homo sapiens OX=9606 GN=IL4I1 PE=1 SV=1
P07949	4	3	21.7242	0.001438	0.001719	1.391125	0.99722	HYS	0.3PG	126257.7771	Proto-oncogene tyrosine-protein kinase receptor Ret OS=Homo sapiens OX=9606 GN=RET PE=1 SV=3
Q9Y5F7	8	3	41.3137	0.000412	0.000531	5.84327	0.999963	2D	0.3PG	102012.1861	Protocadherin gamma-C4 OS=Homo sapiens OX=9606 GN=PCDHGC4 PE=2 SV=1
P17948;H9N1E7	7	3	48.2504	3.58E-07	1.75E-06	2.486387	1	2D	0.3PG	152650.8081	Vascular endothelial growth factor receptor 1 OS=Homo sapiens OX=9606 GN=FLT1 PE=1 SV=2
A0A590UJC7;A0A590UK51;O60733	4	3	17.0929	2.38E-05	4.39E-05	11.61494	1	2D	0.3PG	86034.001	85/88 kDa calcium-independent phospholipase A2 OS=Homo sapiens OX=9606 GN=PLA2G6 PE=1 SV=1
H0YFN7;Q4ADV7	4	3	24.4586	0.031569	0.033292	1.439229	0.695617	2D	0.3PG	148669.8694	Protein RIC1 homolog (Fragment) OS=Homo sapiens OX=9606 GN=RIC1 PE=1 SV=1
Q562R1	10	3	96.7621	3.12E-06	8.22E-06	5.781614	1	HYS	2D	42345.4528	Beta-actin-like protein 2 OS=Homo sapiens OX=9606 GN=ACTBL2 PE=1 SV=2
A0A2U3TZH3;Q05639;A0A2R8Y488	7	3	47.5526	1.37E-05	2.76E-05	4.549104	1	2D	0.3PG	54682.8088	Elongation factor 1-alpha OS=Homo sapiens OX=9606 GN=EEF1A2 PE=1 SV=1
Q9HCU0	4	3	24.4342	9.00E-10	5.10E-08	3.273836	1	2D	0.3PG	82855.2795	Endosialin OS=Homo sapiens OX=9606 GN=CD248 PE=1 SV=1
P23528;E9PK25;E9PP50;G3V1A4;E	7	3	102.4015	1.55E-07	1.09E-06	3.108268	1	HYS	0.3PG	18730.6344	Cofilin-1 OS=Homo sapiens OX=9606 GN=CFL1 PE=1 SV=3
P60842;J3KT12;J3QS69;J3KSZ0;J3K	7	3	51.377	7.19E-05	0.000113	2.996668	1	2D	0.3PG	46382.1618	Eukaryotic initiation factor 4A-I OS=Homo sapiens OX=9606 GN=EIF4A1 PE=1 SV=1
P0DP23;P0DP24;P0DP25;M0QZ52;	8	3	64.5364	1.03E-06	3.50E-06	3.406051	1	HYS	2D	16837.6523	Calmodulin-1 OS=Homo sapiens OX=9606 GN=CALM1 PE=1 SV=1
P34931;Q53FA3	10	3	74.7339	4.46E-05	7.56E-05	3.043352	1	HYS	0.3PG	70774.3427	Heat shock 70 kDa protein 1-like OS=Homo sapiens OX=9606 GN=HSPA1L PE=1 SV=2
A0A0A0MQX5;O94779	4	3	23.923	5.36E-08	5.43E-07	10.43795	1	0.3PG	HYS	119611.4592	Contactin-5 OS=Homo sapiens OX=9606 GN=CNTN5 PE=1 SV=1
Q8IYE0;C9JRR4	4	3	19.4986	1.14E-06	3.76E-06	1.871352	1	0.3PG	2D	113205.9008	Coiled-coil domain-containing protein 146 OS=Homo sapiens OX=9606 GN=CCDC146 PE=1 SV=2

A0A5H1ZRS1;Q15154;A0A4W8VX1	7	3	33.6398	0.000146	0.000208	35.24919	1	2D	0.3PG	229385.3913	Pericentriolar material 1 protein OS=Homo sapiens OX=9606 GN=PCM1 PE=1 SV=1
Q5XKE5;H0YID6	10	3	77.7954	1.64E-07	1.12E-06	1.831394	1	HYS	0.3PG	58120.821	Keratin_ type II cytoskeletal 79 OS=Homo sapiens OX=9606 GN=KRT79 PE=1 SV=2
A0A7P0T7Z1;A0A7P0T871;A0A7P0	5	3	25.8131	1.64E-08	2.64E-07	11.8859	1	0.3PG	2D	68609.3585	Complex I assembly factor ACAD9_ mitochondrial OS=Homo sapiens OX=9606 GN=ACAD9 PE=4 SV=1
O75083	4	3	21.859	0.020385	0.021823	1.244941	0.784612	2D	0.3PG	66877.9275	WD repeat-containing protein 1 OS=Homo sapiens OX=9606 GN=WDR1 PE=1 SV=4
P08185;G3V350	4	3	37.5959	6.54E-07	2.50E-06	23.38613	1	0.3PG	2D	45312.064	Corticosteroid-binding globulin OS=Homo sapiens OX=9606 GN=SERPINA6 PE=1 SV=1
Q15084	4	3	37.0194	8.97E-07	3.14E-06	5.605968	1	2D	0.3PG	48520.5659	Protein disulfide-isomerase A6 OS=Homo sapiens OX=9606 GN=PDIA6 PE=1 SV=1
H0Y5C6	9	3	61.9352	0.000688	0.000851	1.531168	0.999722	HYS	0.3PG	30087.3257	Filamin-A (Fragment) OS=Homo sapiens OX=9606 GN=FLNA PE=1 SV=1
P13647;H0YI76;H0YIN9;F8VV57	8	3	58.7874	0.000405	0.000524	79.84367	0.999966	0.3PG	HYS	62606.5615	Keratin_ type II cytoskeletal 5 OS=Homo sapiens OX=9606 GN=KRT5 PE=1 SV=3
A0A6Q8PF58;C9J126;C9JMH2;P19	4	3	24.4892	8.76E-05	0.000134	2.273423	1	2D	0.3PG	81660.4292	Cadherin-2 OS=Homo sapiens OX=9606 GN=CDH2 PE=1 SV=1
Q5TB80	8	3	37.7714	3.61E-07	1.75E-06	5.323747	1	0.3PG	2D	162456.5274	Centrosomal protein of 162 kDa OS=Homo sapiens OX=9606 GN=CEP162 PE=1 SV=2
P67936;A0A2R8Y5V9;A0A2R8YGX3	8	3	61.1479	1.18E-05	2.41E-05	2.64592	1	2D	0.3PG	28635.913	Tropomyosin alpha-4 chain OS=Homo sapiens OX=9606 GN=TPM4 PE=1 SV=3
P09917;A0A087X109	4	3	22.349	0.000457	0.000584	1.776734	0.999943	HYS	0.3PG	78724.7192	Polyunsaturated fatty acid 5-lipoxygenase OS=Homo sapiens OX=9606 GN=ALOX5 PE=1 SV=2
O00443	4	3	17.8751	3.14E-06	8.25E-06	2.710604	1	2D	0.3PG	192276.5953	Phosphatidylinositol 4-phosphate 3-kinase C2 domain-containing subunit alpha OS=Homo sapiens OX=9606 GN=PIK3C2A PE=1 SV=2
D6RGZ6	9	3	75.5494	3.98E-07	1.86E-06	4.800776	1	2D	0.3PG	136485.7851	Versican core protein (Fragment) OS=Homo sapiens OX=9606 GN=VCAN PE=1 SV=1
Q15008	4	3	18.0431	2.13E-06	6.13E-06	3.317156	1	2D	0.3PG	45816.5696	26S proteasome non-ATPase regulatory subunit 6 OS=Homo sapiens OX=9606 GN=PSMD6 PE=1 SV=1
P09104;F5H0C8;U3KQP4	9	3	82.2944	1.43E-07	1.04E-06	2.672894	1	2D	0.3PG	47610.8108	Gamma-enolase OS=Homo sapiens OX=9606 GN=ENO2 PE=1 SV=3
B2RC85;P0C881	4	3	19.5363	4.78E-07	2.00E-06	4.588768	1	2D	0.3PG	101288.2419	Radial spoke head 10 homolog B2 OS=Homo sapiens OX=9606 GN=RSPH10B2 PE=2 SV=2
Q8TBP0;I3L4U4;I3L0V9;I3L4T6	4	3	21.923	1.82E-07	1.19E-06	5.38599	1	0.3PG	2D	87455.3682	TBC1 domain family member 16 OS=Homo sapiens OX=9606 GN=TBC1D16 PE=2 SV=1
O43306;B3KWA8;O95622	4	3	24.4906	0.000605	0.00076	1.493534	0.999826	HYS	2D	132383.5116	Adenylate cyclase type 6 OS=Homo sapiens OX=9606 GN=ADCY6 PE=1 SV=2
P01042	4	3	27.9716	5.38E-08	5.43E-07	10.41957	1	0.3PG	2D	73040.9754	Kininogen-1 OS=Homo sapiens OX=9606 GN=KNG1 PE=1 SV=2
Q92766;C9JU34	4	3	24.8448	7.89E-10	5.10E-08	12.40144	1	0.3PG	2D	184271.8527	Ras-responsive element-binding protein 1 OS=Homo sapiens OX=9606 GN=RREB1 PE=1 SV=3
Q9Y5F6	4	3	18.652	1.69E-05	3.29E-05	4.472997	1	HYS	0.3PG	102605.1472	Protocadherin gamma-C5 OS=Homo sapiens OX=9606 GN=PCDHGC5 PE=2 SV=1
Q8TF05;J3QLA6	5	3	30.6723	0.017266	0.018601	1.456032	0.814775	HYS	0.3PG	108429.8904	Serine/threonine-protein phosphatase 4 regulatory subunit 1 OS=Homo sapiens OX=9606 GN=PPP4R1 PE=1 SV=1
A0A3B3IU69;F8W717;O00423;H0Y	3	3	14.3409	7.99E-05	0.000124	2.89331	1	0.3PG	2D	93830.2749	Echinoderm microtubule-associated protein-like 1 OS=Homo sapiens OX=9606 GN=EML1 PE=1 SV=1
Q9NZL6	3	3	16.7795	4.00E-08	4.66E-07	27.82731	1	0.3PG	2D	87670.9497	Ral guanine nucleotide dissociation stimulator-like 1 OS=Homo sapiens OX=9606 GN=RGL1 PE=1 SV=1
E5RJ29;Q9NYI0	3	3	15.4262	0.008275	0.009171	1.27223	0.918592	2D	HYS	109675.3118	PH and SEC7 domain-containing protein 3 OS=Homo sapiens OX=9606 GN=PSD3 PE=1 SV=1
Q9H8L6;V9GY37	4	3	28.0801	5.03E-06	1.17E-05	3.761738	1	HYS	0.3PG	105093.0134	Multimerin-2 OS=Homo sapiens OX=9606 GN=MMRN2 PE=1 SV=2
Q149N8;A0A0D9SFM0;H7C2W2	6	3	28.0076	5.57E-08	5.51E-07	3.644975	1	HYS	0.3PG	195702.899	E3 ubiquitin-protein ligase SHPRH OS=Homo sapiens OX=9606 GN=SHPRH PE=1 SV=2
P35913;H7C4P9;H7C4F7	6	3	31.013	2.59E-07	1.44E-06	6.003624	1	2D	0.3PG	99533.5529	Rod cGMP-specific 3'_5'-cyclic phosphodiesterase subunit beta OS=Homo sapiens OX=9606 GN=PDE6B PE=2 SV=2
Q14974;J3KTM9;J3QRG4	3	3	16.497	9.72E-05	0.000146	3.537875	1	2D	0.3PG	98481.9966	Importin subunit beta-1 OS=Homo sapiens OX=9606 GN=KPNB1 PE=1 SV=2
O95678	6	3	52.6519	5.93E-05	9.63E-05	2.00202	1	HYS	0.3PG	59845.6071	Keratin_ type II cytoskeletal 75 OS=Homo sapiens OX=9606 GN=KRT75 PE=1 SV=2
O00750;F5GWN5	6	3	40.1464	8.99E-06	1.92E-05	2.840601	1	2D	0.3PG	186935.1426	Phosphatidylinositol 4-phosphate 3-kinase C2 domain-containing subunit beta OS=Homo sapiens OX=9606 GN=PIK3C2B PE=1 SV=2
A0A075B6T1;Q9C0C7	3	3	15.2784	6.28E-06	1.41E-05	2.705227	1	2D	HYS	130169.1438	Activating molecule in BECN1-regulated autophagy protein 1 OS=Homo sapiens OX=9606 GN=AMBRA1 PE=1 SV=1
P12277	3	3	12.1261	9.87E-06	2.07E-05	1.697857	1	HYS	0.3PG	42929.5023	Creatine kinase B-type OS=Homo sapiens OX=9606 GN=CKB PE=1 SV=1
A0A2R8Y590;H7BYL6	4	3	26.948	1.76E-07	1.17E-06	7.029372	1	2D	0.3PG	247036.8676	A-kinase anchor protein 9 OS=Homo sapiens OX=9606 GN=AKAP9 PE=1 SV=2
Q9H7F0;A0A2R8Y635;A0A2R8YDN	6	3	38.4807	6.15E-05	9.93E-05	3.087282	1	0.3PG	HYS	139868.0252	Polyamine-transporting ATPase 13A3 OS=Homo sapiens OX=9606 GN=ATP13A3 PE=1 SV=4
P07686;H0Y9B6;Q5URX0	6	3	45.0641	3.40E-07	1.72E-06	2.751096	1	2D	0.3PG	63567.6417	Beta-hexosaminidase subunit beta OS=Homo sapiens OX=9606 GN=HEXB PE=1 SV=3
H0YDM2;O94964;X6R3R3	6	3	41.9543	7.91E-05	0.000123	1.778331	1	0.3PG	2D	143541.6565	Protein SOGA1 (Fragment) OS=Homo sapiens OX=9606 GN=SOGA1 PE=1 SV=1
P20618	3	3	21.3648	0.029179	0.03081	1.783865	0.71251	0.3PG	HYS	26717.5428	Proteasome subunit beta type-1 OS=Homo sapiens OX=9606 GN=PSMB1 PE=1 SV=2
P52907	4	3	30.4587	2.01E-05	3.83E-05	2.293625	1	2D	0.3PG	33093.8919	F-actin-capping protein subunit alpha-1 OS=Homo sapiens OX=9606 GN=CAPZA1 PE=1 SV=3
O75334;G3V200	6	3	28.9638	0.000182	0.000253	2.897873	0.999999	HYS	0.3PG	143804.1226	Liprin-alpha-2 OS=Homo sapiens OX=9606 GN=PPFIA2 PE=1 SV=2
B1APY4;Q01974	4	3	26.6496	3.47E-06	8.84E-06	2.853672	1	0.3PG	2D	80867.5482	Receptor tyrosine kinase-like orphan receptor 2_ isoform CRA_b OS=Homo sapiens OX=9606 GN=ROR2 PE=1 SV=1
Q8TDZ2	4	3	20.3947	7.11E-05	0.000112	2.240009	1	2D	0.3PG	118958.4375	[F-actin]-monooxygenase MICAL1 OS=Homo sapiens OX=9606 GN=MICAL1 PE=1 SV=2
J3KQ49;Q8WYN3	3	2	14.7256	2.55E-05	4.65E-05	3.718413	1	0.3PG	2D	69805.068	Cysteine/serine-rich nuclear protein 3 OS=Homo sapiens OX=9606 GN=CSRNP3 PE=1 SV=1
Q92870;G5E9Y1;H0YAJ5;H0YA92	3	2	14.0372	1.09E-08	2.03E-07	3.310418	1	HYS	0.3PG	84286.2698	Amyloid-beta A4 precursor protein-binding family B member 2 OS=Homo sapiens OX=9606 GN=APBB2 PE=1 SV=3
Q9UK96;J3KN78	4	2	19.5333	3.69E-06	9.23E-06	5.070574	1	2D	0.3PG	106735.3527	F-box only protein 10 OS=Homo sapiens OX=9606 GN=FBXO10 PE=1 SV=3
Q9BZ76;A0A087WZ03;A6NC89;Q9	3	2	20.1361	2.25E-06	6.36E-06	2.241799	1	HYS	2D	142857.1356	Contactin-associated protein-like 3 OS=Homo sapiens OX=9606 GN=CNTNAP3 PE=2 SV=3
A0A0G2JNY3;A0A0G2JRG8;E9PIR1;	3	2	20.5811	8.57E-05	0.000132	10.01893	1	0.3PG	HYS	99169.5126	Rho GTPase-activating protein 27 OS=Homo sapiens OX=9606 GN=ARHGAP27 PE=1 SV=1
Q6ZMI0	3	2	16.5744	6.11E-07	2.39E-06	3.142139	1	2D	0.3PG	88827.8586	Protein phosphatase 1 regulatory subunit 21 OS=Homo sapiens OX=9606 GN=PPP1R21 PE=1 SV=1
P12036	3	2	38.0848	3.88E-07	1.84E-06	12.65346	1	2D	0.3PG	112707.1217	Neurofilament heavy polypeptide OS=Homo sapiens OX=9606 GN=NEFH PE=1 SV=4
A0A087WSW9;A0A087WSY9;A0A1	3	2	21.3395	0.000142	0.000205	1.612069	1	HYS	2D	60820.3766	Thioredoxin-disulfide reductase OS=Homo sapiens OX=9606 GN=TXNRD1 PE=1 SV=1
A0A5K1VW81;S4R322	4	2	15.5225	0.009516	0.010437	1.176247	0.902786	2D	HYS	172478.3244	Centrosomal protein of 290 kDa (Fragment) OS=Homo sapiens OX=9606 GN=CEP290 PE=1 SV=2
Q96TA1	3	2	37.9424	6.87E-07	2.56E-06	18.64701	1	0.3PG	HYS	84651.2613	Protein Niban 2 OS=Homo sapiens OX=9606 GN=NIBAN2 PE=1 SV=3
Q13042	3	2	14.0291	0.000685	0.000849	1.423901	0.999725	HYS	0.3PG	72454.1547	Cell division cycle protein 16 homolog OS=Homo sapiens OX=9606 GN=CDC16 PE=1 SV=2
P02671	3	2	14.2777	8.83E-06	1.89E-05	2.347947	1	0.3PG	2D	95714.5217	Fibrinogen alpha chain OS=Homo sapiens OX=9606 GN=FGA PE=1 SV=2
C9JHW1;E7ESB6;I1E4Y6;Q6Y7W6;C	3	2	19.3112	8.64E-06	1.86E-05	2.410974	1	HYS	0.3PG	81960.0047	GRB10-interacting GYF protein 2 (Fragment) OS=Homo sapiens OX=9606 GN=GIGYF2 PE=1 SV=1
Q6UVM3;A0A3B3IRL4	3	2	12.6336	1.71E-05	3.32E-05	2.643496	1	HYS	0.3PG	132212.2489	Potassium channel subfamily T member 2 OS=Homo sapiens OX=9606 GN=KCNT2 PE=1 SV=1
P10124	3	2	25.1388	1.53E-06	4.74E-06	3.016339	1	2D	HYS	17822.9164	Serglycin OS=Homo sapiens OX=9606 GN=SRGN PE=1 SV=3
O60701	3	2	18.9783	2.82E-11	9.65E-09	50.82055	1	0.3PG	2D	55708.4853	UDP-glucose 6-dehydrogenase OS=Homo sapiens OX=9606 GN=UGDH PE=1 SV=1
Q6ZU64	3	2	19.2736	3.81E-06	9.50E-06	4.341073	1	0.3PG	HYS	220387.1717	Cilia- and flagella-associated protein 65 OS=Homo sapiens OX=9606 GN=CFAP65 PE=1 SV=2
H7C582	2	2	12.7155	0.000126	0.000183	20.03446	1	0.3PG	HYS	14078.8186	Integrator complex subunit 1 (Fragment) OS=Homo sapiens OX=9606 GN=INTS1 PE=1 SV=1
H7BYH4;P00441	2	2	14.7063	0.007877	0.008752	1.640181	0.92374	HYS	0.3PG	14080.4561	Superoxide dismutase [Cu-Zn] OS=Homo sapiens OX=9606 GN=SOD1 PE=1 SV=1
Q96CG8;E5RK99;E7EVQ5	4	2	30.9773	0.000717	0.000884	1.705932	0.999676	2D	0.3PG	26794.2635	Collagen triple helix repeat-containing protein 1 OS=Homo sapiens OX=9606 GN=CTHRC1 PE=1 SV=1
Q96R06;K7ELC8	4	2	29.2033	8.82E-05	0.000134	7.275417	1	HYS	2D	135790.7267	Sperm-associated antigen 5 OS=Homo sapiens OX=9606 GN=SPAG5 PE=1 SV=2
Q8NE28	4	2	19.1148	0.000122	0.000178	3.848951	1	2D	HYS	76475.2776	Serine/threonine kinase-like domain-containing protein STKLD1 OS=Homo sapiens OX=9606 GN=STKLD1 PE=2 SV=5
A0A3B3IS79;F5H527;Q76I76;K7EKN	3	2	27.648	3.43E-06	8.80E-06	3.20187	1	0.3PG	2D	160567.1409	Protein phosphatase Slingshot homolog 2 OS=Homo sapiens OX=9606 GN=SSH2 PE=1 SV=1
A0A7P0T9T4;A0A7P0TAK3;A0A7P0	4	2	18.9553	1.55E-07	1.09E-06	8.449419	1	0.3PG	2D	159055.6296	WD repeat-containing protein 62 OS=Homo sapiens OX=9606 GN=WDR62 PE=4 SV=1
O15090;K7EKT4	3	2	24.5337	0.000103	0.000153	6.527111	1	HYS	2D	143185.172	Zinc finger protein 536 OS=Homo sapiens OX=9606 GN=ZNF536 PE=1 SV=3
Q9BUD6	2	2	14.1701	3.58E-05	6.23E-05	21.65793	1	2D	HYS	36359.629	Spondin-2 OS=Homo sapiens OX=9606 GN=SPON2 PE=1 SV=3
O95197	4	2	25.2581	5.17E-05	8.60E-05	1.787517	1	2D	HYS	113238.4811	Reticulon-3 OS=Homo sapiens OX=9606 GN=RTN3 PE=1 SV=2
P24821;F5H7V9;H0YGZ3;J3QSU6;E	3	2	20.5631	3.23E-08	4.08E-07	5.832636	1	0.3PG	2D	246499.3625	Tenascin OS=Homo sapiens OX=9606 GN=TNC PE=1 SV=3
A0A7P0T8Y0;Q8IY21;A0A7P0TAZ7;	4	2	21.6087	2.48E-07	1.42E-06	7.016049	1	HYS	0.3PG	196337.4455	Probable ATP-dependent RNA helicase DDX60 OS=Homo sapiens OX=9606 GN=DDX60 PE=4 SV=1

O60858;X6R9U5	3	2	14.1624	6.41E-07	2.46E-06	4.928712	1	0.3PG	2D	47843.0439	E3 ubiquitin-protein ligase TRIM13 OS=Homo sapiens OX=9606 GN=TRIM13 PE=1 SV=2
G3V3X5;G3V511;Q14767;H0YJ20	3	2	25.4014	4.69E-06	1.12E-05	6.081649	1	2D	0.3PG	199068.6452	Latent-transforming growth factor beta-binding protein 2 OS=Homo sapiens OX=9606 GN=LTBP2 PE=1 SV=1
F8WD26;J3KP06;Q8WWI1;A0A0A0	4	2	25.1651	1.42E-07	1.04E-06	10.26683	1	2D	0.3PG	187376.3677	LIM domain only protein 7 OS=Homo sapiens OX=9606 GN=LMO7 PE=1 SV=2
A0A087WWA3;O60333;Q4R9M9	3	2	16.1372	5.72E-08	5.52E-07	4.397169	1	0.3PG	2D	203435.5005	Kinesin-like protein KIF1B OS=Homo sapiens OX=9606 GN=KIF1B PE=1 SV=1
A0A1W2PNV4;C9JBJ7;C9JEY1;C9JT	3	2	14.2728	6.75E-07	2.54E-06	30.13802	1	HYS	2D	77070.9711	Uncharacterized protein OS=Homo sapiens OX=9606 PE=3 SV=1
A0A499FI31;Q15020;F8VZM2;F8W	3	2	22.6546	2.83E-05	5.08E-05	2.013733	1	HYS	2D	112813.3069	Squamous cell carcinoma antigen recognized by T-cells 3 OS=Homo sapiens OX=9606 GN=SART3 PE=1 SV=1
H3BTN6;Q9H6L2;H3BMW7;H3BPY	2	2	16.5699	2.69E-07	1.44E-06	9.850746	1	HYS	2D	30872.7794	Transmembrane protein 231 OS=Homo sapiens OX=9606 GN=TMEM231 PE=1 SV=1
O95741	2	2	9.5623	8.20E-05	0.000126	10.84984	1	0.3PG	2D	62903.6988	Copine-6 OS=Homo sapiens OX=9606 GN=CPNE6 PE=1 SV=3
C9J6C5;Q9BYG3	2	2	9.2458	0.001548	0.001841	2.882684	0.996616	HYS	0.3PG	19610.7146	MKI67 FHA domain-interacting nucleolar phosphoprotein (Fragment) OS=Homo sapiens OX=9606 GN=NIFK PE=1 SV=1
A0A1C7CYX9;Q16555	2	2	14.915	0.004225	0.004846	1.530243	0.970626	2D	0.3PG	74073.3154	Dihydropyrimidinase-related protein 2 OS=Homo sapiens OX=9606 GN=DPYSL2 PE=1 SV=1
F8VNV8;P54284;F8VWK1	2	2	15.7119	1.13E-08	2.04E-07	8.795579	1	0.3PG	2D	51841.6915	Calcium channel voltage-dependent subunit beta 3 OS=Homo sapiens OX=9606 GN=CACNB3 PE=1 SV=1
Q9BRQ3	2	2	18.5452	3.56E-06	9.01E-06	3.333622	1	0.3PG	2D	32865.1135	Uridine diphosphate glucose pyrophosphatase NUDT22 OS=Homo sapiens OX=9606 GN=NUDT22 PE=1 SV=3
E7EX17;P23588;F8VP89;F8VSC7;F8	2	2	11.5104	2.37E-07	1.38E-06	4.994224	1	HYS	0.3PG	69754.83	Eukaryotic translation initiation factor 4B OS=Homo sapiens OX=9606 GN=EIF4B PE=1 SV=1
P61978;Q5T6W2	2	2	16.6076	1.34E-05	2.70E-05	3.641175	1	HYS	0.3PG	51261.4781	Heterogeneous nuclear ribonucleoprotein K OS=Homo sapiens OX=9606 GN=HNRNPK PE=1 SV=1
A2A2Z9	2	2	8.8419	6.28E-05	0.000101	2.192448	1	HYS	2D	119200.3751	Ankyrin repeat domain-containing protein 18B OS=Homo sapiens OX=9606 GN=ANKRD18B PE=1 SV=1
O60568	2	2	10.99	3.82E-05	6.60E-05	2.298181	1	0.3PG	2D	85355.5548	Multifunctional procollagen lysine hydroxylase and glycosyltransferase LH3 OS=Homo sapiens OX=9606 GN=PLOD3 PE=1 SV=1
Q9UHC7;C9IY57;C9J031;C9JYX8	2	2	9.4631	5.65E-05	9.29E-05	4.642173	1	2D	0.3PG	54718.1872	E3 ubiquitin-protein ligase makorin-1 OS=Homo sapiens OX=9606 GN=MKRN1 PE=1 SV=3
A0A7I2V4C0	2	2	9.6755	2.01E-05	3.83E-05	1.878647	1	HYS	0.3PG	10018.3043	Serine/threonine-protein kinase TBK1 (Fragment) OS=Homo sapiens OX=9606 GN=TBK1 PE=4 SV=1
Q06323	2	2	9.8323	5.25E-08	5.43E-07	11.22742	1	0.3PG	2D	28894.2293	Proteasome activator complex subunit 1 OS=Homo sapiens OX=9606 GN=PSME1 PE=1 SV=1
A0A590UK04;A0A590UKC4;A0A5F	3	2	14.6721	0.575607	0.576285	1.096856	0.111379	2D	0.3PG	113204.1409	AP-3 complex subunit beta OS=Homo sapiens OX=9606 GN=AP3B2 PE=1 SV=1
P0DPE3	3	2	14.7281	0.000271	0.000361	5.088669	0.999995	HYS	0.3PG	33748.5411	Transmembrane and death domain protein 1 OS=Homo sapiens OX=9606 GN=TMDD1 PE=3 SV=1
C9JZ99;F8WD41;Q15166	2	2	11.1465	0.002869	0.00335	4.087964	0.985851	0.3PG	HYS	27899.0633	Paraoxonase OS=Homo sapiens OX=9606 GN=PON3 PE=1 SV=1
Q8TE82	2	2	10.7316	6.49E-05	0.000103	2.269335	1	2D	0.3PG	148672.3918	SH3 domain and tetratricopeptide repeat-containing protein 1 OS=Homo sapiens OX=9606 GN=SH3TC1 PE=1 SV=3
J3QLI9;P62314	2	2	19.8941	4.10E-06	1.01E-05	3.46629	1	HYS	0.3PG	8392.7543	Small nuclear ribonucleoprotein Sm D1 OS=Homo sapiens OX=9606 GN=SNRPD1 PE=1 SV=1
A0A7I2YQF1;P42229	2	2	8.6209	1.81E-07	1.19E-06	6.262591	1	2D	0.3PG	88263.4797	Signal transducer and activator of transcription OS=Homo sapiens OX=9606 GN=STAT5A PE=1 SV=1
Q9P2R3	3	2	12.7003	4.16E-05	7.15E-05	4.59566	1	2D	0.3PG	129996.1892	Rabankyrin-5 OS=Homo sapiens OX=9606 GN=ANKFY1 PE=1 SV=2
P69905;G3V1N2	2	2	30.6114	7.54E-06	1.66E-05	2.89228	1	0.3PG	2D	15314.5997	Hemoglobin subunit alpha OS=Homo sapiens OX=9606 GN=HBA1 PE=1 SV=2
A0A3B3IUC0;Q9Y287;A0A3B3ISG3	2	2	20.2553	1.18E-06	3.81E-06	16.81809	1	2D	0.3PG	24542.1817	Integral membrane protein 2 OS=Homo sapiens OX=9606 GN=ITM2B PE=1 SV=1
P02753;Q5VY30	2	2	12.2373	2.94E-05	5.23E-05	1.822517	1	0.3PG	2D	23352.2121	Retinol-binding protein 4 OS=Homo sapiens OX=9606 GN=RBP4 PE=1 SV=3
Q3T906;F8VQW2;Q9BUA5	2	2	14.9415	1.58E-06	4.85E-06	3.209014	1	HYS	0.3PG	144876.691	N-acetylglucosamine-1-phosphotransferase subunits alpha/beta OS=Homo sapiens OX=9606 GN=GNPTAB PE=1 SV=1
P28066	2	2	16.8734	2.27E-05	4.27E-05	1.827059	1	2D	0.3PG	26582.1786	Proteasome subunit alpha type-5 OS=Homo sapiens OX=9606 GN=PSMA5 PE=1 SV=3
A0A087WUE9;M0R3C7;Q92797;M	2	2	11.5474	7.29E-07	2.68E-06	2.457136	1	0.3PG	2D	118179.9742	Symplekin OS=Homo sapiens OX=9606 GN=SYMPK PE=1 SV=1
O75582;A0A0A6YYC0;A0A3B3ISL8;	2	2	10.234	0.040901	0.042762	1.366637	0.638005	0.3PG	2D	90435.8438	Ribosomal protein S6 kinase alpha-5 OS=Homo sapiens OX=9606 GN=RPS6KA5 PE=1 SV=1
Q12907;D6RBV2	2	2	9.5696	1.88E-08	2.91E-07	829.4784	1	HYS	2D	40570.8994	Vesicular integral-membrane protein VIP36 OS=Homo sapiens OX=9606 GN=LMAN2 PE=1 SV=1
Q49AJ0	2	2	13.2802	1.31E-08	2.27E-07	33.24701	1	HYS	0.3PG	157138.593	Protein FAM135B OS=Homo sapiens OX=9606 GN=FAM135B PE=1 SV=2
Q92556	2	2	12.8867	2.06E-07	1.29E-06	25.65937	1	HYS	2D	84571.0126	Engulfment and cell motility protein 1 OS=Homo sapiens OX=9606 GN=ELMO1 PE=1 SV=2
P98077	2	2	9.0494	1.28E-05	2.60E-05	2.289444	1	2D	0.3PG	62600.4725	SHC-transforming protein 2 OS=Homo sapiens OX=9606 GN=SHC2 PE=1 SV=4
G3V3H7	2	2	14.94	1.86E-07	1.20E-06	8.602599	1	0.3PG	2D	140666.881	A-kinase anchor protein 6 OS=Homo sapiens OX=9606 GN=AKAP6 PE=1 SV=1
A0A0C4DFV9;P0DME0;Q01105	2	2	11.875	7.08E-05	0.000112	1.442149	1	HYS	0.3PG	31124.114	Protein SET OS=Homo sapiens OX=9606 GN=SET PE=1 SV=1
E7ERP7;E9PEV4;H0Y7L5;P02649	2	2	12.2391	1.73E-06	5.22E-06	5.571418	1	2D	0.3PG	25017.5163	Apolipoprotein E (Fragment) OS=Homo sapiens OX=9606 GN=APOE PE=1 SV=1
Q6NZI2	2	2	10.6067	8.34E-07	2.94E-06	5.762073	1	2D	0.3PG	43476.19	Caveolae-associated protein 1 OS=Homo sapiens OX=9606 GN=CAVIN1 PE=1 SV=1
A0A2R8Y448;A0A2R8Y4E3;A0A2R8	2	2	11.663	7.36E-05	0.000115	5.220786	1	2D	0.3PG	44367.5949	Epsilon-sarcoglycan OS=Homo sapiens OX=9606 GN=SGCE PE=1 SV=1
A0A075B6K5;P80748	2	2	5.9966	2.89E-05	5.16E-05	8.409828	1	0.3PG	2D	12445.78	Immunoglobulin lambda variable 3-9 OS=Homo sapiens OX=9606 GN=IGLV3-9 PE=3 SV=1
A0A0G2JLR5;A0A0G2JMW4;Q0283	2	2	9.9737	7.00E-05	0.000111	2.685188	1	2D	0.3PG	36887.6224	Ras association domain-containing protein 7 OS=Homo sapiens OX=9606 GN=RASSF7 PE=1 SV=1
P35219	2	2	10.0582	9.29E-09	1.93E-07	15.26371	1	0.3PG	2D	33258.3287	Carbonic anhydrase-related protein OS=Homo sapiens OX=9606 GN=CA8 PE=1 SV=3
H0Y5F3;H7C2E7	2	2	23.6092	1.30E-05	2.63E-05	2.568108	1	2D	0.3PG	25161.4512	Filamin-A (Fragment) OS=Homo sapiens OX=9606 GN=FLNA PE=1 SV=1
E5RH37;E5RJT2;E9PBN4;F8W7M8;	2	2	10.4949	9.77E-08	8.30E-07	6.300578	1	0.3PG	2D	114833.6576	Zinc finger protein ZFAT OS=Homo sapiens OX=9606 GN=ZFAT PE=1 SV=1
Q6UW02;E9PHG5	3	2	11.8348	0.00031	0.000408	1.665094	0.99999	HYS	0.3PG	52774.604	Cytochrome P450 20A1 OS=Homo sapiens OX=9606 GN=CYP20A1 PE=1 SV=1
Q9BZV1	3	2	14.5928	1.00E-06	3.44E-06	4.605919	1	0.3PG	2D	50038.9201	UBX domain-containing protein 6 OS=Homo sapiens OX=9606 GN=UBXN6 PE=1 SV=1
E7ET52;Q8WW38	2	2	8.2862	0.039102	0.040983	1.518209	0.648199	0.3PG	HYS	115358.0589	Zinc finger protein ZFPM2 OS=Homo sapiens OX=9606 GN=ZFPM2 PE=1 SV=1
B7Z6Z4;F8W1R7;G3V1V0;G8JLA2;J	3	2	26.9756	5.50E-06	1.27E-05	2.081249	1	2D	HYS	26992.0971	Myosin light polypeptide 6 OS=Homo sapiens OX=9606 GN=MYL6 PE=1 SV=1
P28070	3	2	22.135	0.019355	0.020799	1.386374	0.794275	0.3PG	HYS	29261.3518	Proteasome subunit beta type-4 OS=Homo sapiens OX=9606 GN=PSMB4 PE=1 SV=4
Q9BWV1	3	2	14.7644	0.048436	0.050454	1.668952	0.599164	0.3PG	2D	122485.3102	Brother of CDO OS=Homo sapiens OX=9606 GN=BOC PE=1 SV=1
E7ESZ3;Q9H7P9;C9J7S4;C9JVL3;M	2	2	9.5059	0.000444	0.000571	3.17506	0.999949	2D	0.3PG	134801.5433	Pleckstrin homology domain-containing family G member 2 OS=Homo sapiens OX=9606 GN=PLEKHG2 PE=1 SV=2
Q92688	3	2	20.7063	3.05E-06	8.09E-06	4.512806	1	2D	0.3PG	28958.8378	Acidic leucine-rich nuclear phosphoprotein 32 family member B OS=Homo sapiens OX=9606 GN=ANP32B PE=1 SV=1
Q6RI45	3	2	16.7842	2.32E-06	6.52E-06	6.261691	1	0.3PG	2D	205365.6546	Bromodomain and WD repeat-containing protein 3 OS=Homo sapiens OX=9606 GN=BRWD3 PE=1 SV=2
A0A494C100;Q9UJA3	3	2	13.5368	0.342782	0.346451	1.160534	0.186563	2D	HYS	113475.5866	DNA helicase OS=Homo sapiens OX=9606 PE=3 SV=1
P23378	3	2	13.7731	1.17E-06	3.80E-06	13.91702	1	0.3PG	HYS	114326.7696	Glycine dehydrogenase (decarboxylating)_ mitochondrial OS=Homo sapiens OX=9606 GN=GLDC PE=1 SV=2
P51161	2	2	10.2692	1.43E-06	4.51E-06	4.699168	1	0.3PG	HYS	14371.2849	Gastrotropin OS=Homo sapiens OX=9606 GN=FABP6 PE=1 SV=2
Q9BY76;M0QZ51;M0R0N8;M0R2X	3	2	16.0406	0.000449	0.000575	1.983525	0.999947	2D	HYS	45613.3461	Angiopoietin-related protein 4 OS=Homo sapiens OX=9606 GN=ANGPTL4 PE=1 SV=2
P55197	3	2	21.0563	5.65E-06	1.30E-05	8.602678	1	0.3PG	2D	114518.1293	Protein AF-10 OS=Homo sapiens OX=9606 GN=MLLT10 PE=1 SV=2
Q9ULJ3	3	2	23.3224	1.73E-07	1.17E-06	13.5402	1	HYS	0.3PG	120638.3743	Zinc finger and BTB domain-containing protein 21 OS=Homo sapiens OX=9606 GN=ZBTB21 PE=1 SV=2
Q15293;E9PP27	3	2	18.5284	2.46E-05	4.51E-05	2.085739	1	0.3PG	2D	38890.0441	Reticulocalbin-1 OS=Homo sapiens OX=9606 GN=RCN1 PE=1 SV=1
P61088;F8VSD4;F8VV71;F8VZ29;F8	3	2	20.3738	3.44E-06	8.80E-06	3.016525	1	HYS	0.3PG	17194.8797	Ubiquitin-conjugating enzyme E2 N OS=Homo sapiens OX=9606 GN=UBE2N PE=1 SV=1
O43272	3	2	19.2971	7.65E-05	0.000119	2.075139	1	0.3PG	2D	68572.4638	Proline dehydrogenase 1_ mitochondrial OS=Homo sapiens OX=9606 GN=PRODH PE=1 SV=3
A0A2R8Y212;A0A2R8Y445;A0A2R8	2	2	16.5948	1.39E-05	2.78E-05	6.161384	1	0.3PG	2D	219729.9551	DNA helicase OS=Homo sapiens OX=9606 GN=CHD4 PE=1 SV=1
Q99541	3	2	23.5966	0.038582	0.040488	1.245738	0.651219	2D	HYS	48303.6235	Perilipin-2 OS=Homo sapiens OX=9606 GN=PLIN2 PE=1 SV=2
P19224	3	2	13.2557	2.35E-05	4.37E-05	2.487264	1	2D	0.3PG	61378.1486	UDP-glucuronosyltransferase 1-6 OS=Homo sapiens OX=9606 GN=UGT1A6 PE=1 SV=2
Q7L9B9	3	2	19.2194	1.92E-06	5.64E-06	4.286064	1	0.3PG	2D	62858.9318	Endonuclease/exonuclease/phosphatase family domain-containing protein 1 OS=Homo sapiens OX=9606 GN=EEPD1 PE=1 SV=2
B5MCZ9;Q16769	3	2	9.8158	4.48E-06	1.08E-05	4.724463	1	HYS	0.3PG	32221.9305	Glutaminyl-peptide cyclotransferase (Fragment) OS=Homo sapiens OX=9606 GN=QPCT PE=1 SV=1
A0A2R8YEC9;E9PFW2;O00462;A0A	2	2	9.9437	0.076999	0.079622	1.242607	0.49092	2D	HYS	107077.9186	Beta-mannosidase OS=Homo sapiens OX=9606 GN=MANBA PE=1 SV=1
F8WCK4	2	2	8.883	0.072784	0.075355	1.595468	0.504044	2D	HYS	28733.4839	Glycine--tRNA ligase OS=Homo sapiens OX=9606 GN=GARS1 PE=1 SV=2

Q96PB1;F8WDQ7;F8WDW8	3	2	17.5105	6.01E-06	1.37E-05	4.079939	1	HYS	0.3PG	92991.5723	N-acetylneuraminate 9-O-acetyltransferase OS=Homo sapiens OX=9606 GN=CASD1 PE=1 SV=1
H0YN26;P39687;H7BZ09	3	2	28.7304	0.006238	0.007032	2.104934	0.945068	2D	0.3PG	20111.3727	Acidic leucine-rich nuclear phosphoprotein 32 family member A OS=Homo sapiens OX=9606 GN=ANP32A PE=1 SV=1
H3BSS6;Q9H2Y7;A0A0C4DGM5;H3	3	2	13.7337	9.75E-06	2.05E-05	2.985091	1	HYS	0.3PG	213943.6681	Zinc finger protein 106 OS=Homo sapiens OX=9606 GN=ZNF106 PE=1 SV=2
A0A2R8Y2X6;A1A5D9	2	2	9.0096	0.000145	0.000208	2.646156	1	2D	0.3PG	60831.7068	BICD family-like cargo adapter 2 OS=Homo sapiens OX=9606 GN=BICDL2 PE=1 SV=1
P54257	3	2	14.3114	0.362306	0.364882	1.146021	0.177688	2D	0.3PG	76247.9676	Huntingtin-associated protein 1 OS=Homo sapiens OX=9606 GN=HAP1 PE=1 SV=3
A0A075B7B8	3	2	31.8821	0.357138	0.360531	1.149745	0.179971	0.3PG	HYS	13045.6695	Immunoglobulin heavy variable 3/OR16-12 (non-functional) (Fragment) OS=Homo sapiens OX=9606 GN=IGHV3OR16-12 PE=1 SV=1
Q24JP5;H0YFB3	2	2	11.1393	0.000592	0.000744	1.842708	0.99984	2D	0.3PG	111079.4602	Transmembrane protein 132A OS=Homo sapiens OX=9606 GN=TMEM132A PE=1 SV=1
Q96DN5;E7ERK7;E7EWW7	3	2	10.5632	2.65E-05	4.80E-05	5.762899	1	0.3PG	2D	125216.1069	TBC1 domain family member 31 OS=Homo sapiens OX=9606 GN=TBC1D31 PE=1 SV=2
Q9UBG0	2	2	8.8159	5.44E-06	1.26E-05	31.80029	1	2D	0.3PG	169868.183	C-type mannose receptor 2 OS=Homo sapiens OX=9606 GN=MRC2 PE=1 SV=2
A8MX75;P18074;E7EVE9	2	2	9.4473	8.54E-05	0.000131	1.888741	1	0.3PG	2D	81310.4419	General transcription and DNA repair factor IIH helicase subunit XPD (Fragment) OS=Homo sapiens OX=9606 GN=ERCC2 PE=1 SV=2
A0A087WV29;Q9H0A0	2	2	15.8333	0.36127	0.364269	1.211076	0.178142	2D	HYS	94276.0663	RNA cytidine acetyltransferase OS=Homo sapiens OX=9606 GN=NAT10 PE=1 SV=1
Q96C11;A0A0U1RRB9;F2Z2V1;A0A	3	2	15.4022	5.87E-06	1.34E-05	3.162939	1	HYS	0.3PG	60677.5212	FGGY carbohydrate kinase domain-containing protein OS=Homo sapiens OX=9606 GN=FGGY PE=1 SV=2
O94830	2	2	13.7264	0.008569	0.009484	1.797121	0.91482	2D	0.3PG	81716.2579	Phospholipase DDHD2 OS=Homo sapiens OX=9606 GN=DDHD2 PE=1 SV=2
Q5TF85;Q96IP4	2	2	9.5172	4.02E-07	1.86E-06	8.178669	1	0.3PG	2D	60032.5064	Family with sequence similarity 46_ member A_ isoform CRA_a OS=Homo sapiens OX=9606 GN=TENT5A PE=1 SV=1
Q7Z3V4;F5H5T5	3	2	19.3558	0.00041	0.00053	2.312967	0.999964	HYS	0.3PG	124580.9538	Ubiquitin-protein ligase E3B OS=Homo sapiens OX=9606 GN=UBE3B PE=1 SV=3
Q9H0Q0;C9IYV6;C9JPE5	3	2	10.0913	0.017136	0.018485	2.303606	0.816095	0.3PG	2D	37712.1419	CYFIP-related Rac1 interactor A OS=Homo sapiens OX=9606 GN=CYRIA PE=2 SV=1
A0A182DWF2;A0A182DWF3;D3YTF	2	2	14.433	8.09E-05	0.000125	2.844033	1	2D	0.3PG	54308.6826	Thioredoxin-disulfide reductase OS=Homo sapiens OX=9606 GN=TXNRD2 PE=1 SV=1
A6NGS2	2	2	10.1099	0.000101	0.000152	3.379905	1	HYS	0.3PG	14648.4062	Glutamate-rich protein 4 OS=Homo sapiens OX=9606 GN=ERICH4 PE=1 SV=2
P38398;E7EUM2;Q5YLB2;A0A0U1R	3	2	15.0531	0.001454	0.001733	4.930923	0.997137	HYS	0.3PG	210230.3196	Breast cancer type 1 susceptibility protein OS=Homo sapiens OX=9606 GN=BRCA1 PE=1 SV=2
Q99497;K7ELW0;K7EN27	4	2	48.98	0.001123	0.00136	1.489706	0.998617	HYS	0.3PG	20062.173	Parkinson disease protein 7 OS=Homo sapiens OX=9606 GN=PARK7 PE=1 SV=2
Q52LR7;C9J1X4;E7ETK1	4	2	17.8352	5.94E-05	9.63E-05	4.05296	1	HYS	0.3PG	91950.4263	Enhancer of polycomb homolog 2 OS=Homo sapiens OX=9606 GN=EPC2 PE=1 SV=2
P09960;B4DEH5	8	2	50.3948	0.000195	0.000268	2.014744	0.999999	HYS	0.3PG	69912.6656	Leukotriene A-4 hydrolase OS=Homo sapiens OX=9606 GN=LTA4H PE=1 SV=2
Q8TDY2;E5RH44	6	2	31.7581	0.004816	0.005488	142.2056	0.963323	0.3PG	HYS	185201.194	RB1-inducible coiled-coil protein 1 OS=Homo sapiens OX=9606 GN=RB1CC1 PE=1 SV=3
P04259;F8W0C6	15	2	115.7668	7.34E-08	6.71E-07	21.63233	1	HYS	2D	60352.171	Keratin_ type II cytoskeletal 6B OS=Homo sapiens OX=9606 GN=KRT6B PE=1 SV=5
Q8IZ41	6	2	27.4009	4.37E-08	4.95E-07	4.711513	1	0.3PG	2D	83620.679	Ras and EF-hand domain-containing protein OS=Homo sapiens OX=9606 GN=RASEF PE=1 SV=1
Q8NI08	5	2	28.0146	0.003408	0.003926	1.722475	0.980135	2D	HYS	106788.9704	Nuclear receptor coactivator 7 OS=Homo sapiens OX=9606 GN=NCOA7 PE=1 SV=2
Q9P2N2;E9PMX7;J3QKM0	5	2	29.2803	4.15E-05	7.15E-05	2.791703	1	2D	HYS	82458.9674	Rho GTPase-activating protein 28 OS=Homo sapiens OX=9606 GN=ARHGAP28 PE=1 SV=3
J3KR69;Q9H892	5	2	27.8213	0.000145	0.000208	2.307134	1	0.3PG	2D	80624.4985	Tetratricopeptide repeat domain 12_ isoform CRA_d OS=Homo sapiens OX=9606 GN=TTC12 PE=1 SV=1
P0CG39;P0CG38	13	2	102.9712	4.35E-07	1.92E-06	2.518206	1	2D	0.3PG	118815.9034	POTE ankyrin domain family member J OS=Homo sapiens OX=9606 GN=POTEJ PE=3 SV=1
E7ES21	6	2	43.42	4.44E-08	4.97E-07	13.28032	1	0.3PG	2D	110451.4742	Hephaestin OS=Homo sapiens OX=9606 GN=HEPH PE=1 SV=1
Q14112;A0A087WZP6;H0YJV3	5	2	24.2892	6.67E-05	0.000106	1.638894	1	2D	0.3PG	154048.2473	Nidogen-2 OS=Homo sapiens OX=9606 GN=NID2 PE=1 SV=3
O14802	5	2	20.169	2.10E-06	6.04E-06	3.443271	1	HYS	2D	157637.7587	DNA-directed RNA polymerase III subunit RPC1 OS=Homo sapiens OX=9606 GN=POLR3A PE=1 SV=2
Q16352	6	2	54.5649	0.000654	0.00082	2.284915	0.999768	2D	HYS	55561.7724	Alpha-internexin OS=Homo sapiens OX=9606 GN=INA PE=1 SV=2
J3KR97;Q9BTW9;I3L163;I3L439;I3L	5	2	21.8981	0.000691	0.000854	1.682468	0.999716	HYS	0.3PG	138433.6161	Tubulin-specific chaperone D OS=Homo sapiens OX=9606 GN=TBCD PE=1 SV=1
A0A3B3ITQ1	5	2	20.5287	4.83E-06	1.14E-05	222.899	1	HYS	2D	231905.6526	Inositol 1_4_5-trisphosphate receptor type 1 (Fragment) OS=Homo sapiens OX=9606 GN=ITPR1 PE=1 SV=1
A0A0G2JP53;A0A0G2JR31;A0A0G2	4	2	17.8857	0.020053	0.021494	1.339554	0.787701	2D	HYS	104632.6166	Thyroid peroxidase OS=Homo sapiens OX=9606 GN=TPO PE=1 SV=1
P35241;A0A2R8Y5S7;A0A2R8Y7M3	11	2	73.5169	1.09E-05	2.27E-05	5.090022	1	2D	0.3PG	68678.0722	Radixin OS=Homo sapiens OX=9606 GN=RDX PE=1 SV=1
P12814;G3V2N5;G3V2W4;H0YJW3	40	2	386.7419	0.004371	0.005008	1.336504	0.968841	HYS	2D	103628.0313	Alpha-actinin-1 OS=Homo sapiens OX=9606 GN=ACTN1 PE=1 SV=2
Q86YA3;G3XAL8	4	2	19.5885	0.135375	0.138637	1.311264	0.363552	HYS	0.3PG	239054.1609	Protein ZGRF1 OS=Homo sapiens OX=9606 GN=ZGRF1 PE=1 SV=3
E7ESC6;Q9UIA9	4	2	18.217	0.032141	0.033854	1.294622	0.691713	2D	HYS	125435.5196	Exportin-7 OS=Homo sapiens OX=9606 GN=XPO7 PE=1 SV=1
Q04917;A2IDB2	4	2	62.2195	8.70E-05	0.000133	1.954181	1	2D	HYS	28389.8608	14-3-3 protein eta OS=Homo sapiens OX=9606 GN=YWHAH PE=1 SV=4
H0Y300	21	2	270.197	0.0272	0.028792	1.306044	0.727291	HYS	2D	49847.3781	Haptoglobin OS=Homo sapiens OX=9606 GN=HP PE=1 SV=4
P02533;A0A3B3IS58	10	2	73.6735	4.20E-06	1.02E-05	3.882994	1	0.3PG	2D	51903.7472	Keratin_ type I cytoskeletal 14 OS=Homo sapiens OX=9606 GN=KRT14 PE=1 SV=4
Q13822;E7EUF1;E5RIA2;E5RJ49;E5I	5	2	20.2671	6.15E-05	9.93E-05	2.78394	1	2D	0.3PG	100875.6413	Ectonucleotide pyrophosphatase/phosphodiesterase family member 2 OS=Homo sapiens OX=9606 GN=ENPP2 PE=1 SV=3
Q9ULH0;A0A1W2PPB7;E9PH70;H0Y	6	2	30.9288	0.000161	0.000226	2.906369	1	0.3PG	HYS	198139.2516	Kinase D-interacting substrate of 220 kDa OS=Homo sapiens OX=9606 GN=KIDINS220 PE=1 SV=3
Q16363;A0A494C139;A0A494C1K8	46	2	387.471	9.33E-05	0.000141	3.514057	1	2D	HYS	205147.7618	Laminin subunit alpha-4 OS=Homo sapiens OX=9606 GN=LAMA4 PE=1 SV=4
Q15811;A8CTZ0;F8W7U0;C9JQZ7	5	2	30.8024	7.69E-07	2.77E-06	5.025089	1	HYS	0.3PG	196277.3914	Intersectin-1 OS=Homo sapiens OX=9606 GN=ITSN1 PE=1 SV=3
Q8NFY4;H0YMP7	5	2	31.2615	4.36E-09	1.32E-07	199.7915	1	0.3PG	2D	121127.0428	Semaphorin-6D OS=Homo sapiens OX=9606 GN=SEMA6D PE=1 SV=1
A0A494C0N4;A0A7P0T8P4;A0A7P0	5	2	25.8781	0.002807	0.003282	1.615751	0.986474	HYS	0.3PG	147545.1981	Tetratricopeptide repeat protein 21B OS=Homo sapiens OX=9606 GN=TTC21B PE=1 SV=1
P30086	5	2	30.2407	0.00011	0.000162	1.271981	1	HYS	2D	21170.8601	Phosphatidylethanolamine-binding protein 1 OS=Homo sapiens OX=9606 GN=PEBP1 PE=1 SV=3
P52209;K7ELN9;K7EM49;K7EMN2;	6	2	48.1797	0.000152	0.000216	2.155261	1	HYS	0.3PG	53653.3398	6-phosphogluconate dehydrogenase_ decarboxylating OS=Homo sapiens OX=9606 GN=PGD PE=1 SV=3
P23468;F5GWR7;Q3KPI9;C9J8S8	6	2	29.7324	7.96E-06	1.73E-05	117.2101	1	2D	HYS	215786.7785	Receptor-type tyrosine-protein phosphatase delta OS=Homo sapiens OX=9606 GN=PTPRD PE=1 SV=2
B4DPQ0;A0A3B3ISR2;F5H2D0;A0A	26	2	229.1752	2.62E-08	3.54E-07	6.038802	1	2D	0.3PG	83429.3834	Complement subcomponent C1r OS=Homo sapiens OX=9606 GN=C1R PE=1 SV=1
Q5VTE0;A0A087WVQ9;A0A7I2V3H	6	2	46.488	2.46E-08	3.42E-07	10.28392	1	2D	0.3PG	50527.2837	Putative elongation factor 1-alpha-like 3 OS=Homo sapiens OX=9606 GN=EEF1A1P5 PE=5 SV=1
P32119;A6NIW5	6	2	50.7046	0.059925	0.062269	1.226671	0.549532	0.3PG	2D	22063.0168	Peroxiredoxin-2 OS=Homo sapiens OX=9606 GN=PRDX2 PE=1 SV=5
A0A2R8Y7R2;P68871;F8W6P5;A0A	6	2	67.1545	7.67E-06	1.68E-05	4.75058	1	0.3PG	2D	12232.9171	Hemoglobin subunit beta OS=Homo sapiens OX=9606 GN=HBB PE=1 SV=1
Q6WCQ1	6	2	25.3702	2.73E-07	1.44E-06	2.752629	1	2D	0.3PG	117331.7139	Myosin phosphatase Rho-interacting protein OS=Homo sapiens OX=9606 GN=MPRIP PE=1 SV=3
Q6VMQ6;A8MV73	5	2	23.2912	0.000753	0.000925	2.267351	0.999617	2D	0.3PG	137249.2518	Activating transcription factor 7-interacting protein 1 OS=Homo sapiens OX=9606 GN=ATF7IP PE=1 SV=3
Q7Z3Y8	5	2	42.2934	2.87E-05	5.15E-05	52.81455	1	0.3PG	HYS	50449.7064	Keratin_ type I cytoskeletal 27 OS=Homo sapiens OX=9606 GN=KRT27 PE=1 SV=2
A0A6I8PIV1;A0A6I8PL42;A0A6I8PLI	6	2	39.9287	7.63E-08	6.81E-07	15.4122	1	HYS	0.3PG	135535.8323	Leucyl-tRNA synthetase OS=Homo sapiens OX=9606 GN=LARS1 PE=1 SV=1
P35609;A0A494C060	22	2	175.2508	1.00E-06	3.44E-06	257.0625	1	0.3PG	HYS	104424.2806	Alpha-actinin-2 OS=Homo sapiens OX=9606 GN=ACTN2 PE=1 SV=1
A0A024R6I7;A0A0G2JRN3	21	2	293.3845	7.63E-06	1.68E-05	11.68877	1	0.3PG	2D	46879.6598	Alpha-1-antitrypsin OS=Homo sapiens OX=9606 GN=SERPINA1 PE=1 SV=1
P02458	5	2	31.899	6.10E-07	2.39E-06	41.82637	1	2D	0.3PG	142869.0714	Collagen alpha-1(II) chain OS=Homo sapiens OX=9606 GN=COL2A1 PE=1 SV=3
H3BU85	6	2	40.4476	3.43E-06	8.80E-06	4.088349	1	2D	0.3PG	36481.9997	Beta-N-acetylhexosaminidase (Fragment) OS=Homo sapiens OX=9606 GN=HEXA PE=1 SV=1
P63151;A0A590UJJ1;A0A590UJY3;E	5	2	23.2239	0.012894	0.013979	1.103677	0.862041	HYS	2D	52205.4115	Serine/threonine-protein phosphatase 2A 55 kDa regulatory subunit B alpha isoform OS=Homo sapiens OX=9606 GN=PPP2R2A PE=1 SV=1
P27348	4	2	65.9579	4.76E-06	1.13E-05	3.098086	1	2D	0.3PG	28049.4331	14-3-3 protein theta OS=Homo sapiens OX=9606 GN=YWHAQ PE=1 SV=1
P0C0L5;A0A0G2JL54;A0A140TA29;	40	2	339.7792	0.063022	0.065407	1.245788	0.537734	0.3PG	HYS	194291.4576	Complement C4-B OS=Homo sapiens OX=9606 GN=C4B PE=1 SV=2
Q9Y623	9	2	50.354	0.00013	0.000189	2.155784	1	0.3PG	2D	224041.3084	Myosin-4 OS=Homo sapiens OX=9606 GN=MYH4 PE=2 SV=2
O75145;R4GN36	4	2	22.7702	0.502784	0.50397	1.072756	0.129309	0.3PG	HYS	134066.8679	Liprin-alpha-3 OS=Homo sapiens OX=9606 GN=PPFIA3 PE=1 SV=3
P08567	4	2	21.6068	3.31E-05	5.81E-05	3.257359	1	2D	HYS	40524.1162	Pleckstrin OS=Homo sapiens OX=9606 GN=PLEK PE=1 SV=3
O14818;A0A087WYS6;Q8TAA3	4	2	21.8232	2.42E-05	4.44E-05	2.010877	1	2D	0.3PG	28057.9601	Proteasome subunit alpha type-7 OS=Homo sapiens OX=9606 GN=PSMA7 PE=1 SV=1
Q15323	4	2	22.7666	0.011418	0.012474	1.908063	0.879403	2D	0.3PG	48662.7711	Keratin_ type I cuticular Ha1 OS=Homo sapiens OX=9606 GN=KRT31 PE=1 SV=3

A0A087WYS1;A0A7I2YQ74;E7EUC7	4	2	26.9426	4.88E-06	1.15E-05	2.664427	1	HYS	0.3PG	52248.7414	UTP--glucose-1-phosphate uridylyltransferase OS=Homo sapiens OX=9606 GN=UGP2 PE=1 SV=2
F8VZY9;P05783	5	2	39.0673	4.48E-05	7.57E-05	2.461013	1	0.3PG	2D	43774.1421	Keratin_ type I cytoskeletal 18 OS=Homo sapiens OX=9606 GN=KRT18 PE=1 SV=1
Q14568	7	2	38.1165	0.015451	0.016687	1.229318	0.833661	HYS	0.3PG	39478.9154	Heat shock protein HSP 90-alpha A2 OS=Homo sapiens OX=9606 GN=HSP90AA2P PE=1 SV=2
Q9BYG0	4	2	19.7765	0.00026	0.000348	2.619814	0.999996	2D	0.3PG	44508.9845	Lactosylceramide 1_3-N-acetyl-beta-D-glucosaminyltransferase OS=Homo sapiens OX=9606 GN=B3GNT5 PE=1 SV=1
P01877;A0A286YEY5	8	2	56.5014	5.47E-05	9.02E-05	5.736445	1	0.3PG	2D	37389.7647	Immunoglobulin heavy constant alpha 2 OS=Homo sapiens OX=9606 GN=IGHA2 PE=1 SV=4
Q9NSB2;A0A087X106;F5GYI5;O437	4	2	33.6119	5.09E-07	2.09E-06	18.16506	1	0.3PG	HYS	65983.0317	Keratin_ type II cuticular Hb4 OS=Homo sapiens OX=9606 GN=KRT84 PE=2 SV=2
Q86TJ2	4	2	18.434	0.000299	0.000395	3.757768	0.999992	2D	0.3PG	49040.6876	Transcriptional adapter 2-beta OS=Homo sapiens OX=9606 GN=TADA2B PE=1 SV=2
P62166	4	2	22.043	4.17E-05	7.15E-05	6.605871	1	0.3PG	HYS	21935.7851	Neuronal calcium sensor 1 OS=Homo sapiens OX=9606 GN=NCS1 PE=1 SV=2
M0QZQ3	4	2	21.7198	0.000939	0.001141	1.344566	0.999203	2D	HYS	226631.1773	Spectrin beta chain OS=Homo sapiens OX=9606 GN=SPTBN4 PE=1 SV=1
O43866	4	2	21.7398	6.72E-07	2.54E-06	6.539536	1	0.3PG	HYS	39627.5677	CD5 antigen-like OS=Homo sapiens OX=9606 GN=CD5L PE=1 SV=1
P15538;Q4VAR0	9	2	43.2796	3.56E-05	6.20E-05	3.499351	1	0.3PG	2D	57915.2461	Cytochrome P450 11B1_ mitochondrial OS=Homo sapiens OX=9606 GN=CYP11B1 PE=1 SV=5
Q14118;C9J196;C9JEN1;C9JQL4;C9	2	1	12.7441	0.000103	0.000153	17.47768	1	2D	0.3PG	97783.8038	Dystroglycan OS=Homo sapiens OX=9606 GN=DAG1 PE=1 SV=2
I3L2R6;Q9BRA2	2	1	16.0948	4.38E-05	7.47E-05	2.256968	1	HYS	2D	11462.9125	Thioredoxin domain-containing protein 17 OS=Homo sapiens OX=9606 GN=TXNDC17 PE=1 SV=1
H0Y7F7	2	1	9.3047	0.650815	0.650815	1.231526	0.096115	2D	0.3PG	148195.8896	Centrosome-associated protein 350 (Fragment) OS=Homo sapiens OX=9606 GN=CEP350 PE=1 SV=1
A0A1W2PRW1	2	1	10.0786	0.000545	0.00069	2.873182	0.999883	2D	0.3PG	36103.6443	N-acetyllactosaminide beta-1_6-N-acetylglucosaminyl-transferase OS=Homo sapiens OX=9606 GN=GCNT2 PE=1 SV=1
A0A0A6YYL1;A0A0U1RQM3;P4991	2	1	10.0789	3.21E-06	8.38E-06	8.010189	1	2D	HYS	20690.5422	5-formyltetrahydrofolate cyclo-ligase OS=Homo sapiens OX=9606 GN=ST20-MTHFS PE=3 SV=1
Q6S8J3	22	1	216.1701	2.29E-05	4.29E-05	47.09741	1	0.3PG	2D	122960.5057	POTE ankyrin domain family member E OS=Homo sapiens OX=9606 GN=POTEE PE=2 SV=3
A0A494C1A0	20	1	181.5114	2.09E-06	6.04E-06	4.212858	1	0.3PG	2D	100254.6686	Alpha-actinin-2 (Fragment) OS=Homo sapiens OX=9606 GN=ACTN2 PE=1 SV=1
P68036	2	1	11.991	1.96E-05	3.75E-05	2.480886	1	HYS	0.3PG	18032.6876	Ubiquitin-conjugating enzyme E2 L3 OS=Homo sapiens OX=9606 GN=UBE2L3 PE=1 SV=1
P42285;D6REC7	2	1	14.8966	0.000879	0.001075	1.910212	0.999355	HYS	0.3PG	118831.4793	Exosome RNA helicase MTR4 OS=Homo sapiens OX=9606 GN=MTREX PE=1 SV=3
Q9HBV1	2	1	9.9836	6.05E-06	1.38E-05	3.106131	1	2D	0.3PG	34269.4694	Popeye domain-containing protein 3 OS=Homo sapiens OX=9606 GN=POPDC3 PE=1 SV=2
P04433;A0A0A0MRZ8	2	1	15.4714	0.000275	0.000366	2.480962	0.999995	0.3PG	2D	12689.2465	Immunoglobulin kappa variable 3-11 OS=Homo sapiens OX=9606 GN=IGKV3-11 PE=1 SV=1
A0A7P0T963;Q8N7X0	2	1	13.5129	5.79E-05	9.45E-05	6.536987	1	2D	0.3PG	190782.8479	Androglobin OS=Homo sapiens OX=9606 GN=ADGB PE=4 SV=1
P00736	25	1	213.4793	0.02276	0.024304	28.67437	0.763404	2D	HYS	81658.4083	Complement C1r subcomponent OS=Homo sapiens OX=9606 GN=C1R PE=1 SV=2
E9PB61;Q86V81	2	1	22.9562	0.001423	0.001704	1.918016	0.997295	2D	0.3PG	27557.7053	THO complex subunit 4 OS=Homo sapiens OX=9606 GN=ALYREF PE=1 SV=1
E9PKG0	2	1	11.0081	0.000977	0.001186	307.939	0.999097	2D	0.3PG	22576.9937	Plectin (Fragment) OS=Homo sapiens OX=9606 GN=PLEC PE=1 SV=1
A0A1B0GVI3	29	1	332.1437	0.051967	0.054066	75.19743	0.582816	2D	0.3PG	63574.3405	Keratin_ type I cytoskeletal 10 OS=Homo sapiens OX=9606 GN=KRT10 PE=1 SV=2
P13645;K7EMD9;O76014;O76015	29	1	320.7782	0.000507	0.000644	2.414312	0.999913	0.3PG	2D	59055.2696	Keratin_ type I cytoskeletal 10 OS=Homo sapiens OX=9606 GN=KRT10 PE=1 SV=6
Q8WX93	2	1	12.5176	0.001172	0.001415	2.333882	0.998434	HYS	2D	151932.6987	Palladin OS=Homo sapiens OX=9606 GN=PALLD PE=1 SV=3
A0A1B0GVL3;A0A6Q8PHE5;Q0508	2	1	11.1102	0.19524	0.19851	Infinity	0.287832	0.3PG	2D	94399.9253	Ubiquitin-protein ligase E3A OS=Homo sapiens OX=9606 GN=UBE3A PE=1 SV=1
P47929	2	1	18.51	1.37E-05	2.76E-05	2.859081	1	0.3PG	2D	15132.0942	Galectin-7 OS=Homo sapiens OX=9606 GN=LGALS7 PE=1 SV=2
P54652	8	1	73.8701	0.002034	0.002395	45.51297	0.99334	2D	HYS	70306.2015	Heat shock-related 70 kDa protein 2 OS=Homo sapiens OX=9606 GN=HSPA2 PE=1 SV=1
Q5HYN5;Q8NHU0	2	1	10.1544	1.79E-06	5.37E-06	20.85722	1	0.3PG	2D	21500.8469	Cancer/testis antigen family 45 member A1 OS=Homo sapiens OX=9606 GN=CT45A1 PE=1 SV=1
P0C0L4	38	1	326.7977	2.26E-07	1.37E-06	6.529335	1	0.3PG	HYS	194382.5114	Complement C4-A OS=Homo sapiens OX=9606 GN=C4A PE=1 SV=2
C9J2C0;Q9NY65;A0A7P0T945;P683	7	1	44.4361	0.004007	0.004602	3.149799	0.973246	2D	HYS	52783.3847	Tubulin alpha chain (Fragment) OS=Homo sapiens OX=9606 GN=TUBA8 PE=1 SV=1
Q8IXI1	2	1	13.5786	2.41E-06	6.74E-06	5.725382	1	0.3PG	2D	69144.3877	Mitochondrial Rho GTPase 2 OS=Homo sapiens OX=9606 GN=RHOT2 PE=1 SV=2
Q7Z4H8;H0YEM3	4	1	27.7053	0.000906	0.001105	28.76864	0.999288	0.3PG	HYS	58971.7856	Protein O-glucosyltransferase 3 OS=Homo sapiens OX=9606 GN=POGLUT3 PE=1 SV=2
Q8WVD5	2	1	9.5398	1.83E-05	3.51E-05	2.75566	1	0.3PG	2D	26105.2269	RING finger protein 141 OS=Homo sapiens OX=9606 GN=RNF141 PE=1 SV=1
A0A0B4J2F2;P57059	2	1	12.0054	9.28E-08	7.97E-07	13.48747	1	0.3PG	HYS	86013.8001	Probable serine/threonine-protein kinase SIK1B OS=Homo sapiens OX=9606 GN=SIK1B PE=1 SV=1
Q13882	2	1	16.0865	1.46E-05	2.89E-05	20.94432	1	2D	HYS	52404.5228	Protein-tyrosine kinase 6 OS=Homo sapiens OX=9606 GN=PTK6 PE=1 SV=1
P54802	2	1	10.5203	0.000304	0.0004	4.629268	0.999991	2D	HYS	82722.0227	Alpha-N-acetylglucosaminidase OS=Homo sapiens OX=9606 GN=NAGLU PE=1 SV=2
Q99814	2	1	12.8714	5.67E-08	5.52E-07	6.686474	1	0.3PG	HYS	97713.9461	Endothelial PAS domain-containing protein 1 OS=Homo sapiens OX=9606 GN=EPAS1 PE=1 SV=3
A0A1B0GVW8	10	1	63.4073	7.04E-05	0.000111	5.541801	1	HYS	0.3PG	225670.8929	Protein unc-13 homolog B OS=Homo sapiens OX=9606 GN=UNC13B PE=1 SV=1
B1AM27;O14795;F8W8M9;I6L9J0;	10	1	64.1891	0.000122	0.000178	7.132788	1	0.3PG	2D	186514.0754	Protein unc-13 homolog B OS=Homo sapiens OX=9606 GN=UNC13B PE=1 SV=1
P39060;H7BXV5;H7C457	2	1	12.2611	0.000245	0.00033	12.88775	0.999997	2D	HYS	179499.4612	Collagen alpha-1(XVIII) chain OS=Homo sapiens OX=9606 GN=COL18A1 PE=1 SV=5
A0A087X1Z3;Q9UL46;H0YM70	2	1	10.1887	0.000162	0.000227	3.277615	1	2D	0.3PG	29297.8241	Proteasome activator complex subunit 2 OS=Homo sapiens OX=9606 GN=PSME2 PE=1 SV=1
A0A087X0M8;C9J905;C9JH37;C9JW	2	1	13.8098	8.50E-06	1.84E-05	2.891519	1	HYS	0.3PG	131878.2512	Neural cell adhesion molecule L1-like protein OS=Homo sapiens OX=9606 GN=CHL1 PE=1 SV=1
P01593;P01594	2	1	14.447	0.001361	0.001634	1.950594	0.997604	HYS	0.3PG	13019.5775	Immunoglobulin kappa variable 1D-33 OS=Homo sapiens OX=9606 GN=IGKV1D-33 PE=1 SV=2
Q9H511	2	1	4.5105	0.063456	0.065778	1.325704	0.536124	2D	HYS	71329.7415	Kelch-like protein 31 OS=Homo sapiens OX=9606 GN=KLHL31 PE=2 SV=1
Q8NI22	2	1	11.1401	3.20E-05	5.64E-05	3.14401	1	2D	0.3PG	16504.4216	Multiple coagulation factor deficiency protein 2 OS=Homo sapiens OX=9606 GN=MCFD2 PE=1 SV=1
Q7Z7G0	10	1	87.9195	5.67E-05	9.30E-05	2.341726	1	HYS	0.3PG	119326.384	Target of Nesh-SH3 OS=Homo sapiens OX=9606 GN=ABI3BP PE=1 SV=1
A0A0B4J1V1	2	1	20.6984	0.008906	0.009818	1.303656	0.910516	HYS	0.3PG	13010.7114	Immunoglobulin heavy variable 3-21 OS=Homo sapiens OX=9606 GN=IGHV3-21 PE=1 SV=1
Q9Y5S1	2	1	12.0421	0.003231	0.003732	3.505328	0.982075	HYS	0.3PG	86893.7205	Transient receptor potential cation channel subfamily V member 2 OS=Homo sapiens OX=9606 GN=TRPV2 PE=1 SV=1
Q05932	2	1	8.5628	3.45E-06	8.81E-06	4.444594	1	HYS	2D	65407.5588	Folylpolyglutamate synthase_ mitochondrial OS=Homo sapiens OX=9606 GN=FPGS PE=1 SV=3
Q96I63;Q96TA2	2	1	15.1779	5.82E-09	1.60E-07	9.741944	1	2D	HYS	82951.1443	ATP-dependent zinc metalloprotease YME1L1 OS=Homo sapiens OX=9606 GN=YME1L1 PE=1 SV=1
A0A0J9YY99;P01764;P01768;P0DP	2	1	26.5988	0.001378	0.001652	9.713354	0.997522	0.3PG	2D	13135.8932	Ig-like domain-containing protein (Fragment) OS=Homo sapiens OX=9606 PE=1 SV=1
Q9UJ98;D6W5U7	2	1	9.6493	9.96E-05	0.000149	10.46356	1	0.3PG	2D	140345.4658	Cohesin subunit SA-3 OS=Homo sapiens OX=9606 GN=STAG3 PE=1 SV=2
P51575	2	1	14.975	6.81E-06	1.52E-05	3.840824	1	HYS	0.3PG	45607.4374	P2X purinoceptor 1 OS=Homo sapiens OX=9606 GN=P2RX1 PE=1 SV=1
F5GY03;E5RK62	9	1	170.6996	4.28E-07	1.91E-06	15.14206	1	2D	0.3PG	17926.2208	Osteonectin (Fragment) OS=Homo sapiens OX=9606 GN=SPARC PE=1 SV=1
Q12769	2	1	12.4991	0.000395	0.000512	2.784094	0.99997	2D	0.3PG	164459.7337	Nuclear pore complex protein Nup160 OS=Homo sapiens OX=9606 GN=NUP160 PE=1 SV=3
Q9Y6Y9	2	1	10.3605	1.85E-06	5.47E-06	8.033431	1	0.3PG	2D	18944.7658	Lymphocyte antigen 96 OS=Homo sapiens OX=9606 GN=LY96 PE=1 SV=2
P19012;A8MT21;C9JTG5;A0A3B3IT	9	1	59.9108	0.000191	0.000264	53.84177	0.999999	0.3PG	HYS	49439.9298	Keratin_ type I cytoskeletal 15 OS=Homo sapiens OX=9606 GN=KRT15 PE=1 SV=3
Q9NQW1;E9PKR7	2	1	10.17	1.61E-06	4.91E-06	42.33377	1	2D	0.3PG	130065.8667	Protein transport protein Sec31B OS=Homo sapiens OX=9606 GN=SEC31B PE=1 SV=1
A0A1B0GV13	2	1	10.337	0.002377	0.002783	234.7052	0.990513	2D	HYS	11902.0659	Spectrin alpha chain_ non-erythrocytic 1 (Fragment) OS=Homo sapiens OX=9606 GN=SPTAN1 PE=1 SV=1
A0A7P0T8J3;A0A7P0T8X1;A0A7P0T	13	1	99.345	0.000283	0.000376	8.024544	0.999994	2D	0.3PG	55309.8936	Protein disulfide-isomerase OS=Homo sapiens OX=9606 GN=P4HB PE=4 SV=1
A0A7I2V2U8;A0A7I2V5D3;Q6IBS0;	2	1	14.2593	0.022805	0.024321	1.317585	0.763015	HYS	0.3PG	33992.7789	Twinfilin-2 OS=Homo sapiens OX=9606 GN=TWF2 PE=1 SV=1
O14617;A0A2R8Y4J3;A0A2R8YCY8	2	1	9.5856	0.000303	0.000399	11.56938	0.999991	HYS	0.3PG	131241.7542	AP-3 complex subunit delta-1 OS=Homo sapiens OX=9606 GN=AP3D1 PE=1 SV=1
Q8N608;J3KPP1	2	1	10.9731	3.27E-06	8.51E-06	3.304781	1	HYS	0.3PG	91458.5531	Inactive dipeptidyl peptidase 10 OS=Homo sapiens OX=9606 GN=DPP10 PE=1 SV=2
Q8WVM7	2	1	8.3679	0.169798	0.173471	1.94852	0.315884	HYS	2D	145397.0964	Cohesin subunit SA-1 OS=Homo sapiens OX=9606 GN=STAG1 PE=1 SV=3
A0A0A0MS87;Q9UN36	2	1	10.1232	3.44E-06	8.80E-06	3.425468	1	2D	0.3PG	40115.2124	Protein NDRG2 OS=Homo sapiens OX=9606 GN=NDRG2 PE=1 SV=1
Q96G03;E7ENQ8	2	1	9.8774	0.000372	0.000484	4.878517	0.999977	HYS	0.3PG	68796.7285	Phosphoglucomutase-2 OS=Homo sapiens OX=9606 GN=PGM2 PE=1 SV=4
A6NHR9;J3KTL8	1	1	5.0612	1.46E-07	1.05E-06	4.083397	1	2D	0.3PG	228084.721	Structural maintenance of chromosomes flexible hinge domain-containing protein 1 OS=Homo sapiens OX=9606 GN=SMCHD1 PE=1 SV=2

A0A0A0MQS9;A0A0A0MTC7;E5RF	42	1	394.9343	0.000668	0.000835	1.426778	0.999749	2D	HYS	205191.7716	Laminin subunit alpha-4 OS=Homo sapiens OX=9606 GN=LAMA4 PE=1 SV=1
D6R956;D6R974;D6RE83;P09936	1	1	6.4326	2.67E-06	7.31E-06	17.60962	1	0.3PG	2D	27124.9821	Ubiquitin carboxyl-terminal hydrolase OS=Homo sapiens OX=9606 GN=UCHL1 PE=1 SV=1
A0A075B7C0;A0A0C4DGQ5;K7EIV0	1	1	7.3979	0.00068	0.000846	2.749824	0.999733	0.3PG	HYS	22082.6904	Calcium-activated neutral proteinase small subunit (Fragment) OS=Homo sapiens OX=9606 GN=CAPNS1 PE=1 SV=1
A0A499FJK2;A0A7I2V5Z9;A0A7I2Y	1	1	6.6043	0.00033	0.000433	2.620765	0.999987	2D	HYS	45009.5048	Transforming growth factor beta OS=Homo sapiens OX=9606 GN=TGFB1 PE=1 SV=1
Q13401	1	1	4.3654	0.000108	0.000159	3.592317	1	2D	0.3PG	18886.9097	Putative postmeiotic segregation increased 2-like protein 3 OS=Homo sapiens OX=9606 GN=PMS2P3 PE=5 SV=2
H0Y579;P54727	1	1	6.7489	0.000196	0.000269	2.903961	0.999999	HYS	0.3PG	12050.5545	UV excision repair protein RAD23 homolog B (Fragment) OS=Homo sapiens OX=9606 GN=RAD23B PE=1 SV=1
Q15393	1	1	5.8184	0.010546	0.011552	1.62038	0.889985	2D	0.3PG	136661.0972	Splicing factor 3B subunit 3 OS=Homo sapiens OX=9606 GN=SF3B3 PE=1 SV=4
A1YPR0	1	1	5.7677	0.002074	0.002438	1.371298	0.993034	2D	0.3PG	69929.4619	Zinc finger and BTB domain-containing protein 7C OS=Homo sapiens OX=9606 GN=ZBTB7C PE=2 SV=1
F5H7G1;P07358	1	1	6.0179	0.000147	0.000209	4.778657	1	HYS	2D	62827.9867	Complement component 8 subunit beta OS=Homo sapiens OX=9606 GN=C8B PE=1 SV=1
O95954	1	1	4.9591	0.007357	0.008217	1.594082	0.930491	HYS	0.3PG	59610.9765	Formimidoyltransferase-cyclodeaminase OS=Homo sapiens OX=9606 GN=FTCD PE=1 SV=2
A8MVZ9;C9J8F3;J3KSV6;J3QKP5;K7	1	1	5.8524	2.71E-06	7.38E-06	4.70488	1	HYS	0.3PG	36637.4776	Fructose-bisphosphate aldolase OS=Homo sapiens OX=9606 GN=ALDOC PE=1 SV=1
F8VNP8	1	1	6.8893	9.71E-06	2.05E-05	21.8577	1	0.3PG	2D	6189.6017	Cleavage and polyadenylation-specificity factor subunit 6 OS=Homo sapiens OX=9606 GN=CPSF6 PE=1 SV=1
A0A075B6R2;A0A087WSY4;A0A087	1	1	6.486	1.10E-05	2.28E-05	19.62219	1	0.3PG	2D	12961.7793	Immunoglobulin heavy variable 4-4 OS=Homo sapiens OX=9606 GN=IGHV4-4 PE=3 SV=2
Q9C035	1	1	0	0.002306	0.002704	1.743402	0.991128	2D	0.3PG	57535.9209	Tripartite motif-containing protein 5 OS=Homo sapiens OX=9606 GN=TRIM5 PE=1 SV=1
Q9BRR9;R4GN15	1	1	4.8224	8.02E-06	1.74E-05	19.97347	1	2D	HYS	83943.9736	Rho GTPase-activating protein 9 OS=Homo sapiens OX=9606 GN=ARHGAP9 PE=1 SV=2
B4E2D5;F6S7C4;Q8NEM7;R4GND2	1	1	4.0794	0.039192	0.041026	2.712	0.647681	HYS	0.3PG	56855.0958	Transcription factor SPT20 homolog OS=Homo sapiens OX=9606 GN=SUPT20H PE=1 SV=1
A0A7P0TAG0;A0A7P0TAV6;P50281	1	1	5.0122	3.23E-06	8.41E-06	51.12057	1	2D	0.3PG	57214.55	Matrix metalloproteinase-14 OS=Homo sapiens OX=9606 GN=MMP14 PE=4 SV=1
A0A2R8Y6G7;E9PJP3;E9PKG6;E9PL	1	1	5.9899	1.36E-06	4.34E-06	4.441644	1	HYS	0.3PG	27746.8537	Nucleobindin-2 OS=Homo sapiens OX=9606 GN=NUCB2 PE=1 SV=1
B4DV51;B5MDF5;J3KQE5;P62826	1	1	7.0988	0.000113	0.000167	2.81413	1	HYS	0.3PG	14845.0431	GTP-binding nuclear protein Ran OS=Homo sapiens OX=9606 GN=RAN PE=1 SV=1
E9PK08;E9PKF6;E9PNN8;E9PQP7;H	1	1	5.253	0.000259	0.000348	5.543943	0.999996	HYS	0.3PG	16388.0331	Serine/threonine-protein phosphatase 6 regulatory subunit 3 (Fragment) OS=Homo sapiens OX=9606 GN=PPP6R3 PE=1 SV=1
B5MBZ8;C9J177;C9JD73;H7C003;Q	1	1	4.8162	0.000856	0.001049	3.131901	0.999408	2D	0.3PG	31471.3604	Protein phosphatase 1 regulatory subunit 7 OS=Homo sapiens OX=9606 GN=PPP1R7 PE=1 SV=1
A0A7I2YQK5;E7ETK5;H0Y4R1;P122	1	1	4.257	7.44E-05	0.000116	3.057513	1	2D	0.3PG	56080.1694	Inosine-5'-monophosphate dehydrogenase OS=Homo sapiens OX=9606 GN=IMPDH2 PE=1 SV=1
Q8WUJ3	1	1	4.1561	0.000222	0.000301	2.4151	0.999998	HYS	0.3PG	154537.7423	Cell migration-inducing and hyaluronan-binding protein OS=Homo sapiens OX=9606 GN=CEMIP PE=1 SV=2
Q99962	1	1	4.9769	0.000197	0.00027	Infinity	0.999999	0.3PG	HYS	40133.4964	Endophilin-A1 OS=Homo sapiens OX=9606 GN=SH3GL2 PE=1 SV=1
P35368	1	1	5.3129	0.012736	0.013843	36.08937	0.863868	0.3PG	HYS	57748.5779	Alpha-1B adrenergic receptor OS=Homo sapiens OX=9606 GN=ADRA1B PE=1 SV=3
A8MWK0	1	1	0	4.44E-05	7.53E-05	480.9763	1	2D	0.3PG	56814.424	Putative fatty acid desaturase 2-like protein FADS2B OS=Homo sapiens OX=9606 GN=FADS2B PE=5 SV=2
Q5T6F0;X6RBJ8	1	1	5.7385	3.11E-08	4.01E-07	Infinity	1	0.3PG	HYS	51087.5992	DDB1- and CUL4-associated factor 12 OS=Homo sapiens OX=9606 GN=DCAF12 PE=1 SV=1
C9JYB3;O75717	1	1	4.9079	1.91E-06	5.62E-06	93.1781	1	HYS	2D	18462.437	WD repeat and HMG-box DNA-binding protein 1 (Fragment) OS=Homo sapiens OX=9606 GN=WDHD1 PE=1 SV=1
Q13009	1	1	4.9088	1.78E-05	3.45E-05	3.965352	1	0.3PG	HYS	178820.032	Rho guanine nucleotide exchange factor TIAM1 OS=Homo sapiens OX=9606 GN=TIAM1 PE=1 SV=2
B0QZK8;Q5SSJ5;Q5SWC8	1	1	6.1033	0.000324	0.000425	Infinity	0.999988	2D	HYS	13683.7955	Heterochromatin protein 1-binding protein 3 (Fragment) OS=Homo sapiens OX=9606 GN=HP1BP3 PE=1 SV=1
A0A669KBG0;Q8NBP7	1	1	4.9582	7.78E-05	0.000121	3.001856	1	0.3PG	2D	61718.8984	Proprotein convertase 9 OS=Homo sapiens OX=9606 GN=PCSK9 PE=1 SV=1
A0A2R8Y214;P14138;Q4FAT2	1	1	9.3472	0.011475	0.01252	66.08803	0.878721	2D	HYS	21743.8294	Endothelin-3 OS=Homo sapiens OX=9606 GN=EDN3 PE=1 SV=1
Q9Y2G7	1	1	4.1622	0.000485	0.000618	197.9111	0.999927	HYS	2D	63496.6056	Zinc finger protein 30 homolog OS=Homo sapiens OX=9606 GN=ZFP30 PE=1 SV=1
H3BSM0;Q53FZ2	1	1	4.6125	1.04E-06	3.52E-06	33.01852	1	HYS	2D	65991.0871	Acyl-coenzyme A synthetase ACSM3_ mitochondrial (Fragment) OS=Homo sapiens OX=9606 GN=ACSM3 PE=1 SV=1
Q5FYB1	1	1	5.477	0.012189	0.013282	6.79198	0.870246	0.3PG	HYS	64486.5744	Arylsulfatase I OS=Homo sapiens OX=9606 GN=ARSI PE=1 SV=1
Q96KC9	1	1	4.8093	0.006963	0.007819	92.30914	0.935612	0.3PG	HYS	43003.0082	Calcium-binding and spermatid-specific protein 1 OS=Homo sapiens OX=9606 GN=CABS1 PE=2 SV=3
A0A0G2JJQ8;P13762;X5D2U9	1	1	4.8944	0.003033	0.003516	160.7237	0.98418	0.3PG	2D	30370.3587	HLA class II histocompatibility antigen_ DR beta 4 chain OS=Homo sapiens OX=9606 GN=HLA-DRB4 PE=1 SV=1
M0QWZ7;Q9NP81	1	1	5.0746	0.005872	0.006637	76.46779	0.949819	2D	0.3PG	58638.741	Seryl-tRNA synthetase OS=Homo sapiens OX=9606 GN=SARS2 PE=1 SV=1
M0R1B1	1	1	5.0586	0.000238	0.000321	7.019385	0.999997	HYS	0.3PG	5112.7247	Zinc finger protein 649 (Fragment) OS=Homo sapiens OX=9606 GN=ZNF649 PE=4 SV=1
E9PN19;O00214	1	1	4.3699	1.93E-05	3.71E-05	Infinity	1	2D	HYS	32803.6223	Galectin OS=Homo sapiens OX=9606 GN=LGALS8 PE=1 SV=1
J3QT10;Q8TBK2	1	1	4.6547	0.000937	0.001141	2.097978	0.999208	2D	0.3PG	46002.4936	N-lysine methyltransferase SETD6 OS=Homo sapiens OX=9606 GN=SETD6 PE=1 SV=1
Q8NHP6;R4GMN1	1	1	5.4028	0.000188	0.000261	3.267922	0.999999	0.3PG	HYS	60088.4416	Motile sperm domain-containing protein 2 OS=Homo sapiens OX=9606 GN=MOSPD2 PE=1 SV=1
Q92851	1	1	5.6676	0.000693	0.000855	2.683306	0.999713	2D	0.3PG	59635.0102	Caspase-10 OS=Homo sapiens OX=9606 GN=CASP10 PE=1 SV=3
Q9H489	2	1	8.7248	0.043659	0.045534	15.20502	0.623103	2D	HYS	39857.7244	Putative testis-specific Y-encoded-like protein 3 OS=Homo sapiens OX=9606 GN=TSPY26P PE=5 SV=1
A0A1B0GU38;P50452	1	1	5.4884	0.000678	0.000846	9.290231	0.999735	HYS	0.3PG	29856.8595	Serpin B8 OS=Homo sapiens OX=9606 GN=SERPINB8 PE=1 SV=1
H7C1D9	1	1	5.201	5.12E-05	8.54E-05	23.63746	1	0.3PG	2D	113796.6529	Alstrom syndrome protein 1 (Fragment) OS=Homo sapiens OX=9606 GN=ALMS1 PE=1 SV=1
C9JZF7	1	1	11.0956	0.005148	0.005843	1.411532	0.959124	HYS	0.3PG	9948.629	Chondroitin sulfate glucuronyltransferase (Fragment) OS=Homo sapiens OX=9606 GN=CHPF2 PE=1 SV=1
H7BZJ3	1	1	7.3074	4.09E-07	1.86E-06	2.414764	1	HYS	0.3PG	13747.443	Protein disulfide-isomerase A3 (Fragment) OS=Homo sapiens OX=9606 GN=PDIA3 PE=1 SV=1
S4R396	1	1	13.4162	2.16E-08	3.19E-07	5.09845	1	0.3PG	2D	7434.9514	Polypeptide N-acetylgalactosaminyltransferase 6 (Fragment) OS=Homo sapiens OX=9606 GN=GALNT6 PE=1 SV=1
P00747	1	1	6.6087	0.000271	0.000361	1.763818	0.999995	0.3PG	2D	93306.4078	Plasminogen OS=Homo sapiens OX=9606 GN=PLG PE=1 SV=2
C9J5G4	1	1	16.2428	4.01E-07	1.86E-06	12.43432	1	2D	0.3PG	16348.4987	Follistatin-related protein 1 (Fragment) OS=Homo sapiens OX=9606 GN=FSTL1 PE=1 SV=8
A0A0A0MQV8;B4DGD8;C9IZ08;F8V	1	1	5.9217	2.34E-06	6.56E-06	3.860812	1	2D	0.3PG	70070.1198	GTPase-activating protein and VPS9 domain-containing protein 1 OS=Homo sapiens OX=9606 GN=GAPVD1 PE=1 SV=1
F8VPE8;F8VU65;F8VW21;P05388;	1	1	12.4516	0.219217	0.222622	1.32548	0.265399	2D	0.3PG	16794.6781	60S acidic ribosomal protein P0 (Fragment) OS=Homo sapiens OX=9606 GN=RPLP0 PE=1 SV=1
P57796	2	1	8.5093	4.79E-06	1.13E-05	3.431706	1	0.3PG	2D	30490.1012	Calcium-binding protein 4 OS=Homo sapiens OX=9606 GN=CABP4 PE=2 SV=2
A0A087WXM3;M0R0W4;P59047	1	1	5.2098	0.000405	0.000524	4.977896	0.999966	2D	0.3PG	137236.7947	NACHT_ LRR and PYD domains-containing protein 5 OS=Homo sapiens OX=9606 GN=NLRP5 PE=1 SV=1
K7EJ80;K7EJH5	1	1	6.8232	8.21E-07	2.91E-06	10.8001	1	0.3PG	2D	5376.1055	Zinc finger protein 763 OS=Homo sapiens OX=9606 GN=ZNF763 PE=4 SV=1
A0A0C4DH25;P01619	1	1	17.0912	2.05E-08	3.10E-07	6.348404	1	0.3PG	2D	12629.139	Immunoglobulin kappa variable 3D-20 OS=Homo sapiens OX=9606 GN=IGKV3D-20 PE=3 SV=1
A0A0A0MSF3	1	1	13.0724	8.86E-10	5.10E-08	19.88168	1	0.3PG	2D	39454.9308	Cyclin-L2 OS=Homo sapiens OX=9606 GN=CCNL2 PE=1 SV=1
Q86VP6	2	1	14.5672	0.000681	0.000846	2.045471	0.999732	0.3PG	2D	138086.7865	Cullin-associated NEDD8-dissociated protein 1 OS=Homo sapiens OX=9606 GN=CAND1 PE=1 SV=2
F5H8H2;Q03426	1	1	5.0581	5.72E-05	9.38E-05	1.996515	1	2D	0.3PG	37571.061	Mevalonate kinase OS=Homo sapiens OX=9606 GN=MVK PE=1 SV=1
O95455	1	1	4.597	4.10E-10	4.97E-08	123.1674	1	HYS	2D	40556.1263	dTDP-D-glucose 4_6-dehydratase OS=Homo sapiens OX=9606 GN=TGDS PE=1 SV=1
A0A0G2JLQ8;A0A0G2JLX3;A0A0G2	2	1	10.9556	0.003036	0.003516	151.9565	0.984146	2D	HYS	101545.2605	NACHT_ LRR and PYD domains-containing protein 2 (Fragment) OS=Homo sapiens OX=9606 GN=NLRP2 PE=1 SV=1
Q6P1X5;H0YC37	2	1	9.3615	0.021013	0.022467	20.65627	0.778862	2D	HYS	138454.4054	Transcription initiation factor TFIID subunit 2 OS=Homo sapiens OX=9606 GN=TAF2 PE=1 SV=3
A0A2R8YEL6;E1CEI4;P48506;A0A0	2	1	10.2661	7.58E-05	0.000118	7.350652	1	0.3PG	HYS	73844.463	Glutamate--cysteine ligase (Fragment) OS=Homo sapiens OX=9606 GN=GCLC PE=1 SV=1
Q9C0B0	1	1	9.3258	2.31E-06	6.52E-06	5.217529	1	2D	0.3PG	89852.399	RING finger protein unkempt homolog OS=Homo sapiens OX=9606 GN=UNK PE=1 SV=2
Q5TE25;Q9NWM0	1	1	4.855	9.04E-06	1.92E-05	4.777207	1	2D	0.3PG	50350.0547	Spermine oxidase (Fragment) OS=Homo sapiens OX=9606 GN=SMOX PE=1 SV=1
A0A0X1KG76	1	1	4.4545	3.82E-08	4.51E-07	20.37567	1	0.3PG	2D	21286.7447	Membrane frizzled-related protein (Fragment) OS=Homo sapiens OX=9606 GN=MFRP PE=1 SV=1
A6NK59	1	1	5.9761	3.22E-05	5.67E-05	2.548801	1	0.3PG	2D	66243.807	Ankyrin repeat and SOCS box protein 14 OS=Homo sapiens OX=9606 GN=ASB14 PE=2 SV=2
F8WB07;F8WEK5;Q9H0V9	1	1	5.1807	0.024684	0.026259	1.349896	0.747222	HYS	0.3PG	19049.924	VIP36-like protein OS=Homo sapiens OX=9606 GN=LMAN2L PE=1 SV=1
Q8IWZ4	1	1	5.7658	1.75E-07	1.17E-06	34.91936	1	0.3PG	2D	25238.9238	Tripartite motif-containing protein 48 OS=Homo sapiens OX=9606 GN=TRIM48 PE=1 SV=2
K7EMJ5;K7EPS3;K7ERP1;Q9GZN7	1	1	5.1007	6.38E-05	0.000102	1.643249	1	HYS	0.3PG	12763.6104	Protein rogdi homolog OS=Homo sapiens OX=9606 GN=ROGDI PE=1 SV=1
F8WAS9;Q6P1Q9;Q96IZ6	1	1	5.834	7.81E-09	1.75E-07	10.72138	1	0.3PG	HYS	8698.5279	tRNA N(3)-methylcytidine methyltransferase METTL2B OS=Homo sapiens OX=9606 GN=METTL2B PE=1 SV=1

A0A0C4DH72;A0A0C4DH73;P0159	1	1	7.5227	4.77E-05	8.02E-05	3.332801	1	0.3PG	2D	12868.5946	Immunoglobulin kappa variable 1-6 OS=Homo sapiens OX=9606 GN=IGKV1-6 PE=3 SV=1
I3L1A8;I3L2T0;I3L3N8;I3L437;Q8TE	1	1	5.0597	3.47E-07	1.74E-06	20.41973	1	0.3PG	2D	10768.1345	Elongator complex protein 5 (Fragment) OS=Homo sapiens OX=9606 GN=ELP5 PE=1 SV=1
M0R0S1;M0R2P6;Q8TBC3	1	1	4.4442	1.45E-06	4.54E-06	4.31245	1	2D	0.3PG	66424.2369	SH3KBP1-binding protein 1 (Fragment) OS=Homo sapiens OX=9606 GN=SHKBP1 PE=1 SV=1
F8WAK3;Q13495	1	1	5.122	0.019887	0.021343	1.827854	0.789249	2D	0.3PG	15300.5055	Mastermind-like domain-containing protein 1 (Fragment) OS=Homo sapiens OX=9606 GN=MAMLD1 PE=1 SV=1
F2Z393;P37837	1	1	5.7144	0.000117	0.000172	1.740407	1	HYS	0.3PG	35557.0154	Transaldolase OS=Homo sapiens OX=9606 GN=TALDO1 PE=1 SV=1
O60888	1	1	6.7704	0.19143	0.194868	1.134099	0.291727	2D	HYS	19230.4191	Protein CutA OS=Homo sapiens OX=9606 GN=CUTA PE=1 SV=2
Q5T8V1;Q8N4J0	1	1	4.465	5.87E-07	2.33E-06	7.098464	1	HYS	0.3PG	28094.0081	Carnosine N-methyltransferase (Fragment) OS=Homo sapiens OX=9606 GN=CARNMT1 PE=1 SV=1
B1AQT1;D6RFK6	1	1	5.1133	1.49E-07	1.06E-06	20.30176	1	0.3PG	2D	21615.6251	Ankyrin-3 (Fragment) OS=Homo sapiens OX=9606 GN=ANK3 PE=1 SV=1
Q86VW0	1	1	5.3582	5.34E-06	1.24E-05	4.656189	1	2D	0.3PG	80089.7827	SEC14 domain and spectrin repeat-containing protein 1 OS=Homo sapiens OX=9606 GN=SESTD1 PE=1 SV=2
P11172	1	1	4.903	1.10E-05	2.28E-05	2.822067	1	HYS	0.3PG	52677.8717	Uridine 5'-monophosphate synthase OS=Homo sapiens OX=9606 GN=UMPS PE=1 SV=1
Q96J77	1	1	9.535	3.17E-07	1.61E-06	19.94371	1	2D	0.3PG	15617.0496	Tumor protein D55 OS=Homo sapiens OX=9606 GN=TPD52L3 PE=1 SV=2
A0A7P0T897;A0A7P0T9P7;P98155	1	1	4.6935	4.60E-07	1.98E-06	487.6722	1	0.3PG	2D	95069.3842	Very low-density lipoprotein receptor OS=Homo sapiens OX=9606 GN=VLDLR PE=4 SV=1
A0A0U1RR32;A0A0U1RRH7;A0A3B	1	1	5.5549	0.000163	0.000228	1.492607	1	HYS	0.3PG	18481.5797	Histone H2A OS=Homo sapiens OX=9606 GN=hCG_2039566 PE=3 SV=1
D6REX3;D6RHZ5;O94979	1	1	5.3644	5.40E-08	5.43E-07	9.459727	1	0.3PG	2D	137195.9572	Protein transport protein Sec31A OS=Homo sapiens OX=9606 GN=SEC31A PE=1 SV=1
Q7Z2Z1	1	1	4.9655	1.43E-05	2.84E-05	5.561438	1	0.3PG	2D	212853.1108	Treslin OS=Homo sapiens OX=9606 GN=TICRR PE=1 SV=2
A0A0G2JQ41;A0A1C7CYZ0;B7Z683	1	1	4.4195	2.46E-06	6.83E-06	8.258981	1	2D	0.3PG	89428.8519	Active breakpoint cluster region-related protein (Fragment) OS=Homo sapiens OX=9606 GN=ABR PE=1 SV=1
O60266;U3KQ91	2	1	9.3708	1.66E-07	1.13E-06	6.507047	1	2D	0.3PG	130157.8855	Adenylate cyclase type 3 OS=Homo sapiens OX=9606 GN=ADCY3 PE=1 SV=3
A0A6Q8PF34;Q96PZ2	1	1	4.7542	0.00741	0.008266	Infinity	0.929796	2D	HYS	37240.337	Serine protease FAM111A (Fragment) OS=Homo sapiens OX=9606 GN=FAM111A PE=1 SV=1
Q04695;F5GWP8	6	1	41.0564	0.000221	0.000301	2.943304	0.999998	0.3PG	2D	48390.8844	Keratin_ type I cytoskeletal 17 OS=Homo sapiens OX=9606 GN=KRT17 PE=1 SV=2
Q9P253	3	1	15.9725	4.92E-06	1.16E-05	2.431866	1	HYS	0.3PG	111554.461	Vacuolar protein sorting-associated protein 18 homolog OS=Homo sapiens OX=9606 GN=VPS18 PE=1 SV=2
A0A1B0GV47;Q7Z4S6	6	1	32.7646	0.007181	0.008042	111.2731	0.932781	2D	HYS	186021.3047	Kinesin-like protein KIF21A (Fragment) OS=Homo sapiens OX=9606 GN=KIF21A PE=1 SV=1
G3XAM7;P35221	3	1	12.5432	2.32E-07	1.38E-06	7.123936	1	0.3PG	2D	93634.2818	Catenin (Cadherin-associated protein)_ alpha 1_ 102kDa_ isoform CRA_a OS=Homo sapiens OX=9606 GN=CTNNA1 PE=1 SV=1
Q07021;I3L3B0;I3L3Q7	3	1	22.4978	2.49E-08	3.42E-07	36.63132	1	0.3PG	2D	31761.4626	Complement component 1 Q subcomponent-binding protein_ mitochondrial OS=Homo sapiens OX=9606 GN=C1QBP PE=1 SV=1
P08575;A0A075B788	3	1	24.1307	0.079042	0.081635	Infinity	0.484828	2D	HYS	148969.3939	Receptor-type tyrosine-protein phosphatase C OS=Homo sapiens OX=9606 GN=PTPRC PE=1 SV=3
P35712;E9PN02;E9PQ78;E9PQL4	3	1	15.1119	4.15E-05	7.15E-05	3.376887	1	2D	0.3PG	92320.6524	Transcription factor SOX-6 OS=Homo sapiens OX=9606 GN=SOX6 PE=1 SV=3
A0A0A0MRF9;P16885	5	1	27.445	0.000188	0.000261	1.277296	0.999999	2D	HYS	147257.5776	1-phosphatidylinositol 4_5-bisphosphate phosphodiesterase gamma OS=Homo sapiens OX=9606 GN=PLCG2 PE=1 SV=1
H0Y991	3	1	25.5347	0.001857	0.002191	12.99106	0.994642	2D	HYS	23269.1014	Collagen alpha-1(XII) chain (Fragment) OS=Homo sapiens OX=9606 GN=COL12A1 PE=1 SV=1
E7EMB3	6	1	51.2949	0.183571	0.187317	1.354668	0.300082	2D	0.3PG	21860.4064	Calmodulin-2 OS=Homo sapiens OX=9606 GN=CALM2 PE=1 SV=1
K7ELY9	3	1	23.1281	0.007098	0.00796	147.9073	0.933853	2D	HYS	27069.9674	Beclin-1 (Fragment) OS=Homo sapiens OX=9606 GN=BECN1 PE=1 SV=1
A0A7I2V4L7;B1ANR0;Q13310;A0A7	5	1	31.7476	7.50E-07	2.74E-06	11.55008	1	0.3PG	2D	66728.5061	Polyadenylate-binding protein OS=Homo sapiens OX=9606 GN=PABPC4 PE=1 SV=1
Q8N264	3	1	12.3585	5.83E-08	5.57E-07	7.777853	1	0.3PG	HYS	84885.7504	Rho GTPase-activating protein 24 OS=Homo sapiens OX=9606 GN=ARHGAP24 PE=1 SV=2
P27816;H7C456	3	1	15.4302	3.50E-07	1.74E-06	10.49804	1	0.3PG	HYS	121518.4468	Microtubule-associated protein 4 OS=Homo sapiens OX=9606 GN=MAP4 PE=1 SV=3
A0A087WTF3;A0A087WZ65;A0A08	3	1	15.3652	0.000289	0.000383	4.086602	0.999993	HYS	0.3PG	180615.533	Ankyrin-3 (Fragment) OS=Homo sapiens OX=9606 GN=ANK3 PE=1 SV=1
P0DPH7;P0DPH8	6	1	41.1574	1.61E-07	1.11E-06	Infinity	1	0.3PG	2D	50643.9551	Tubulin alpha-3C chain OS=Homo sapiens OX=9606 GN=TUBA3C PE=1 SV=1
O14976	2	1	5.0733	1.42E-08	2.36E-07	36.74171	1	0.3PG	2D	144673.4805	Cyclin-G-associated kinase OS=Homo sapiens OX=9606 GN=GAK PE=1 SV=2
P36888;E7ER61	3	1	20.6137	0.000258	0.000347	Infinity	0.999996	2D	HYS	114785.5521	Receptor-type tyrosine-protein kinase FLT3 OS=Homo sapiens OX=9606 GN=FLT3 PE=1 SV=2
Q8N1A0	2	1	11.4901	4.08E-07	1.86E-06	8.397381	1	0.3PG	2D	34328.9456	Keratin-like protein KRT222 OS=Homo sapiens OX=9606 GN=KRT222 PE=1 SV=1
P35790	2	1	14.4838	1.67E-06	5.06E-06	15.24023	1	0.3PG	HYS	52705.3607	Choline kinase alpha OS=Homo sapiens OX=9606 GN=CHKA PE=1 SV=3
Q4G0A6	3	1	17.216	1.29E-07	9.89E-07	11.00911	1	2D	0.3PG	85284.2692	Probable ubiquitin carboxyl-terminal hydrolase MINDY-4 OS=Homo sapiens OX=9606 GN=MINDY4 PE=1 SV=2
Q9Y6D9	4	1	24.727	0.000168	0.000235	118.8584	1	0.3PG	2D	83352.1676	Mitotic spindle assembly checkpoint protein MAD1 OS=Homo sapiens OX=9606 GN=MAD1L1 PE=1 SV=2
Q9Y4E6	4	1	23.555	4.37E-07	1.93E-06	7.618172	1	2D	0.3PG	166262.7344	WD repeat-containing protein 7 OS=Homo sapiens OX=9606 GN=WDR7 PE=1 SV=2
H7C0D9	3	1	20.7629	0.004575	0.005227	272.6324	0.966328	2D	0.3PG	34629.7161	DNA-directed RNA polymerase (Fragment) OS=Homo sapiens OX=9606 GN=POLR1B PE=1 SV=8
Q8NEV8;E9PPH6	5	1	25.6833	7.57E-06	1.67E-05	2.620733	1	2D	0.3PG	223888.1735	Exophilin-5 OS=Homo sapiens OX=9606 GN=EXPH5 PE=1 SV=3
Q8TDG2	2	1	15.5228	0.000145	0.000208	2.459732	1	HYS	2D	42266.6496	Actin-related protein T1 OS=Homo sapiens OX=9606 GN=ACTRT1 PE=2 SV=2
Q6UVY6	3	1	14.084	1.16E-06	3.80E-06	5.863212	1	HYS	0.3PG	70565.0506	DBH-like monooxygenase protein 1 OS=Homo sapiens OX=9606 GN=MOXD1 PE=1 SV=1
Q01546	3	1	29.1566	4.40E-05	7.48E-05	42.4391	1	2D	0.3PG	66411.1179	Keratin_ type II cytoskeletal 2 oral OS=Homo sapiens OX=9606 GN=KRT76 PE=1 SV=2
Q9Y281	5	1	62.901	2.74E-05	4.93E-05	2.572763	1	HYS	0.3PG	18850.6967	Cofilin-2 OS=Homo sapiens OX=9606 GN=CFL2 PE=1 SV=1
M0QYJ9	3	1	20.7429	0.000175	0.000244	2.696732	1	2D	0.3PG	15594.7514	SH3KBP1-binding protein 1 OS=Homo sapiens OX=9606 GN=SHKBP1 PE=1 SV=1
P08729	4	1	42.42	0.000384	0.0005	1.736083	0.999973	2D	0.3PG	51442.7715	Keratin_ type II cytoskeletal 7 OS=Homo sapiens OX=9606 GN=KRT7 PE=1 SV=5
Q99683	3	1	17.558	1.05E-06	3.53E-06	7.644723	1	0.3PG	2D	155848.7625	Mitogen-activated protein kinase kinase kinase 5 OS=Homo sapiens OX=9606 GN=MAP3K5 PE=1 SV=1
Q9Y2I6	3	1	15.1772	3.03E-07	1.56E-06	3.121342	1	2D	0.3PG	157541.3685	Ninein-like protein OS=Homo sapiens OX=9606 GN=NINL PE=1 SV=2
A0A0G2JLU6;A0A0G2JM27;A0A0G	3	1	27.4803	7.34E-05	0.000115	3.764466	1	2D	0.3PG	32720.2647	Immunoglobulin alpha Fc receptor OS=Homo sapiens OX=9606 GN=FCAR PE=1 SV=1
E9PNL8;Q13574	3	1	13.8041	0.008639	0.009549	1.793548	0.913928	2D	HYS	79726.0994	Diacylglycerol kinase OS=Homo sapiens OX=9606 GN=DGKZ PE=1 SV=1
A0A5H1ZRS2;A0A075B6P5;A0A075	2	1	15.6391	7.61E-08	6.81E-07	4.876548	1	0.3PG	2D	11157.4496	Immunoglobulin kappa variable 2D-28 OS=Homo sapiens OX=9606 GN=IGKV2D-28 PE=1 SV=1
H0YNN7;Q9UPX6	3	1	21.017	1.17E-06	3.80E-06	3.655023	1	2D	0.3PG	97254.4664	Major intrinsically disordered Notch2-binding receptor 1 OS=Homo sapiens OX=9606 GN=MINAR1 PE=3 SV=1
P07093;C9JN98	3	1	27.3248	0.006863	0.007716	98.83491	0.936922	2D	0.3PG	44230.4168	Glia-derived nexin OS=Homo sapiens OX=9606 GN=SERPINE2 PE=1 SV=1
A0A2R8YF49;Q12774;A0A2R8YFR7	3	1	19.5631	2.73E-06	7.38E-06	7.437702	1	0.3PG	2D	177952.2628	Rho guanine nucleotide exchange factor 5 OS=Homo sapiens OX=9606 GN=ARHGEF5 PE=1 SV=1
Q8IZY2	5	1	19.6204	0.000123	0.00018	19.36082	1	HYS	2D	236402.9932	Phospholipid-transporting ATPase ABCA7 OS=Homo sapiens OX=9606 GN=ABCA7 PE=1 SV=3
H3BQA7	3	1	15.4558	4.95E-05	8.29E-05	Infinity	1	0.3PG	HYS	71982.2912	Obscurin (Fragment) OS=Homo sapiens OX=9606 GN=OBSCN PE=1 SV=3
P60981;F6RFD5	3	1	26.2114	0.002903	0.003381	32.25668	0.985513	2D	HYS	18961.9812	Destrin OS=Homo sapiens OX=9606 GN=DSTN PE=1 SV=3
F8W705;J3KQ37;O14715	3	1	15.1529	8.70E-05	0.000133	10.96228	1	2D	0.3PG	104370.6142	RANBP2-like and GRIP domain-containing protein 8 OS=Homo sapiens OX=9606 GN=RGPD8 PE=1 SV=1
I3L2J8;Q9UPN4	3	1	12.1727	4.44E-06	1.07E-05	3.049813	1	0.3PG	2D	118154.2341	Centrosomal protein of 131 kDa OS=Homo sapiens OX=9606 GN=CEP131 PE=1 SV=1
O43683;A0A590UK50;C9IYH4;C9JQ	3	1	18.062	3.88E-07	1.84E-06	22.36573	1	HYS	0.3PG	123515.9841	Mitotic checkpoint serine/threonine-protein kinase BUB1 OS=Homo sapiens OX=9606 GN=BUB1 PE=1 SV=1
A2A3D8;A2A3E6;A2A3E7;A2A3E8;E	5	1	28.0335	0.000548	0.000692	2.966244	0.999881	2D	0.3PG	218429.2357	Protocadherin-15 OS=Homo sapiens OX=9606 GN=PCDH15 PE=1 SV=3
Q96ER3;E9PMD2;E9PRZ1;H0YEK5;J	3	1	21.2553	2.35E-05	4.37E-05	2.93531	1	2D	0.3PG	54470.5255	Protein SAAL1 OS=Homo sapiens OX=9606 GN=SAAL1 PE=1 SV=2
P02748	4	1	37.2441	2.58E-07	1.44E-06	256.4762	1	0.3PG	2D	64656.1742	Complement component C9 OS=Homo sapiens OX=9606 GN=C9 PE=1 SV=2
Q13136;E9PJZ7;A0A3B3ITS2;E9PID	3	1	22.7419	3.40E-11	9.65E-09	28.19475	1	0.3PG	HYS	136349.2836	Liprin-alpha-1 OS=Homo sapiens OX=9606 GN=PPFIA1 PE=1 SV=1
A0A2U3TZL8;A0A7I2V5Y5;H7BYN4	5	1	23.7202	0.000298	0.000394	2.521727	0.999992	HYS	0.3PG	89538.6685	Kinesin-like protein OS=Homo sapiens OX=9606 GN=KIF23 PE=1 SV=1
Q9Y608;C9JSU1;H7C3N9	3	1	20.55	0.000267	0.000357	1.612473	0.999995	HYS	0.3PG	82399.6802	Leucine-rich repeat flightless-interacting protein 2 OS=Homo sapiens OX=9606 GN=LRRFIP2 PE=1 SV=1
P52823	3	1	17.7119	2.19E-05	4.14E-05	147.3792	1	HYS	0.3PG	28248.7583	Stanniocalcin-1 OS=Homo sapiens OX=9606 GN=STC1 PE=1 SV=1
Q99456	5	1	36.6151	1.46E-05	2.89E-05	4.694052	1	0.3PG	2D	53625.5628	Keratin_ type I cytoskeletal 12 OS=Homo sapiens OX=9606 GN=KRT12 PE=1 SV=1
B5MBY2;Q9Y6T7	5	1	28.4478	2.73E-06	7.38E-06	10.63826	1	HYS	0.3PG	92064.1436	Diacylglycerol kinase OS=Homo sapiens OX=9606 GN=DGKB PE=1 SV=1

P19099	6	1	36.471	1.17E-05	2.40E-05	6.68075	1	0.3PG	2D	57959.4512	Cytochrome P450 11B2_ mitochondrial OS=Homo sapiens OX=9606 GN=CYP11B2 PE=1 SV=3
P22223	3	1	14.356	9.13E-05	0.000139	2.017245	1	2D	HYS	91931.7632	Cadherin-3 OS=Homo sapiens OX=9606 GN=CDH3 PE=1 SV=2
Q9P1U1	3	1	13.9073	0.412231	0.41467	1.54659	0.157787	2D	HYS	48121.0317	Actin-related protein 3B OS=Homo sapiens OX=9606 GN=ACTR3B PE=2 SV=1
P02775	3	1	21.8682	0.000756	0.000927	3.125063	0.999611	0.3PG	2D	14179.3758	Platelet basic protein OS=Homo sapiens OX=9606 GN=PPBP PE=1 SV=3
A0A140T913;A0A140T9I0;A0A140T	2	1	11.4528	1.96E-07	1.25E-06	11.00656	1	2D	0.3PG	41734.6366	HLA class I histocompatibility antigen_ A alpha chain OS=Homo sapiens OX=9606 GN=HLA-A PE=1 SV=1
Q14914;F2Z3J9	2	1	15.0707	1.96E-06	5.71E-06	4.244345	1	0.3PG	2D	36098.0298	Prostaglandin reductase 1 OS=Homo sapiens OX=9606 GN=PTGR1 PE=1 SV=2
A0A075B727;P16284	2	1	9.1332	0.00047	0.000599	2.24114	0.999936	2D	0.3PG	26504.2785	Platelet endothelial cell adhesion molecule (Fragment) OS=Homo sapiens OX=9606 GN=PECAM1 PE=1 SV=1
D6RBW9;D6RDX8;H0Y9A5;J3KNG2;	3	1	18.3907	0.000201	0.000275	2.383682	0.999999	HYS	2D	29348.6453	Tetraspanin (Fragment) OS=Homo sapiens OX=9606 GN=TSPAN17 PE=1 SV=1
E9PF10;O75694	2	1	16.6229	4.14E-07	1.86E-06	10.06529	1	0.3PG	2D	149519.917	Nuclear pore complex protein Nup155 OS=Homo sapiens OX=9606 GN=NUP155 PE=1 SV=1
O94856	4	1	19.0223	2.42E-09	9.35E-08	Infinity	1	2D	0.3PG	150882.4059	Neurofascin OS=Homo sapiens OX=9606 GN=NFASC PE=1 SV=4
P08727	7	1	45.4681	7.50E-06	1.66E-05	8.085146	1	0.3PG	HYS	44106.0704	Keratin_ type I cytoskeletal 19 OS=Homo sapiens OX=9606 GN=KRT19 PE=1 SV=4
A0A7I2V454;A0A7I2V5R1;Q8TD19	3	1	20.9476	0.000141	0.000204	4.091002	1	2D	0.3PG	95857.1295	Serine/threonine-protein kinase Nek9 OS=Homo sapiens OX=9606 GN=NEK9 PE=1 SV=1
O14498;H0YL90;H0YN67	3	1	22.2436	6.75E-08	6.31E-07	2.988998	1	2D	0.3PG	46624.6475	Immunoglobulin superfamily containing leucine-rich repeat protein OS=Homo sapiens OX=9606 GN=ISLR PE=2 SV=1
O14950	5	1	39.0845	0.000236	0.00032	15.90598	0.999997	2D	HYS	19836.2475	Myosin regulatory light chain 12B OS=Homo sapiens OX=9606 GN=MYL12B PE=1 SV=2
J3KN67;A0A087WWU8	7	1	51.9055	0.458573	0.460741	1.350238	0.142188	2D	HYS	33450.7124	Tropomyosin alpha-3 chain OS=Homo sapiens OX=9606 GN=TPM3 PE=1 SV=1
O15259	2	1	13.6373	6.07E-05	9.83E-05	2.146546	1	HYS	2D	83869.7888	Nephrocystin-1 OS=Homo sapiens OX=9606 GN=NPHP1 PE=1 SV=1
K7ERE3;P13646	4	1	28.1508	0.007494	0.008349	8.727574	0.928699	HYS	2D	45602.7609	Keratin_ type I cytoskeletal 13 OS=Homo sapiens OX=9606 GN=KRT13 PE=1 SV=1
Q8N0Y7	5	1	55.4722	0.025973	0.027562	7.650779	0.736842	0.3PG	2D	28947.9826	Probable phosphoglycerate mutase 4 OS=Homo sapiens OX=9606 GN=PGAM4 PE=3 SV=1
Q8IZU2	4	1	17.9277	9.72E-06	2.05E-05	7.570672	1	2D	0.3PG	150212.7248	WD repeat-containing protein 17 OS=Homo sapiens OX=9606 GN=WDR17 PE=2 SV=2
E9PQG4	3	1	13.8391	0.000148	0.000211	1.862012	1	HYS	0.3PG	85696.2682	Myomegalin OS=Homo sapiens OX=9606 GN=PDE4DIP PE=1 SV=2
G3XA83;Q9UBX8	4	1	28.172	1.03E-06	3.50E-06	9.927501	1	2D	0.3PG	40745.6303	Beta-1_4-galactosyltransferase 6 OS=Homo sapiens OX=9606 GN=B4GALT6 PE=1 SV=1
Q7L7X3;J3QS76	2	1	15.4948	0.00145	0.001731	4.146067	0.997156	HYS	0.3PG	116526.7263	Serine/threonine-protein kinase TAO1 OS=Homo sapiens OX=9606 GN=TAOK1 PE=1 SV=1
O15457	4	1	30.1437	1.84E-06	5.46E-06	4.37153	1	2D	0.3PG	105839.3067	MutS protein homolog 4 OS=Homo sapiens OX=9606 GN=MSH4 PE=1 SV=2
P06753;A0A2R2Y2Q3;A0A494C0P6	7	1	50.896	2.97E-05	5.26E-05	5.988691	1	0.3PG	2D	33007.0662	Tropomyosin alpha-3 chain OS=Homo sapiens OX=9606 GN=TPM3 PE=1 SV=2
P15531	4	1	32.081	0.000196	0.000269	1.983983	0.999999	HYS	0.3PG	17319.8447	Nucleoside diphosphate kinase A OS=Homo sapiens OX=9606 GN=NME1 PE=1 SV=1
Q12965;H0YNQ8	2	1	9.4863	6.59E-07	2.50E-06	4.221372	1	0.3PG	2D	127632.3797	Unconventional myosin-Ie OS=Homo sapiens OX=9606 GN=MYO1E PE=1 SV=2
A0A087X1X7;E9PK01;E9PQZ1;E9PR	2	1	19.6004	0.001858	0.002191	1.402587	0.994635	HYS	0.3PG	69738.6956	Elongation factor 1-delta OS=Homo sapiens OX=9606 GN=EEF1D PE=1 SV=1
O15056;H7BY56	4	1	25.4495	0.001794	0.002124	2.28305	0.995078	0.3PG	2D	166678.7192	Synaptojanin-2 OS=Homo sapiens OX=9606 GN=SYNJ2 PE=1 SV=3
Q58FF7	4	1	25.0794	0.000106	0.000157	44.4387	1	0.3PG	2D	68667.1353	Putative heat shock protein HSP 90-beta-3 OS=Homo sapiens OX=9606 GN=HSP90AB3P PE=5 SV=1
E9PPG9;E9PQ57;P78406;B0QZ36;B	2	1	16.0181	0.000415	0.000535	1.941648	0.999962	HYS	2D	24460.4544	Rae1 protein homolog OS=Homo sapiens OX=9606 GN=RAE1 PE=1 SV=1
E9PFT6;P02042;E9PEW8	4	1	41.9681	4.11E-13	3.49E-10	Infinity	1	0.3PG	2D	15590.8282	Hemoglobin subunit delta OS=Homo sapiens OX=9606 GN=HBD PE=1 SV=1
Q9NR34	1	1	4.6312	0.006358	0.007158	109.0938	0.943504	2D	0.3PG	71138.8504	Mannosyl-oligosaccharide 1_2-alpha-mannosidase IC OS=Homo sapiens OX=9606 GN=MAN1C1 PE=1 SV=1
C9JRL4	4	1	35.5438	6.78E-07	2.54E-06	6.800578	1	2D	0.3PG	13144.3775	Cytosolic malate dehydrogenase (Fragment) OS=Homo sapiens OX=9606 GN=MDH1 PE=1 SV=8
J3QRS3;P19105;J3KTJ1;P24844	5	1	38.9528	0.000122	0.000178	65.73267	1	2D	HYS	20513.9808	Myosin regulatory light chain 12A OS=Homo sapiens OX=9606 GN=MYL12A PE=1 SV=1
H0Y5N9	4	1	24.4535	1.14E-07	8.98E-07	5.918098	1	HYS	0.3PG	80456.6982	Collagen alpha-1(XII) chain (Fragment) OS=Homo sapiens OX=9606 GN=COL12A1 PE=1 SV=1
K7EK47	2	1	10.1824	0.010866	0.011887	1.435174	0.886077	2D	HYS	28222.4184	Adhesion G protein-coupled receptor L1 (Fragment) OS=Homo sapiens OX=9606 GN=ADGRL1 PE=1 SV=1
Q14563	3	1	22.3055	0.005206	0.005901	1.910843	0.958383	2D	0.3PG	89973.0135	Semaphorin-3A OS=Homo sapiens OX=9606 GN=SEMA3A PE=1 SV=1
Q96NY7	2	1	11.5215	2.18E-07	1.33E-06	8.681473	1	2D	0.3PG	73239.6893	Chloride intracellular channel protein 6 OS=Homo sapiens OX=9606 GN=CLIC6 PE=2 SV=3
A6NHC0;B1B154	5	1	23.1731	2.04E-05	3.87E-05	5.181733	1	0.3PG	HYS	80171.0646	Calpain-8 OS=Homo sapiens OX=9606 GN=CAPN8 PE=1 SV=3
A0A4W8ZXM2;A0A0B4J2B5;S4R46(	2	1	23.3004	2.39E-07	1.38E-06	4.155868	1	0.3PG	2D	11281.4742	Immunoglobulin heavy variable 3-72 OS=Homo sapiens OX=9606 GN=IGHV3-72 PE=1 SV=1
P59045	2	1	9.8942	0.008105	0.008994	10.20976	0.92079	0.3PG	2D	120060.4805	NACHT_ LRR and PYD domains-containing protein 11 OS=Homo sapiens OX=9606 GN=NLRP11 PE=2 SV=2
Q53HC5	4	1	24.9378	0.000182	0.000253	1.496281	0.999999	0.3PG	HYS	69223.1183	Kelch-like protein 26 OS=Homo sapiens OX=9606 GN=KLHL26 PE=1 SV=2
K7EIX2;Q6AI08	2	1	14.6121	1.49E-05	2.94E-05	8.528238	1	0.3PG	2D	118131.9919	HEAT repeat-containing protein 6 OS=Homo sapiens OX=9606 GN=HEATR6 PE=1 SV=1
P08581	2	1	9.4468	2.52E-05	4.60E-05	4.097841	1	2D	0.3PG	157879.6758	Hepatocyte growth factor receptor OS=Homo sapiens OX=9606 GN=MET PE=1 SV=4
Q12756;A0A3B3IT28;A0A3B3ITE5;A	2	1	4.66	0.000206	0.000282	2.729899	0.999999	HYS	0.3PG	192661.2248	Kinesin-like protein KIF1A OS=Homo sapiens OX=9606 GN=KIF1A PE=1 SV=2
H0Y6R1;H7BZV7;Q9UBP9	2	1	23.4	8.95E-06	1.91E-05	9.863891	1	0.3PG	2D	16665.0083	PTB domain-containing engulfment adapter protein 1 (Fragment) OS=Homo sapiens OX=9606 GN=GULP1 PE=1 SV=1
Q9NZA1;A0A2R8Y4M1;A0A2R8Y61	3	1	15.7474	9.08E-05	0.000138	5.15118	1	2D	HYS	46844.8646	Chloride intracellular channel protein 5 OS=Homo sapiens OX=9606 GN=CLIC5 PE=1 SV=3
P13497	7	1	34.1022	0.100614	0.103413	1.319505	0.429341	2D	HYS	113586.9292	Bone morphogenetic protein 1 OS=Homo sapiens OX=9606 GN=BMP1 PE=1 SV=2
F8WAS2	7	1	53.813	2.86E-06	7.62E-06	1.820332	1	HYS	0.3PG	23551.1583	Inter-alpha-trypsin inhibitor heavy chain H1 OS=Homo sapiens OX=9606 GN=ITIH1 PE=1 SV=1
E7EMV2;E7ESP9;P07197	3	1	37.754	6.43E-05	0.000103	4.456715	1	0.3PG	2D	78938.6449	160 kDa neurofilament protein OS=Homo sapiens OX=9606 GN=NEFM PE=1 SV=1
E9PRU1;H0YET5	2	1	15.9327	0.012806	0.013902	7.171934	0.863055	2D	0.3PG	50586.872	EGF-containing fibulin-like extracellular matrix protein 2 OS=Homo sapiens OX=9606 GN=EFEMP2 PE=1 SV=1
Q9BYZ2	2	1	26.2531	0.000566	0.000713	6.778716	0.999866	0.3PG	HYS	42399.2078	L-lactate dehydrogenase A-like 6B OS=Homo sapiens OX=9606 GN=LDHAL6B PE=1 SV=3
Q969W8;C9J6X1	3	1	17.1759	6.24E-07	2.43E-06	5.599273	1	0.3PG	2D	50416.8835	Zinc finger protein 566 OS=Homo sapiens OX=9606 GN=ZNF566 PE=1 SV=1
Q7Z3Y9	3	1	18.7701	8.60E-06	1.86E-05	13.08461	1	0.3PG	2D	52652.0738	Keratin_ type I cytoskeletal 26 OS=Homo sapiens OX=9606 GN=KRT26 PE=1 SV=2
Q6KB66	3	1	39.1091	9.16E-05	0.000139	13.80796	1	0.3PG	2D	51038.6257	Keratin_ type II cytoskeletal 80 OS=Homo sapiens OX=9606 GN=KRT80 PE=1 SV=2
A0A7I2V649;A0A7I2YQ88;A0A7I2Y(	5	1	31.652	0.028725	0.030368	1.378063	0.715836	HYS	0.3PG	65346.7579	Polyadenylate-binding protein 1 OS=Homo sapiens OX=9606 GN=PABPC1 PE=1 SV=1
P02652;V9GYM3;V9GYE3	2	1	23.7316	1.50E-05	2.96E-05	14.88321	1	0.3PG	HYS	11289.0901	Apolipoprotein A-II OS=Homo sapiens OX=9606 GN=APOA2 PE=1 SV=1
Q9NSB4	2	1	19.4451	7.81E-10	5.10E-08	Infinity	1	0.3PG	2D	58021.442	Keratin_ type II cuticular Hb2 OS=Homo sapiens OX=9606 GN=KRT82 PE=1 SV=3
C4AM86;Q92764	2	1	13.2365	9.99E-09	2.02E-07	11639.29	1	0.3PG	HYS	48846.1951	Keratin_ type I cuticular Ha5 OS=Homo sapiens OX=9606 GN=KRT35 PE=1 SV=1
Q9H668	3	1	22.7339	0.005019	0.005704	1.390354	0.960763	HYS	0.3PG	42461.2598	CST complex subunit STN1 OS=Homo sapiens OX=9606 GN=STN1 PE=1 SV=2
A0A087WY68;A0A087WZR0;A0A7I2	2	1	11.0712	3.12E-05	5.52E-05	2.187355	1	0.3PG	HYS	109194.6009	Proprotein convertase subtilisin/kexin type 6 OS=Homo sapiens OX=9606 GN=PCSK6 PE=1 SV=1
Q7Z3Z0	7	1	63.5963	0.115342	0.118264	1.197261	0.398648	HYS	0.3PG	49888.268	Keratin_ type I cytoskeletal 25 OS=Homo sapiens OX=9606 GN=KRT25 PE=1 SV=1
A0A669KBL2;Q5VUG0;Q5T980;Q5T	3	0	17.8028					---	---	102030.3611	Scm-like with four MBT domains protein 2 OS=Homo sapiens OX=9606 GN=SFMBT2 PE=1 SV=1
A0A0A0MRH0;Q9NVX2	1	0	4.1257					---	---	48911.0673	Notchless protein homolog 1 OS=Homo sapiens OX=9606 GN=NLE1 PE=1 SV=1
A0A0A0MTJ2;E7ENT7;Q9BQ16	1	0	5.5013					---	---	40712.3457	Sparc/osteonectin_ cwcv and kazal-like domains proteoglycan (Testican) 3_ isoform CRA_b OS=Homo sapiens OX=9606 GN=SPOCK3 PE=1 SV=1
B7Z7P8;P62495	1	0	5.265					---	---	47761.272	Eukaryotic peptide chain release factor subunit 1 OS=Homo sapiens OX=9606 GN=ETF1 PE=1 SV=1
Q8WZ73	1	0	3.9928					---	---	41768.6822	E3 ubiquitin-protein ligase rififylin OS=Homo sapiens OX=9606 GN=RFFL PE=1 SV=1
A0A087X1K1;A0A087X1Z5;Q96C00	1	0	5.2811					---	---	15307.6932	Zinc finger and BTB domain-containing protein 9 (Fragment) OS=Homo sapiens OX=9606 GN=ZBTB9 PE=1 SV=1
C9JIF9;P13798	1	0	3.9748					---	---	82757.7449	Acyl-peptide hydrolase OS=Homo sapiens OX=9606 GN=APEH PE=1 SV=1
Q9Y536	3	0	31.4242					---	---	18409.9414	Peptidyl-prolyl cis-trans isomerase A-like 4A OS=Homo sapiens OX=9606 GN=PPIAL4A PE=2 SV=1
Q06278	1	0	5.0338					---	---	150313.3779	Aldehyde oxidase OS=Homo sapiens OX=9606 GN=AOX1 PE=1 SV=2
M0R0Q9	5	0	39.5405					---	---	11268.8066	Complement C3 (Fragment) OS=Homo sapiens OX=9606 GN=C3 PE=1 SV=1

P16435	1	0	4.4946					---	---	77146.0795	NADPH--cytochrome P450 reductase OS=Homo sapiens OX=9606 GN=POR PE=1 SV=2
B1APN9;O75335;M0QZB5	5	0	25.6464					---	---	134497.5335	Liprin-alpha-4 OS=Homo sapiens OX=9606 GN=PPFIA4 PE=1 SV=2
C9JWP9;K7EKU8	1	0	10.5386					---	---	26045.4386	Disks large homolog 4 (Fragment) OS=Homo sapiens OX=9606 GN=DLG4 PE=1 SV=2
F8WC23;H7BZI8;Q6ZW49	1	0	12.4088					---	---	16200.1713	PAX transactivation activation domain-interacting protein OS=Homo sapiens OX=9606 GN=PAXIP1 PE=1 SV=1
O75128;A0A0J9YWK3;A0A3B3IU00	5	0	22.7207					---	---	136529.3892	Protein cordon-bleu OS=Homo sapiens OX=9606 GN=COBL PE=1 SV=2
F8VXC8;F8VZW6;Q8TAQ2	1	0	13.0326					---	---	136583.5417	SWI/SNF complex subunit SMARCC2 OS=Homo sapiens OX=9606 GN=SMARCC2 PE=1 SV=1
H7C0T9;Q9NZQ3	1	0	5.3171					---	---	16382.8316	NCK-interacting protein with SH3 domain (Fragment) OS=Homo sapiens OX=9606 GN=NCKIPSD PE=1 SV=1
P78332	1	0	7.0018					---	---	129271.3871	RNA-binding protein 6 OS=Homo sapiens OX=9606 GN=RBM6 PE=1 SV=5
A0A5H1ZRS9	2	0	14.4601					---	---	11305.7381	Immunoglobulin kappa variable 2D-29 OS=Homo sapiens OX=9606 GN=IGKV2D-29 PE=1 SV=1
O60656	1	0	5.0133					---	---	60796.5599	UDP-glucuronosyltransferase 1A9 OS=Homo sapiens OX=9606 GN=UGT1A9 PE=1 SV=1
A1A4Z5;Q70T25;Q9HCX4	4	0	31.262					---	---	43943.0492	Short transient receptor potential channel 7 OS=Homo sapiens OX=9606 GN=TRPC7 PE=2 SV=1
Q8IWU5;B1AMP9	3	0	16.3313					---	---	101823.4711	Extracellular sulfatase Sulf-2 OS=Homo sapiens OX=9606 GN=SULF2 PE=1 SV=1
P05186	1	0	5.716					---	---	57647.0927	Alkaline phosphatase_ tissue-nonspecific isozyme OS=Homo sapiens OX=9606 GN=ALPL PE=1 SV=4
Q15648;J3QKZ7	3	0	20.131					---	---	169447.8304	Mediator of RNA polymerase II transcription subunit 1 OS=Homo sapiens OX=9606 GN=MED1 PE=1 SV=4
O75695	1	0	8.6486					---	---	40496.5287	Protein XRP2 OS=Homo sapiens OX=9606 GN=RP2 PE=1 SV=4
P0DOY2;P0DOY3	3	0	59.5458					---	---	11464.6553	Immunoglobulin lambda constant 2 OS=Homo sapiens OX=9606 GN=IGLC2 PE=1 SV=1
A0A0G2JLV7;Q6GTX8;A0A0G2JM9	3	0	21.4334					---	---	31756.2833	Leukocyte-associated immunoglobulin-like receptor 1 OS=Homo sapiens OX=9606 GN=LAIR1 PE=1 SV=1
B4DY09;Q12905	3	0	16.7905					---	---	39081.5686	Interleukin enhancer-binding factor 2 OS=Homo sapiens OX=9606 GN=ILF2 PE=1 SV=1
C9JYY6;F8W775;Q92823	1	0	4.4227					---	---	134669.1537	Neuronal cell adhesion molecule OS=Homo sapiens OX=9606 GN=NRCAM PE=1 SV=3
P05452	1	0	4.0269					---	---	22936.0447	Tetranectin OS=Homo sapiens OX=9606 GN=CLEC3B PE=1 SV=3
Q9Y4D7	1	0	4.7971					---	---	215428.9271	Plexin-D1 OS=Homo sapiens OX=9606 GN=PLXND1 PE=1 SV=3
Q7Z3Y7	4	0	39.6594					---	---	51194.7661	Keratin_ type I cytoskeletal 28 OS=Homo sapiens OX=9606 GN=KRT28 PE=1 SV=2
Q9BYX7	4	0	36.2372					---	---	42358.5184	Putative beta-actin-like protein 3 OS=Homo sapiens OX=9606 GN=POTEKP PE=5 SV=1
H0Y2S9	4	0	21.2464					---	---	204685.5406	Myosin phosphatase Rho-interacting protein (Fragment) OS=Homo sapiens OX=9606 GN=MPRIP PE=1 SV=3
E7EVH7;F8W6L3;G3V2E7;G3V3H3;	1	0	4.4312					---	---	83223.8721	Kinesin light chain OS=Homo sapiens OX=9606 PE=3 SV=2
P26010;F5H6T4	4	0	19.407					---	---	90039.8423	Integrin beta-7 OS=Homo sapiens OX=9606 GN=ITGB7 PE=1 SV=1
E7EQG2;Q14240	4	0	31.6533					---	---	41518.6072	RNA helicase OS=Homo sapiens OX=9606 GN=EIF4A2 PE=1 SV=1
A0A0A0MSQ3;Q96PU4	1	0	5.6764					---	---	12911.782	E3 ubiquitin-protein ligase UHRF2 (Fragment) OS=Homo sapiens OX=9606 GN=UHRF2 PE=1 SV=1
D6REN1;D6RGC4;Q8TCQ1	1	0	11.792					---	---	16255.0405	E3 ubiquitin-protein ligase MARCHF1 (Fragment) OS=Homo sapiens OX=9606 GN=MARCHF1 PE=1 SV=1
A0A6Q8PHN5	1	0	6.3799					---	---	5501.161	Alanine--tRNA ligase_ cytoplasmic OS=Homo sapiens OX=9606 GN=AARS1 PE=1 SV=1
Q99574	1	0	5.5124					---	---	46427.0991	Neuroserpin OS=Homo sapiens OX=9606 GN=SERPINI1 PE=1 SV=1
Q9BZA8	1	0	4.3458					---	---	147858.3052	Protocadherin-11 Y-linked OS=Homo sapiens OX=9606 GN=PCDH11Y PE=1 SV=1
Q99250;A0A1B0GW67	3	0	14.789					---	---	230142.4426	Sodium channel protein type 2 subunit alpha OS=Homo sapiens OX=9606 GN=SCN2A PE=1 SV=3
Q99102	1	0	4.8094					---	---	234141.3459	Mucin-4 OS=Homo sapiens OX=9606 GN=MUC4 PE=1 SV=4
K7EJD3	4	0	46.9893					---	---	22622.0702	Galectin-3-binding protein OS=Homo sapiens OX=9606 GN=LGALS3BP PE=1 SV=1
A0A5F9ZHS3;E7EN32;O00255;E7EN	3	0	15.8081					---	---	72831.8112	Menin OS=Homo sapiens OX=9606 GN=MEN1 PE=1 SV=1
H0Y781;H3BLT5;H7C0Y8	3	0	20.8156					---	---	186417.8005	Transmembrane protein KIAA1109 (Fragment) OS=Homo sapiens OX=9606 GN=KIAA1109 PE=1 SV=1
E9PNV5	3	0	23.8658					---	---	85841.1198	Neuron navigator 2 (Fragment) OS=Homo sapiens OX=9606 GN=NAV2 PE=1 SV=8
Q96AP0	1	0	0					---	---	49879.7528	Adrenocortical dysplasia protein homolog OS=Homo sapiens OX=9606 GN=ACD PE=1 SV=4
J3QLC6	1	0	5.6433					---	---	17428.8013	Ras-related protein Rab-34_ isoform NARR (Fragment) OS=Homo sapiens OX=9606 GN=RAB34 PE=1 SV=1
Q9Y471	1	0	5.1807					---	---	58779.1716	Inactive cytidine monophosphate-N-acetylneuraminic acid hydroxylase OS=Homo sapiens OX=9606 GN=CMAHP PE=1 SV=4
Q96C90;F5GXC4;F5H2U0	3	0	23.7379					---	---	16025.0171	Protein phosphatase 1 regulatory subunit 14B OS=Homo sapiens OX=9606 GN=PPP1R14B PE=1 SV=3
E9PL10;Q96K17	1	0	4.9864					---	---	15779.075	Transcription factor BTF3 OS=Homo sapiens OX=9606 GN=BTF3L4 PE=1 SV=1
P16298;Q5F2F8	1	0	4.8957					---	---	59708.7204	Serine/threonine-protein phosphatase 2B catalytic subunit beta isoform OS=Homo sapiens OX=9606 GN=PPP3CB PE=1 SV=2
D3DUE4;F6WF08;K7EJS9;K7ELU8;K	1	0	6.5915					---	---	58826.7658	Ankyrin repeat and SAM domain-containing protein 3 OS=Homo sapiens OX=9606 GN=ANKS3 PE=1 SV=1
Q92520	1	0	5.6771					---	---	24965.6582	Protein FAM3C OS=Homo sapiens OX=9606 GN=FAM3C PE=1 SV=1
Q96D15	1	0	6.1679					---	---	37493.0374	Reticulocalbin-3 OS=Homo sapiens OX=9606 GN=RCN3 PE=1 SV=1
A0A1P0AYU5;Q9BWM7;S4R3N9	1	0	5.8823					---	---	36321.1403	Sideroflexin OS=Homo sapiens OX=9606 GN=SFXN3 PE=1 SV=1
P01782;P0DP04	1	0	5.419					---	---	13115.8541	Immunoglobulin heavy variable 3-9 OS=Homo sapiens OX=9606 GN=IGHV3-9 PE=1 SV=2
M0R366;Q9BTV5	1	0	4.5262					---	---	49706.4482	Fibronectin type III and SPRY domain-containing protein 1 OS=Homo sapiens OX=9606 GN=FSD1 PE=1 SV=1
Q9Y241	1	0	11.1582					---	---	10142.9528	HIG1 domain family member 1A_ mitochondrial OS=Homo sapiens OX=9606 GN=HIGD1A PE=1 SV=1
H0YN44;O60566	1	0	9.7886					---	---	10477.7652	Mitotic checkpoint serine/threonine-protein kinase BUB1 beta OS=Homo sapiens OX=9606 GN=BUB1B PE=1 SV=1
C9JGI3;P19971;A0A494BZZ4;A0A4	2	0	10.7397					---	---	46486.3761	Thymidine phosphorylase (Fragment) OS=Homo sapiens OX=9606 GN=TYMP PE=1 SV=1
P12882	9	0	50.809					---	---	224115.3216	Myosin-1 OS=Homo sapiens OX=9606 GN=MYH1 PE=1 SV=3
B7ZLG1;Q9BZS1;A0A0C4DFW6	2	0	9.2342					---	---	50268.4427	FOXP3 protein OS=Homo sapiens OX=9606 GN=FOXP3 PE=1 SV=1
A0A0C4DFX9;H0Y3X6;Q9H3P2	2	0	12.7366					---	---	58727.5091	Negative elongation factor A OS=Homo sapiens OX=9606 GN=NELFA PE=1 SV=1
O60885	2	0	7.4444					---	---	152675.2472	Bromodomain-containing protein 4 OS=Homo sapiens OX=9606 GN=BRD4 PE=1 SV=2
A0A0B4J1X5;A0A0C4DH42;P01767	2	0	27.6652					---	---	13010.7201	Immunoglobulin heavy variable 3-74 OS=Homo sapiens OX=9606 GN=IGHV3-74 PE=3 SV=1
P61769;F5H6I0	6	0	76.8088					---	---	13828.6371	Beta-2-microglobulin OS=Homo sapiens OX=9606 GN=B2M PE=1 SV=1
A0A140T975;A0A1W2PS39;Q5RJ27	2	0	11.3819					---	---	34549.5519	HLA class I histocompatibility antigen_ A alpha chain OS=Homo sapiens OX=9606 GN=HLA-A PE=1 SV=1
P08779;C9JM50;K7EPJ9	10	0	82.8931					---	---	51610.0737	Keratin_ type I cytoskeletal 16 OS=Homo sapiens OX=9606 GN=KRT16 PE=1 SV=4
A6NKB8;Q9H4A4	2	0	14.5205					---	---	68796.1618	Aminopeptidase B OS=Homo sapiens OX=9606 GN=RNPEP PE=1 SV=1
D3YTG3;H7C556;F6R962;H0Y897;E	10	0	87.7684					---	---	197059.4754	Target of Nesh-SH3 OS=Homo sapiens OX=9606 GN=ABI3BP PE=1 SV=2
E7EVK1	2	0	15.1072					---	---	116488.5296	Nuclear receptor corepressor 1 (Fragment) OS=Homo sapiens OX=9606 GN=NCOR1 PE=1 SV=2
Q8ND30	2	0	10.5559					---	---	99114.1055	Liprin-beta-2 OS=Homo sapiens OX=9606 GN=PPFIBP2 PE=1 SV=3
P48668	12	0	90.4858					---	---	60310.174	Keratin_ type II cytoskeletal 6C OS=Homo sapiens OX=9606 GN=KRT6C PE=1 SV=3
G3V5M2;P00491	2	0	17.8501					---	---	24592.9423	Inosine-guanosine phosphorylase (Fragment) OS=Homo sapiens OX=9606 GN=PNP PE=1 SV=1
P02538	12	0	83.3137					---	---	60330.1643	Keratin_ type II cytoskeletal 6A OS=Homo sapiens OX=9606 GN=KRT6A PE=1 SV=3
P22392;O60361;C9K028;E5RHP0;F	6	0	35.1254					---	---	17412.1281	Nucleoside diphosphate kinase B OS=Homo sapiens OX=9606 GN=NME2 PE=1 SV=1
A0A7P0T9D6;I3L514	11	0	80.2498					---	---	59577.9718	Protein disulfide-isomerase OS=Homo sapiens OX=9606 GN=P4HB PE=4 SV=1
A0A0C4DFP4;A0A6Q8PF23;Q96IV0	2	0	10.8469					---	---	72981.2595	N-glycanase 1 (Fragment) OS=Homo sapiens OX=9606 GN=NGLY1 PE=1 SV=1
A5A3E0	19	0	180.6814					---	---	123098.626	POTE ankyrin domain family member F OS=Homo sapiens OX=9606 GN=POTEF PE=1 SV=2

Q8WVV5;C9IZY2;H7C4E8	2	0	10.855					---	---	59754.6845	Butyrophilin subfamily 2 member A2 OS=Homo sapiens OX=9606 GN=BTN2A2 PE=1 SV=2
F5GX23;F5H5V4;F5H7X1;J3KN29;O	2	0	10.6669					---	---	20025.5881	26S proteasome non-ATPase regulatory subunit 9 OS=Homo sapiens OX=9606 GN=PSMD9 PE=1 SV=1
Q96BJ3	2	0	10.2521					---	---	35194.2857	Axin interactor_ dorsalization-associated protein OS=Homo sapiens OX=9606 GN=AIDA PE=1 SV=1
Q14525	2	0	19.1002					---	---	47354.2458	Keratin_ type I cuticular Ha3-II OS=Homo sapiens OX=9606 GN=KRT33B PE=1 SV=3
I3L392	7	0	61.4042					---	---	18644.2251	Collagen alpha-3(VI) chain (Fragment) OS=Homo sapiens OX=9606 GN=COL6A3 PE=1 SV=1
Q7Z794	2	0	23.209					---	---	62186.6485	Keratin_ type II cytoskeletal 1b OS=Homo sapiens OX=9606 GN=KRT77 PE=1 SV=3
A0A2R8Y7X9;P69891;P69892	2	0	21.4067					---	---	18477.2286	GLOBIN domain-containing protein OS=Homo sapiens OX=9606 PE=3 SV=1
A0A0B4J1V0	2	0	19.8					---	---	13096.889	Immunoglobulin heavy variable 3-15 OS=Homo sapiens OX=9606 GN=IGHV3-15 PE=3 SV=1
H3BM52	2	0	9.6527					---	---	19746.2765	ADP-ribosylation factor-like protein 2-binding protein (Fragment) OS=Homo sapiens OX=9606 GN=ARL2BP PE=1 SV=1
P14550	2	0	17.4303					---	---	36915.2543	Aldo-keto reductase family 1 member A1 OS=Homo sapiens OX=9606 GN=AKR1A1 PE=1 SV=3
P68363	7	0	56.0358					---	---	50836.0402	Tubulin alpha-1B chain OS=Homo sapiens OX=9606 GN=TUBA1B PE=1 SV=1
F5H5D3;Q9BQE3;F8VVB9;A0A1W2	7	0	55.3017					---	---	58642.6998	Tubulin alpha chain OS=Homo sapiens OX=9606 GN=TUBA1C PE=1 SV=1
A0A087WUE4;C9JMY0;Q96PV6;A0A	2	0	18.6377					---	---	96629.0884	Leukocyte receptor cluster member 8 OS=Homo sapiens OX=9606 GN=LENG8 PE=1 SV=1
Q71U36;Q6PEY2	7	0	55.8198					---	---	50820.0409	Tubulin alpha-1A chain OS=Homo sapiens OX=9606 GN=TUBA1A PE=1 SV=1
Q5TCU3;A7XZE4;P07951;H0YKP3	7	0	47.5486					---	---	32928.7493	Tropomyosin beta chain OS=Homo sapiens OX=9606 GN=TPM2 PE=1 SV=1
A0A087X232	20	0	181.8458					---	---	77445.3502	Complement C1s subcomponent OS=Homo sapiens OX=9606 GN=C1S PE=1 SV=1
B5MC51	2	0	10.9858					---	---	72276.6288	LIM domain kinase 2 OS=Homo sapiens OX=9606 GN=LIMK2 PE=1 SV=1
P01780;A0A0J9YVY3;P01762;P017	2	0	22.0163					---	---	13113.8351	Immunoglobulin heavy variable 3-7 OS=Homo sapiens OX=9606 GN=IGHV3-7 PE=1 SV=2
A0A0G2JMB2	8	0	82.6619					---	---	37306.6938	Immunoglobulin heavy constant alpha 2 (Fragment) OS=Homo sapiens OX=9606 GN=IGHA2 PE=1 SV=1
P55290	2	0	14.3379					---	---	78743.1811	Cadherin-13 OS=Homo sapiens OX=9606 GN=CDH13 PE=1 SV=1
A0A087X256;Q2M389	2	0	13.9655					---	---	137529.2944	WASH complex subunit 4 OS=Homo sapiens OX=9606 GN=WASHC4 PE=1 SV=1
A4D0Y5	2	0	16.2513					---	---	10273.7185	Uncharacterized protein C7orf77 OS=Homo sapiens OX=9606 GN=C7orf77 PE=4 SV=1
Q8N584;G3V1P2	2	0	11.1832					---	---	66839.3215	Tetratricopeptide repeat protein 39C OS=Homo sapiens OX=9606 GN=TTC39C PE=2 SV=2
Q07869	2	0	8.9525					---	---	53308.7581	Peroxisome proliferator-activated receptor alpha OS=Homo sapiens OX=9606 GN=PPARA PE=1 SV=2
E9PI65	8	0	82.7611					---	---	17973.3744	Heat shock cognate 71 kDa protein (Fragment) OS=Homo sapiens OX=9606 GN=HSPA8 PE=1 SV=1
A0A2Q2TTZ9	2	0	26.0635					---	---	11956.147	Immunoglobulin kappa variable 1-33 OS=Homo sapiens OX=9606 GN=IGKV1D-33 PE=1 SV=1
F8WE98	8	0	68.7584					---	---	67047.8709	Filamin-A (Fragment) OS=Homo sapiens OX=9606 GN=FLNA PE=1 SV=2
Q7Z3D4	2	0	13.2957					---	---	34822.7935	LysM and putative peptidoglycan-binding domain-containing protein 3 OS=Homo sapiens OX=9606 GN=LYSMD3 PE=1 SV=2
Q6PD62	2	0	9.6293					---	---	134414.9289	RNA polymerase-associated protein CTR9 homolog OS=Homo sapiens OX=9606 GN=CTR9 PE=1 SV=1
Q9Y2K2	2	0	15.1364					---	---	146106.2208	Serine/threonine-protein kinase SIK3 OS=Homo sapiens OX=9606 GN=SIK3 PE=1 SV=4
H0YJG4	3	0	8.5877					---	---	209392.1952	Chromodomain-helicase-DNA-binding protein 8 (Fragment) OS=Homo sapiens OX=9606 GN=CHD8 PE=1 SV=2
I3L2X3;I3L393;I3NI39;O14978	1	0	5.9512					---	---	26684.2896	Zinc finger protein 263 OS=Homo sapiens OX=9606 GN=ZNF263 PE=1 SV=1
A0A5F9ZGS5;A0A5F9ZGS7;A0A5F9	1	0	4.4154					---	---	207126.5829	Ankyrin-2 OS=Homo sapiens OX=9606 GN=ANK2 PE=1 SV=1
E9PJ66;E9PJY1;E9PL18;Q9NVX7	1	0	7.893					---	---	18677.0326	Kelch repeat and BTB domain-containing protein 4 (Fragment) OS=Homo sapiens OX=9606 GN=KBTBD4 PE=1 SV=1
A0A0A0MQW1;A0A2R8Y4X0;A0A2	1	0	4.4886					---	---	95476.8363	Kinase suppressor of Ras 1 OS=Homo sapiens OX=9606 GN=KSR1 PE=1 SV=1
Q6P0N0	1	0	8.5456					---	---	129998.0722	Mis18-binding protein 1 OS=Homo sapiens OX=9606 GN=MIS18BP1 PE=1 SV=1
E9PIW2	1	0	4.3298					---	---	91044.8919	Disks large homolog 2 OS=Homo sapiens OX=9606 GN=DLG2 PE=1 SV=1
H3BMH0	1	0	5.4245					---	---	29719.6522	CCR4-NOT transcription complex subunit 1 (Fragment) OS=Homo sapiens OX=9606 GN=CNOT1 PE=1 SV=1
Q8N5U0	1	0	5.2124					---	---	36652.9213	Uncharacterized protein C11orf42 OS=Homo sapiens OX=9606 GN=C11orf42 PE=2 SV=2
E7ENX6;Q6PJG9	1	0	5.184					---	---	44965.0599	Leucine-rich repeat and fibronectin type-III domain-containing protein 4 OS=Homo sapiens OX=9606 GN=LRFN4 PE=1 SV=2
D6R934;P02746	2	0	22.3488					---	---	26687.5424	Complement C1q subcomponent subunit B OS=Homo sapiens OX=9606 GN=C1QB PE=1 SV=1
P08133	1	0	5.3055					---	---	76215.5931	Annexin A6 OS=Homo sapiens OX=9606 GN=ANXA6 PE=1 SV=3
A0A1B0GVW0	1	0	11.0232					---	---	38386.351	ATPase H(+)-transporting lysosomal accessory protein 2 OS=Homo sapiens OX=9606 GN=ATP6AP2 PE=1 SV=1
P35503	1	0	6.2474					---	---	60908.7467	UDP-glucuronosyltransferase 1A3 OS=Homo sapiens OX=9606 GN=UGT1A3 PE=1 SV=1
H7C358	1	0	5.7172					---	---	30655.6571	Gamma-tubulin complex component (Fragment) OS=Homo sapiens OX=9606 GN=TUBGCP6 PE=1 SV=1
O95399;Q5H8X8	1	0	5.2966					---	---	14523.7629	Urotensin-2 OS=Homo sapiens OX=9606 GN=UTS2 PE=1 SV=1
Q9UJQ1	1	0	5.5211					---	---	31700.3068	Lysosome-associated membrane glycoprotein 5 OS=Homo sapiens OX=9606 GN=LAMP5 PE=1 SV=1
Q9NQX4	3	0	14.8968					---	---	204122.4111	Unconventional myosin-Vc OS=Homo sapiens OX=9606 GN=MYO5C PE=1 SV=2
A0A2R8Y661;A0A2R8YGD3;Q9Y4G	1	0	5.1098					---	---	175157.9839	Cyclic nucleotide ras GEF OS=Homo sapiens OX=9606 GN=RAPGEF2 PE=1 SV=1
Q8WUF8	1	0	4.3187					---	---	48200.5876	Cotranscriptional regulator FAM172A OS=Homo sapiens OX=9606 GN=FAM172A PE=1 SV=1
C9J4H0;Q9Y6J8	1	0	5.1308					---	---	32449.8751	Serine/threonine/tyrosine-interacting-like protein 1 OS=Homo sapiens OX=9606 GN=STYXL1 PE=1 SV=1
Q15468	1	0	8.7878					---	---	144950.7239	SCL-interrupting locus protein OS=Homo sapiens OX=9606 GN=STIL PE=1 SV=2
A0A6Q8PF40;A0A6Q8PFC4;A0A6Q	1	0	12.075					---	---	41039.4389	Protein TFG OS=Homo sapiens OX=9606 GN=TFG PE=1 SV=1
E9PQV1;P17020	1	0	5.0163					---	---	24725.8087	Zinc finger protein 16 (Fragment) OS=Homo sapiens OX=9606 GN=ZNF16 PE=1 SV=1
Q6ZMR3;A0A087WUM2	3	0	20.9007					---	---	36849.5512	L-lactate dehydrogenase A-like 6A OS=Homo sapiens OX=9606 GN=LDHAL6A PE=2 SV=1
F5H061;P19438	1	0	3.7925					---	---	47111.3649	Tumor necrosis factor receptor superfamily member 1A OS=Homo sapiens OX=9606 GN=TNFRSF1A PE=1 SV=1
H0YJ27	1	0	12.1392					---	---	6533.4923	Protein FAM71D OS=Homo sapiens OX=9606 GN=FAM71D PE=4 SV=2
A0A1W2PQ58;A0A1W2PRD1;A0A1	2	0	9.5686					---	---	76897.163	Sodium channel protein type 3 subunit alpha OS=Homo sapiens OX=9606 GN=SCN3A PE=1 SV=1
O75578	2	0	10.4876					---	---	128970.9391	Integrin alpha-10 OS=Homo sapiens OX=9606 GN=ITGA10 PE=2 SV=2
Q32Q12;E7ERL0;J3KPD9	6	0	41.0252					---	---	32927.1877	Nucleoside diphosphate kinase OS=Homo sapiens OX=9606 GN=NME1-NME2 PE=1 SV=1
H0YLF3	6	0	64.6068					---	---	8555.5371	Beta-2-microglobulin (Fragment) OS=Homo sapiens OX=9606 GN=B2M PE=1 SV=1
F8WEC0;Q9H3L0	2	0	8.7995					---	---	37117.4784	Cobalamin-trafficking protein CblD OS=Homo sapiens OX=9606 GN=MMADHC PE=1 SV=1
H7C4N2	6	0	44.3929					---	---	21860.9404	Plastin-3 (Fragment) OS=Homo sapiens OX=9606 GN=PLS3 PE=1 SV=1
H9KV75	34	0	339.643					---	---	95339.2438	Alpha-actinin-1 OS=Homo sapiens OX=9606 GN=ACTN1 PE=1 SV=1
A0A7I2V4Y4	37	0	373.7192					---	---	104176.9612	Alpha-actinin-1 OS=Homo sapiens OX=9606 GN=ACTN1 PE=1 SV=1
A0A0G2JPR0;A0A140TA32;A0A140	38	0	334.2563					---	---	194472.6355	C4a anaphylatoxin OS=Homo sapiens OX=9606 GN=C4A PE=1 SV=1
A0A087WZN9;A0A087WTR6;A0A0	5	0	28.8443					---	---	199520.4226	Protocadherin-15 OS=Homo sapiens OX=9606 GN=PCDH15 PE=1 SV=1
I3L0U4;I3NI25;Q96NB1	1	0	4.5742					---	---	14020.0385	Centrosomal protein 20 (Fragment) OS=Homo sapiens OX=9606 GN=CEP20 PE=1 SV=1
A0A087X2A7;A0A0C4DGZ2;H0YBA	2	0	9.1349					---	---	128722.4516	Protein FAM184A OS=Homo sapiens OX=9606 GN=FAM184A PE=1 SV=1
A0A3B3ITS5;Q14181	1	0	9.5656					---	---	65258.5239	DNA polymerase alpha subunit B OS=Homo sapiens OX=9606 PE=3 SV=1
M0R2Z9;Q8IX01	3	0	13.1854					---	---	122457.1901	SURP and G-patch domain-containing protein 2 OS=Homo sapiens OX=9606 GN=SUGP2 PE=1 SV=1
Q99489	1	0	4.6936					---	---	38048.2242	D-aspartate oxidase OS=Homo sapiens OX=9606 GN=DDO PE=1 SV=1

A0A087X074;A0A1W2PR54;A0A1W	1	0	10.2058					---	---	17687.929	Spindlin-2A OS=Homo sapiens OX=9606 GN=SPIN2A PE=3 SV=1
Q9UK61	5	0	25.7491					---	---	190571.5125	Protein TASOR OS=Homo sapiens OX=9606 GN=TASOR PE=1 SV=3
A0A0A0MS31;C9JB29	1	0	9.5152					---	---	32772.5624	Caspase-8 OS=Homo sapiens OX=9606 GN=CASP8 PE=1 SV=1
A0A0C4DG76;Q9BQS7;Q5JZ08	5	0	38.0765					---	---	131621.6739	Hephaestin OS=Homo sapiens OX=9606 GN=HEPH PE=1 SV=1
P01889	1	0	5.2517					---	---	40802.2893	HLA class I histocompatibility antigen_ B alpha chain OS=Homo sapiens OX=9606 GN=HLA-B PE=1 SV=3
A0A2R8YF94	1	0	11.0622					---	---	4793.5214	RIMS-binding protein 2 (Fragment) OS=Homo sapiens OX=9606 GN=RIMBP2 PE=4 SV=1
Q9HB75	1	0	5.4913					---	---	100681.2844	p53-induced death domain-containing protein 1 OS=Homo sapiens OX=9606 GN=PIDD1 PE=1 SV=2
P09493;A0A0S2Z4G6;H0YKX5;Q6ZN	5	0	28.3919					---	---	32765.6444	Tropomyosin alpha-1 chain OS=Homo sapiens OX=9606 GN=TPM1 PE=1 SV=2
P55287	4	0	18.3959					---	---	88421.6516	Cadherin-11 OS=Homo sapiens OX=9606 GN=CDH11 PE=2 SV=2
